# Supplementary figures and images for: Threat of shock increases excitability and connectivity of the intraparietal sulcus
Source: eLife. 2017 May 30;6:e23608. doi: 10.7554/eLife.23608 (PMC5478270; doi:10.7554/eLife.23608)

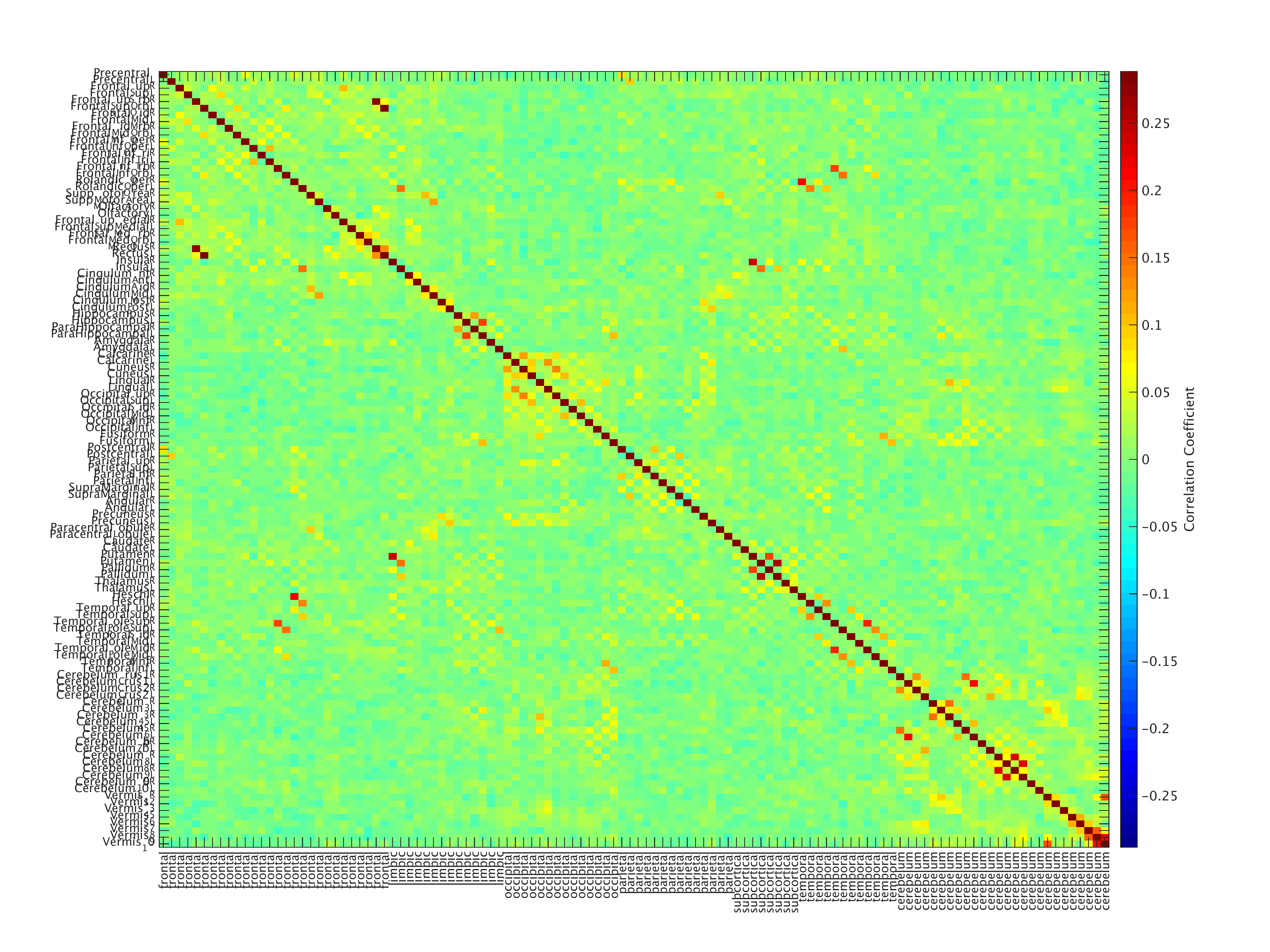

Supplement: Supplementary file 1. — This zip file contains high resolution images of the adjacency matrices for the MEG connectivity analysis suggested by the editor and reviewers. DOI: http://dx.doi.org/10.7554/eLife.23608.021 [file elife-23608-supp1.zip › hi-res_adjacency_matrices/alpha/downsampled/raw/alpha.ave.aal.saf.raw.r.downsampled.png]

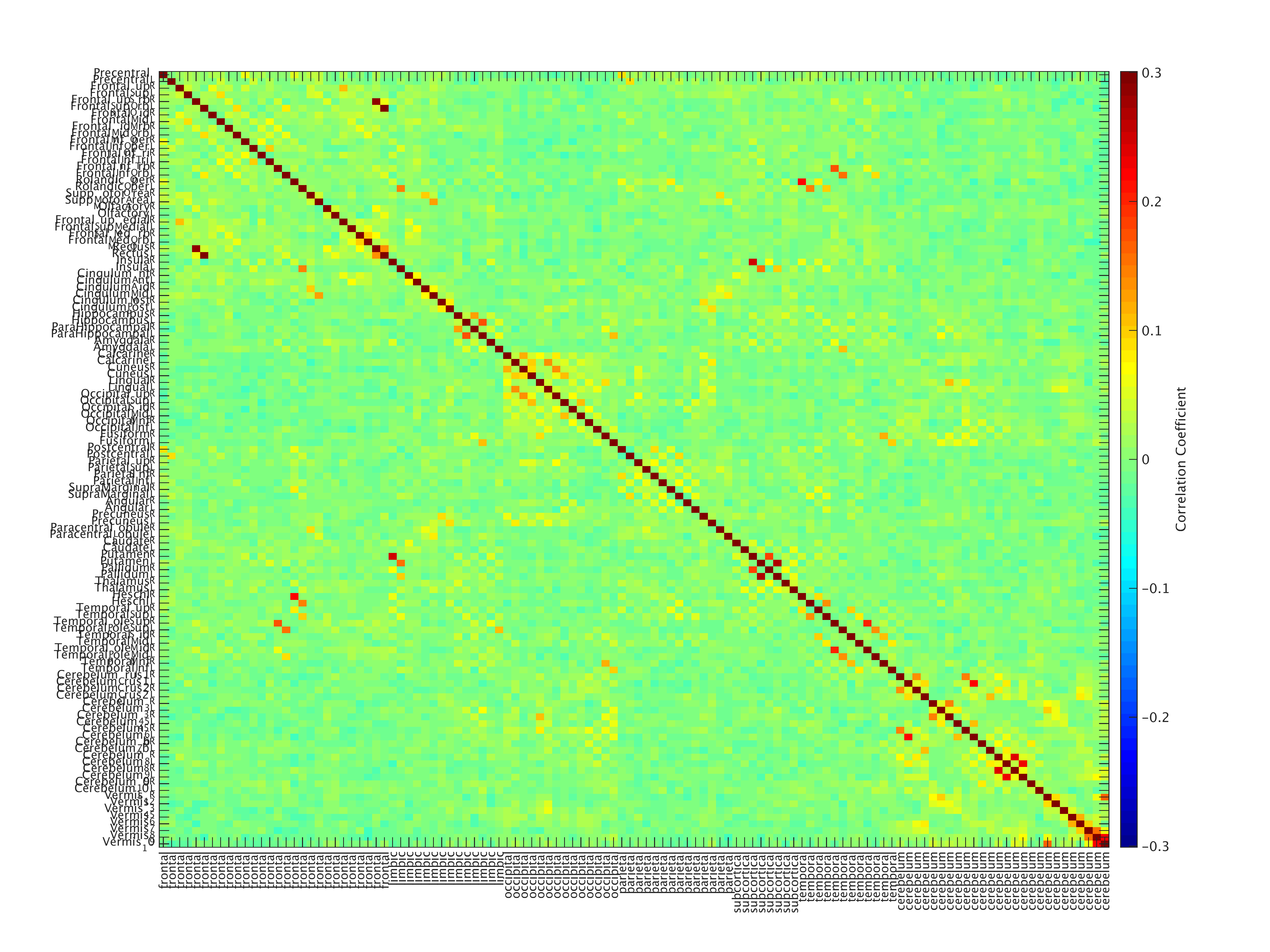

Supplement: Supplementary file 1. — This zip file contains high resolution images of the adjacency matrices for the MEG connectivity analysis suggested by the editor and reviewers. DOI: http://dx.doi.org/10.7554/eLife.23608.021 [file elife-23608-supp1.zip › hi-res_adjacency_matrices/alpha/downsampled/raw/alpha.ave.aal.saf.raw.z.downsampled.png]

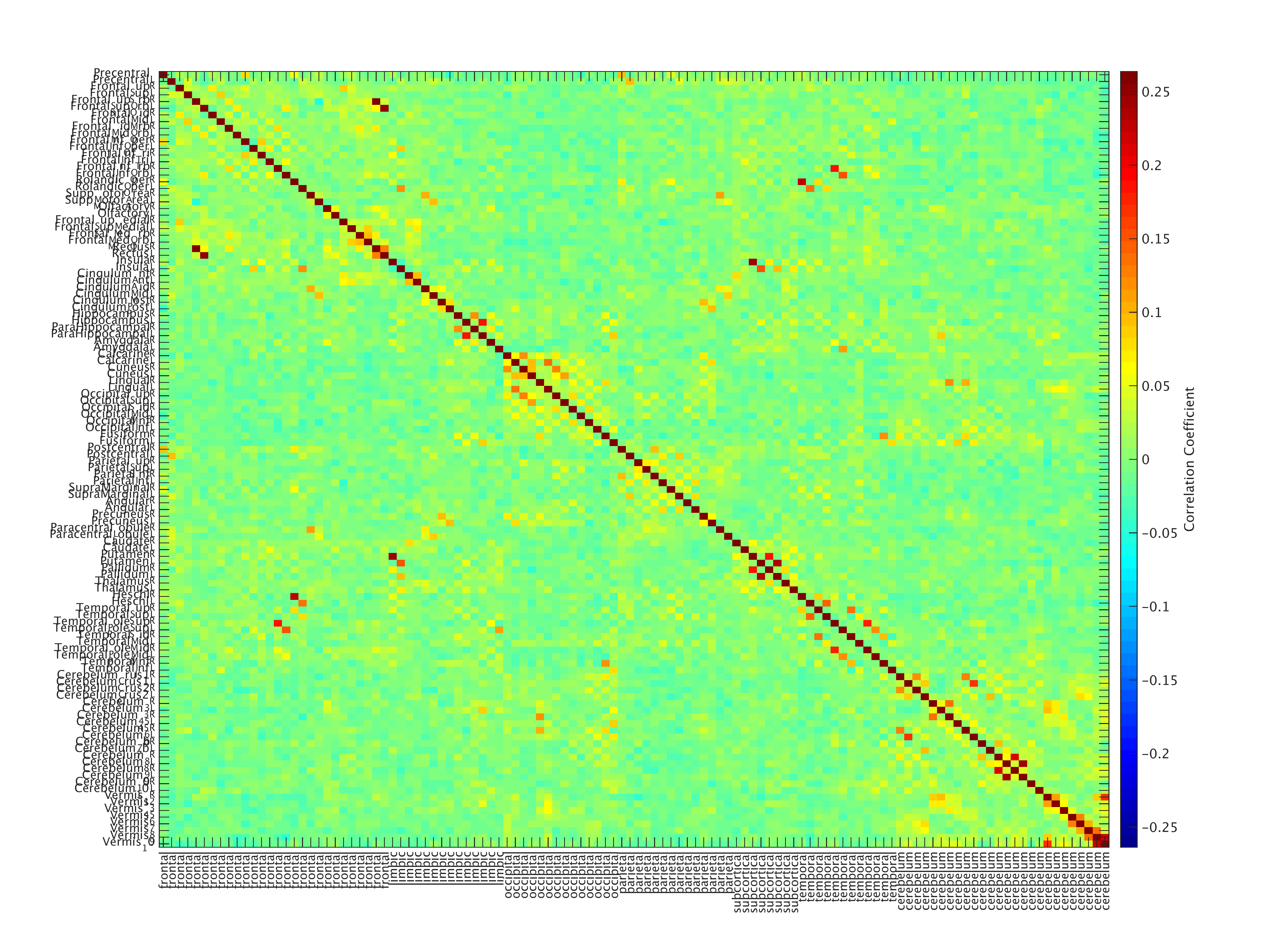

Supplement: Supplementary file 1. — This zip file contains high resolution images of the adjacency matrices for the MEG connectivity analysis suggested by the editor and reviewers. DOI: http://dx.doi.org/10.7554/eLife.23608.021 [file elife-23608-supp1.zip › hi-res_adjacency_matrices/alpha/downsampled/raw/alpha.ave.aal.thr.raw.r.downsampled.png]

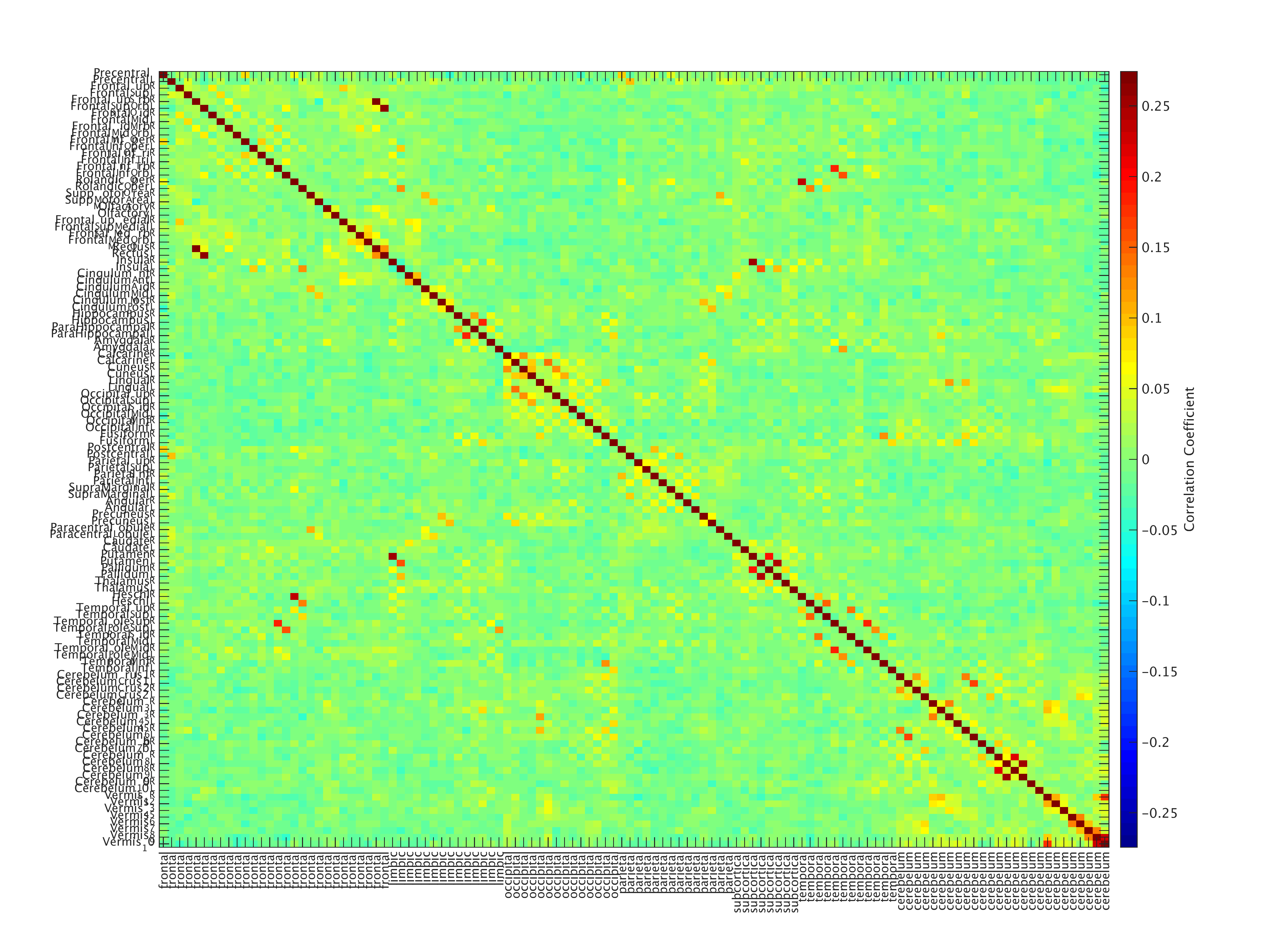

Supplement: Supplementary file 1. — This zip file contains high resolution images of the adjacency matrices for the MEG connectivity analysis suggested by the editor and reviewers. DOI: http://dx.doi.org/10.7554/eLife.23608.021 [file elife-23608-supp1.zip › hi-res_adjacency_matrices/alpha/downsampled/raw/alpha.ave.aal.thr.raw.z.downsampled.png]

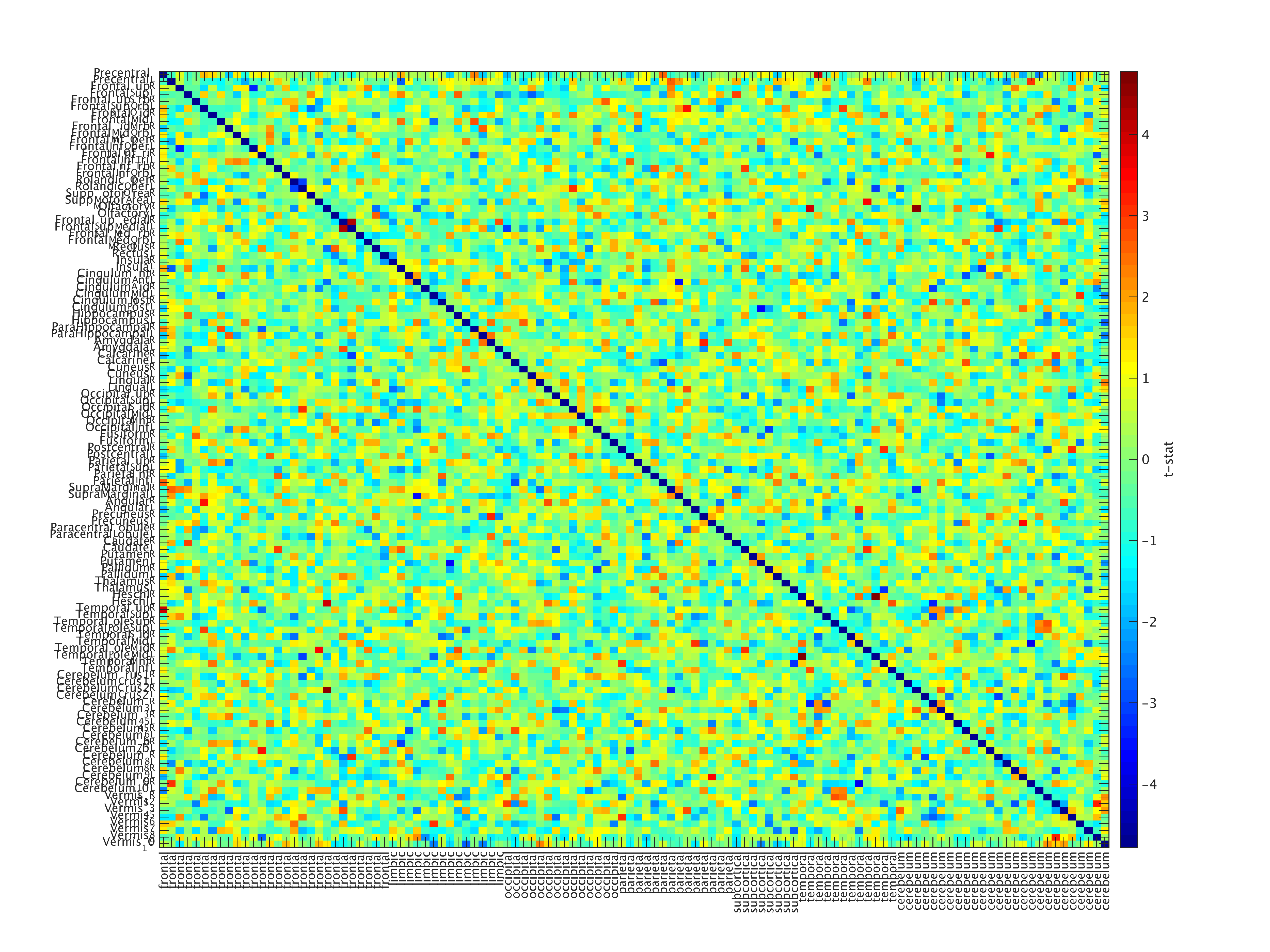

Supplement: Supplementary file 1. — This zip file contains high resolution images of the adjacency matrices for the MEG connectivity analysis suggested by the editor and reviewers. DOI: http://dx.doi.org/10.7554/eLife.23608.021 [file elife-23608-supp1.zip › hi-res_adjacency_matrices/alpha/downsampled/raw/alpha.tstat.aal.raw.r.downsampled.png]

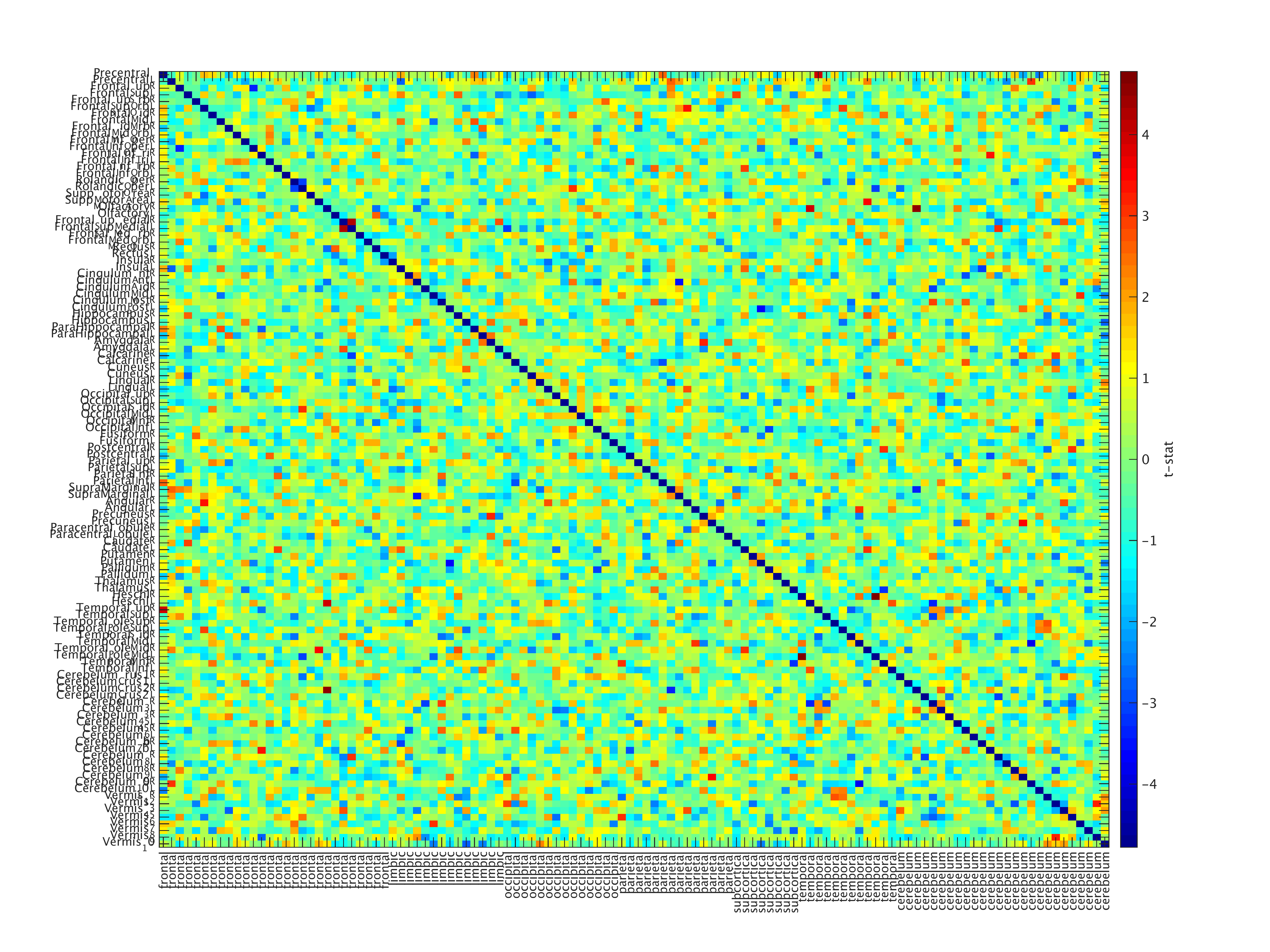

Supplement: Supplementary file 1. — This zip file contains high resolution images of the adjacency matrices for the MEG connectivity analysis suggested by the editor and reviewers. DOI: http://dx.doi.org/10.7554/eLife.23608.021 [file elife-23608-supp1.zip › hi-res_adjacency_matrices/alpha/downsampled/raw/alpha.tstat.aal.raw.z.downsampled.png]

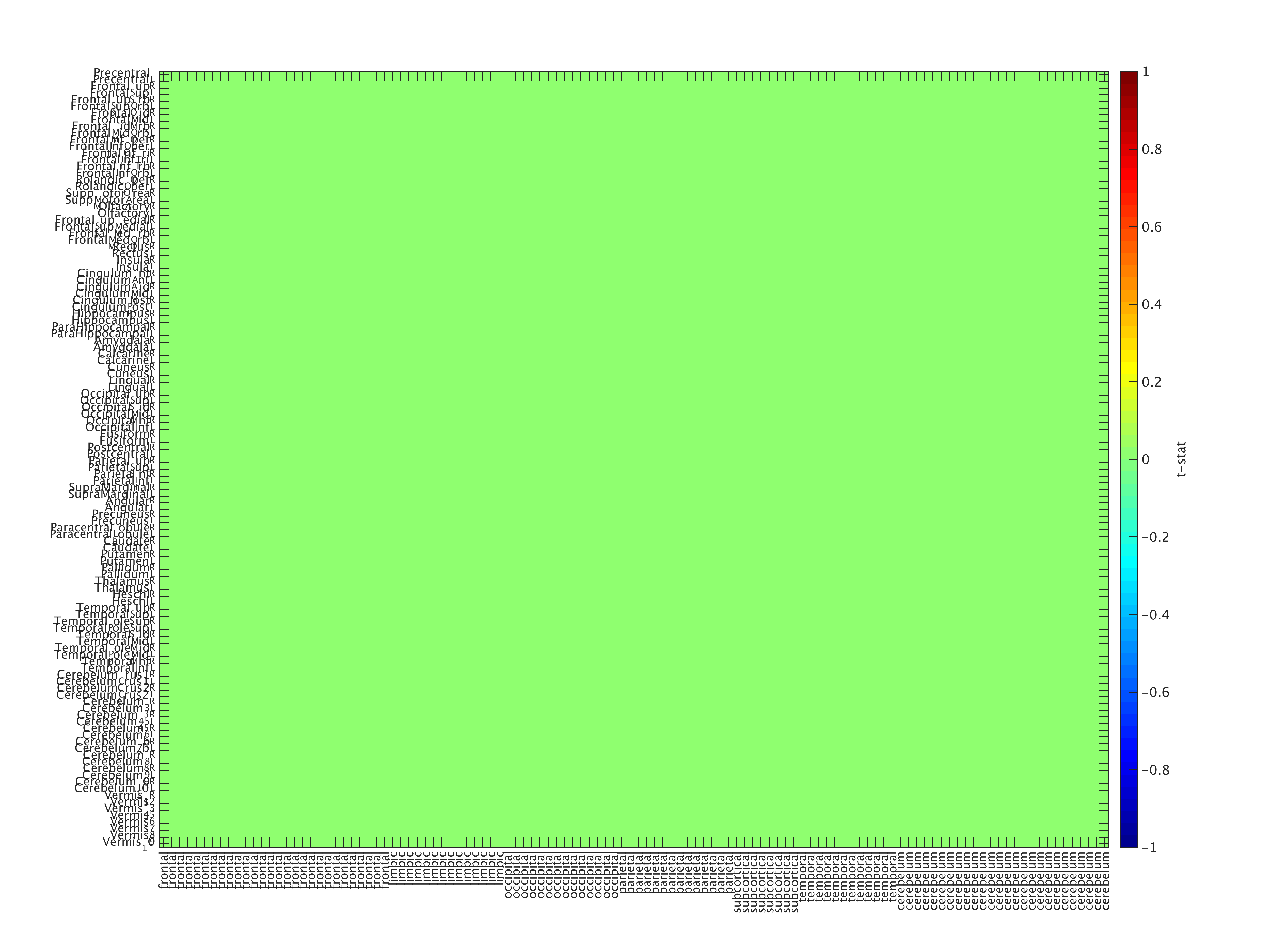

Supplement: Supplementary file 1. — This zip file contains high resolution images of the adjacency matrices for the MEG connectivity analysis suggested by the editor and reviewers. DOI: http://dx.doi.org/10.7554/eLife.23608.021 [file elife-23608-supp1.zip › hi-res_adjacency_matrices/alpha/downsampled/raw/alpha.t-thresh.aal.raw.r.downsampled.png]

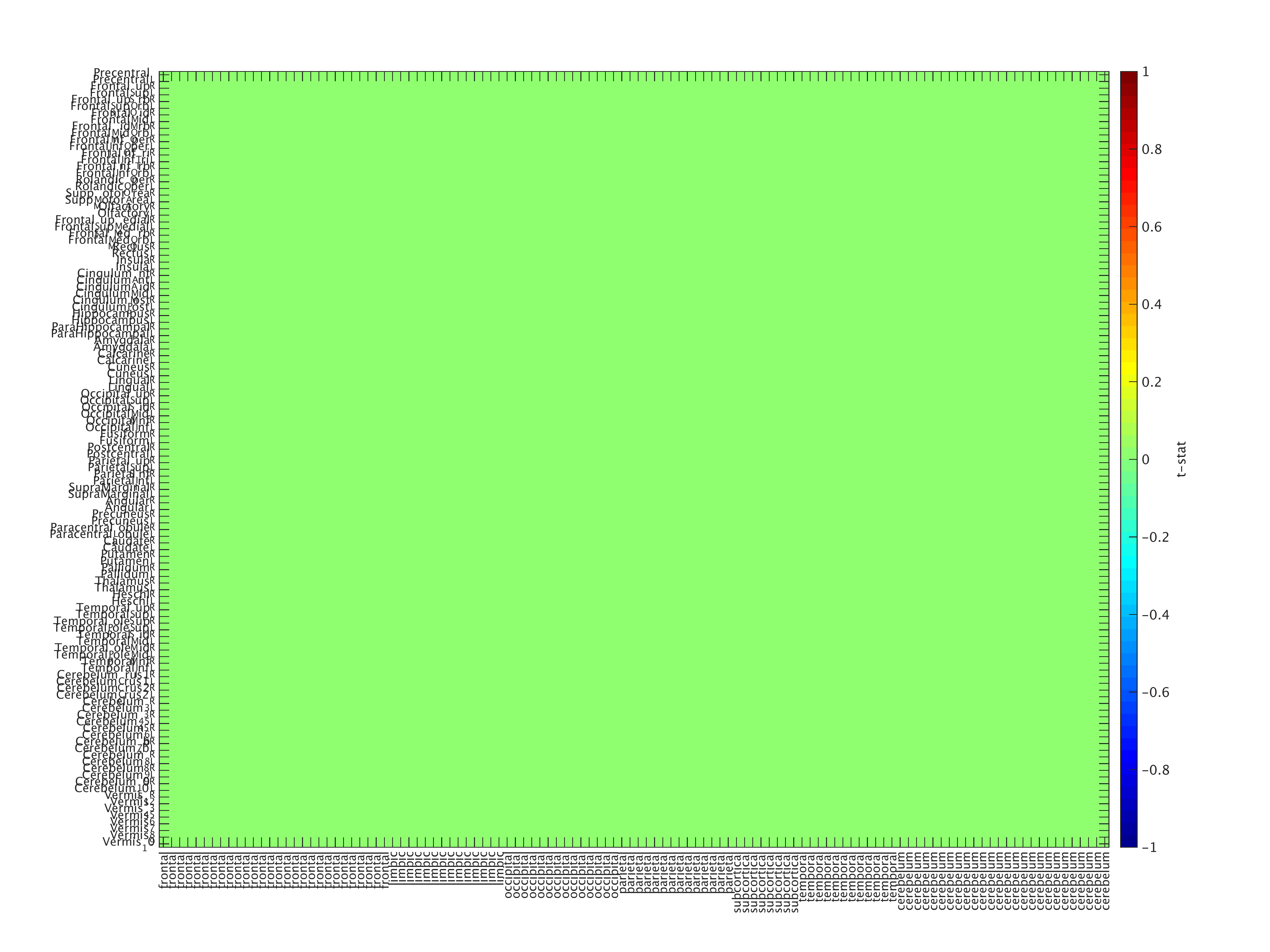

Supplement: Supplementary file 1. — This zip file contains high resolution images of the adjacency matrices for the MEG connectivity analysis suggested by the editor and reviewers. DOI: http://dx.doi.org/10.7554/eLife.23608.021 [file elife-23608-supp1.zip › hi-res_adjacency_matrices/alpha/downsampled/raw/alpha.t-thresh.aal.raw.z.downsampled.png]

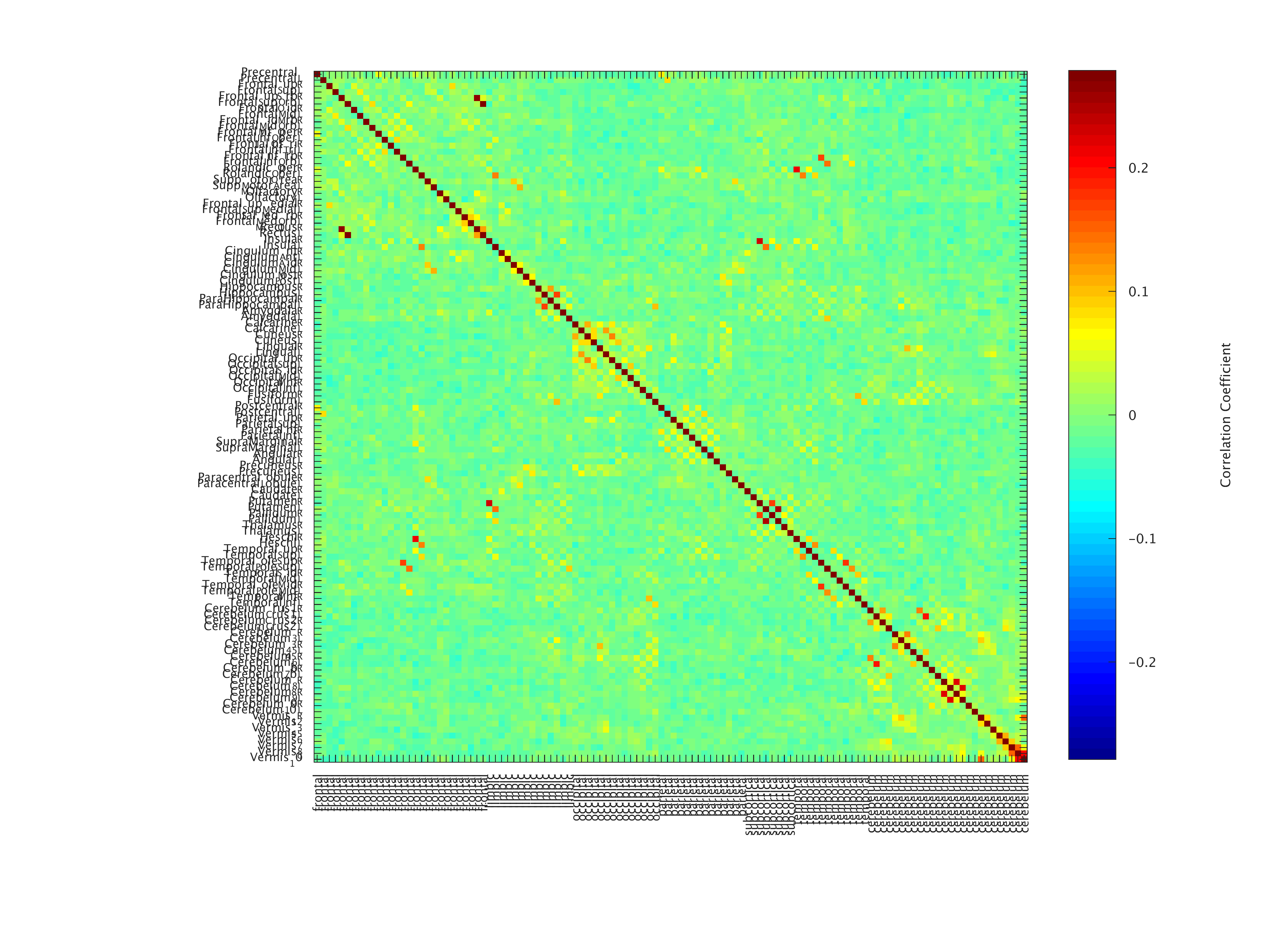

Supplement: Supplementary file 1. — This zip file contains high resolution images of the adjacency matrices for the MEG connectivity analysis suggested by the editor and reviewers. DOI: http://dx.doi.org/10.7554/eLife.23608.021 [file elife-23608-supp1.zip › hi-res_adjacency_matrices/alpha/downsampled/zscore/alpha.ave.aal.saf.zscore.r.downsampled.png]

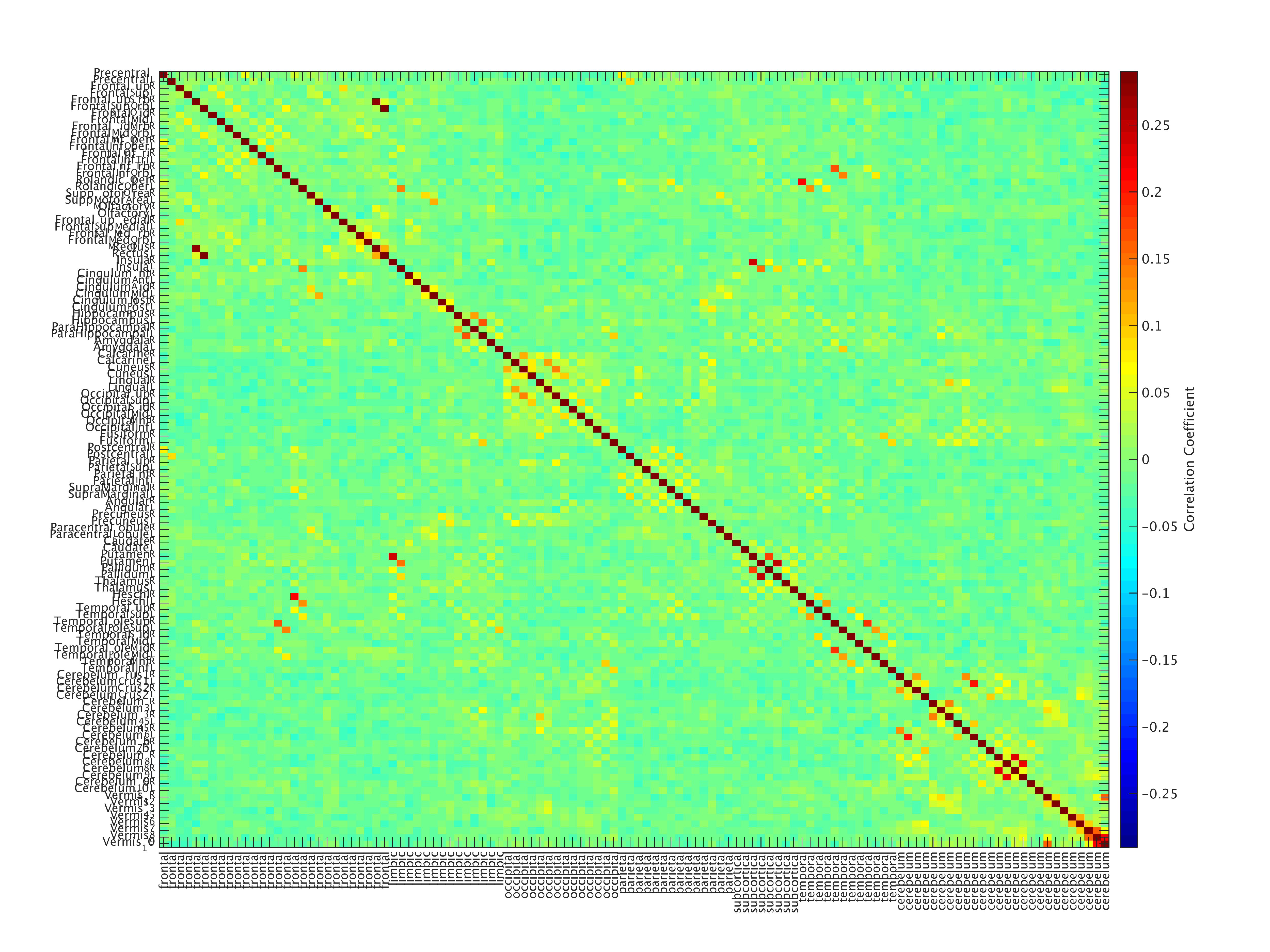

Supplement: Supplementary file 1. — This zip file contains high resolution images of the adjacency matrices for the MEG connectivity analysis suggested by the editor and reviewers. DOI: http://dx.doi.org/10.7554/eLife.23608.021 [file elife-23608-supp1.zip › hi-res_adjacency_matrices/alpha/downsampled/zscore/alpha.ave.aal.saf.zscore.z.downsampled.png]

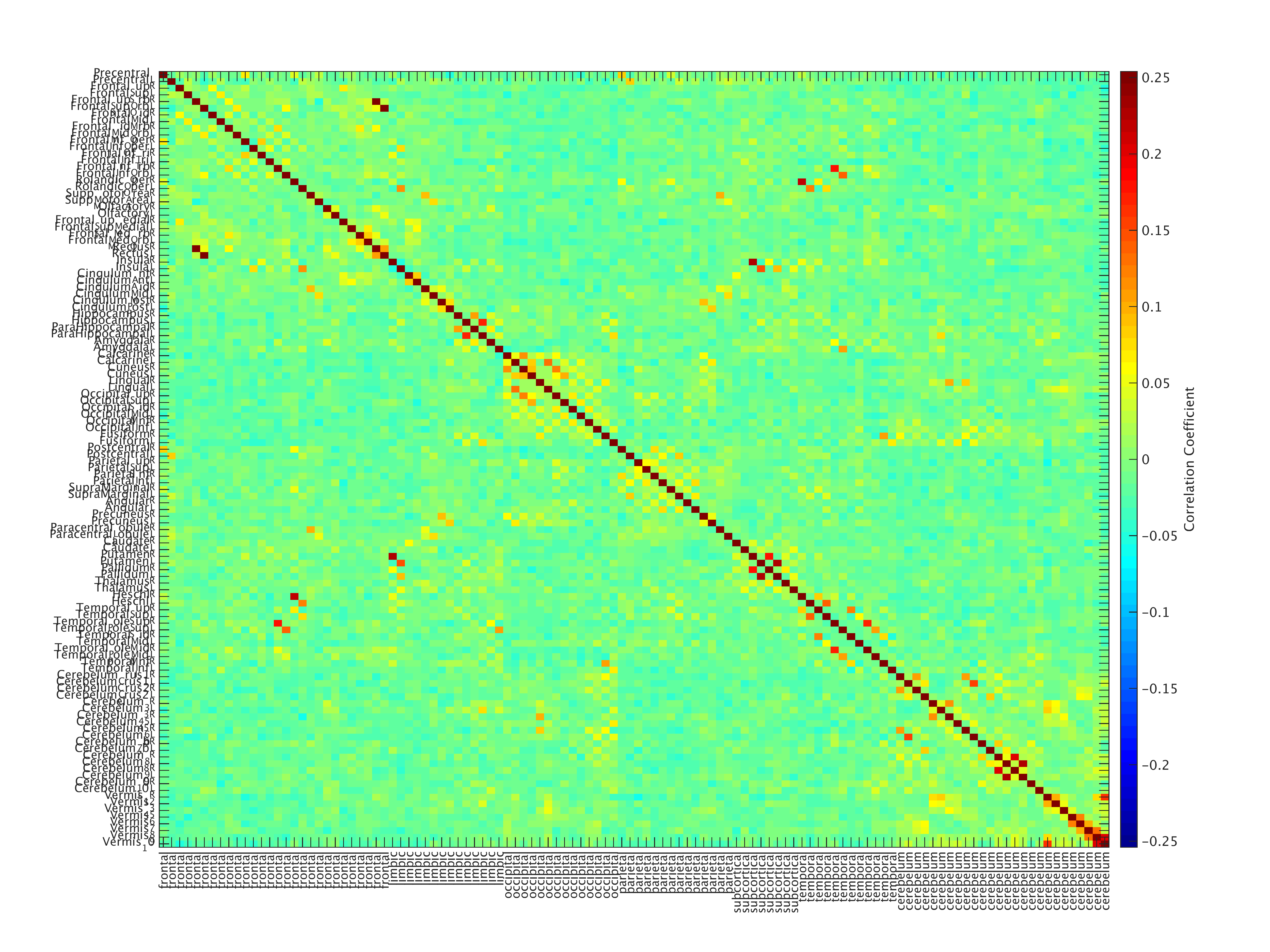

Supplement: Supplementary file 1. — This zip file contains high resolution images of the adjacency matrices for the MEG connectivity analysis suggested by the editor and reviewers. DOI: http://dx.doi.org/10.7554/eLife.23608.021 [file elife-23608-supp1.zip › hi-res_adjacency_matrices/alpha/downsampled/zscore/alpha.ave.aal.thr.zscore.r.downsampled.png]

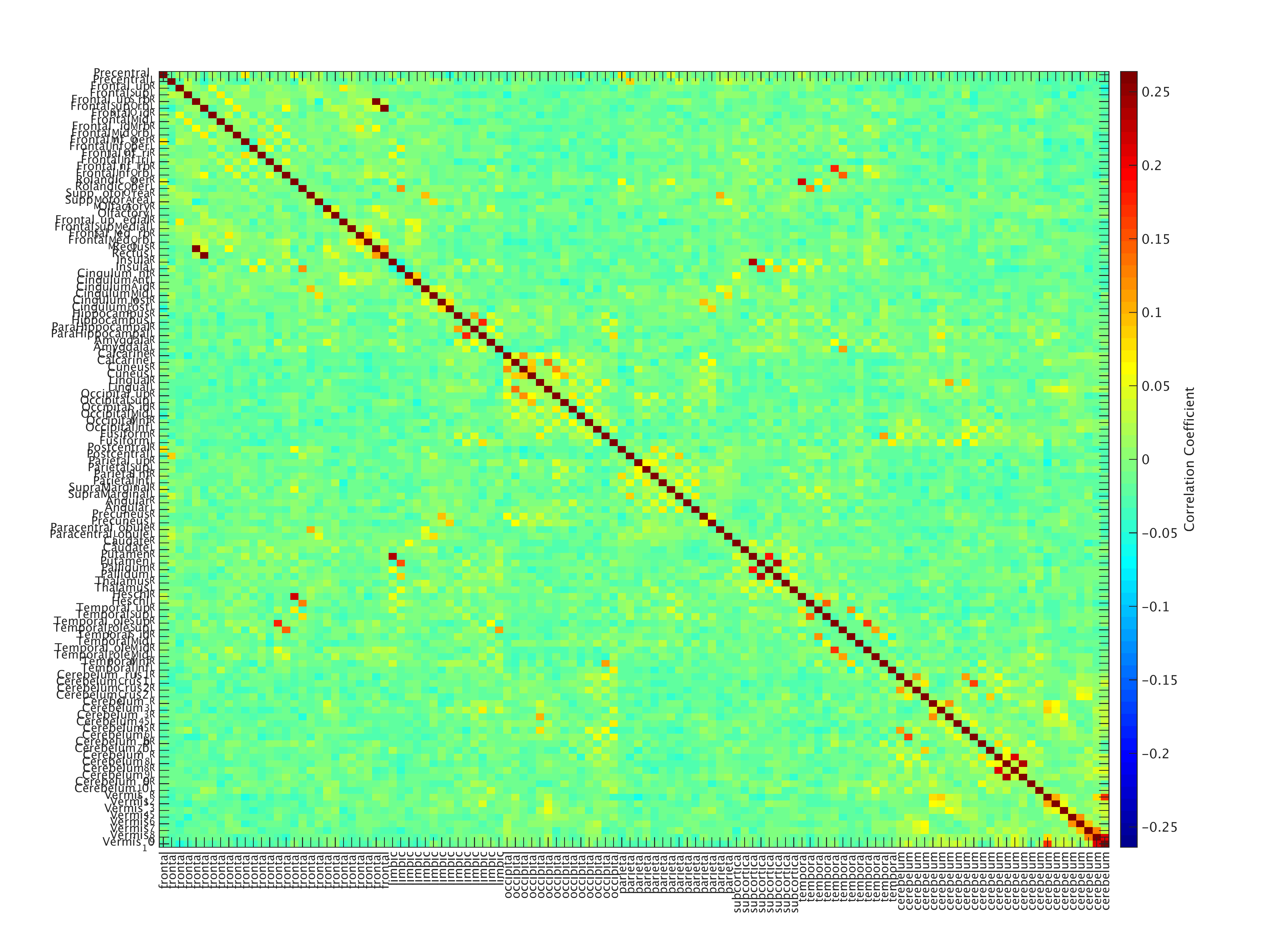

Supplement: Supplementary file 1. — This zip file contains high resolution images of the adjacency matrices for the MEG connectivity analysis suggested by the editor and reviewers. DOI: http://dx.doi.org/10.7554/eLife.23608.021 [file elife-23608-supp1.zip › hi-res_adjacency_matrices/alpha/downsampled/zscore/alpha.ave.aal.thr.zscore.z.downsampled.png]

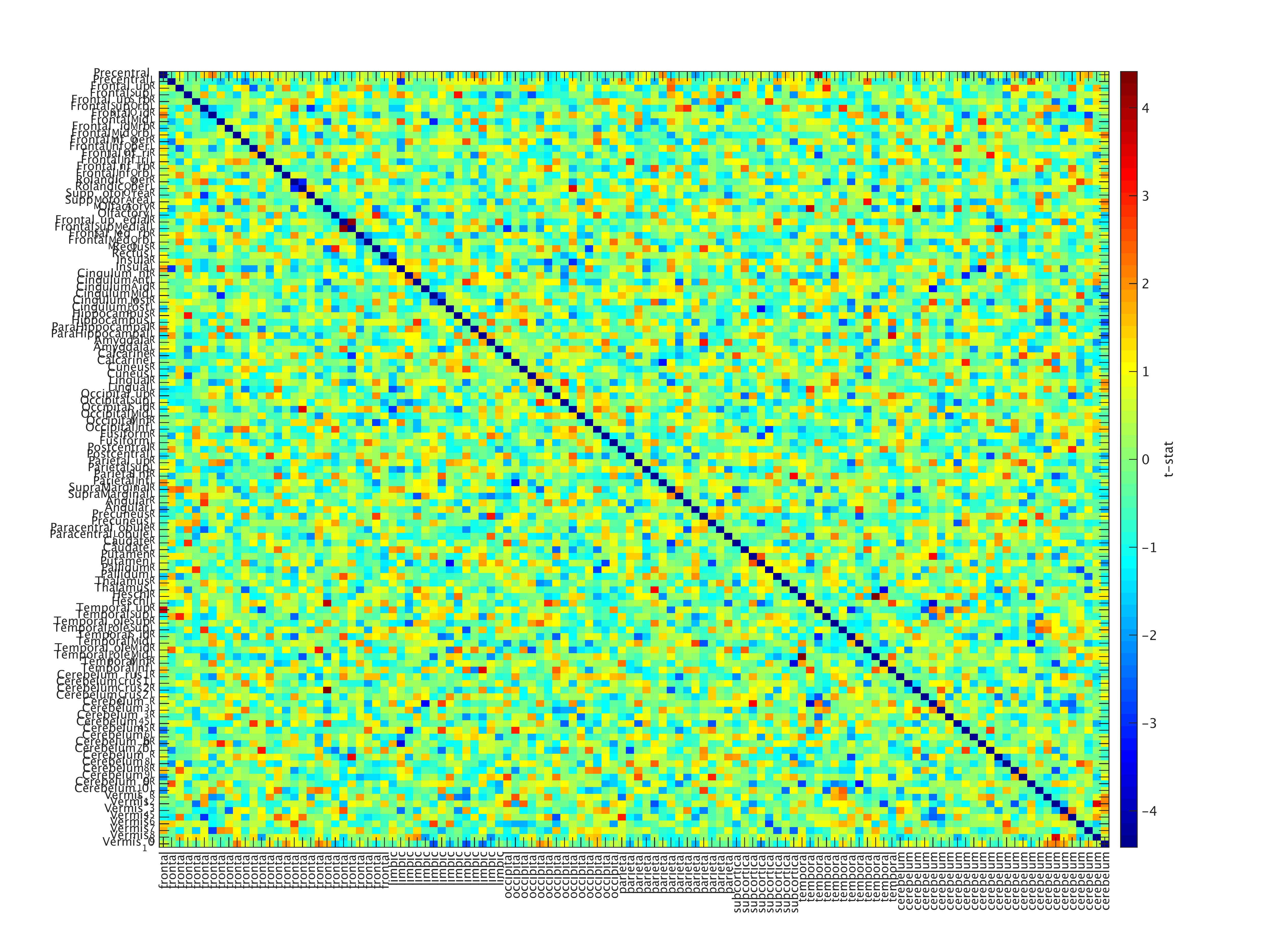

Supplement: Supplementary file 1. — This zip file contains high resolution images of the adjacency matrices for the MEG connectivity analysis suggested by the editor and reviewers. DOI: http://dx.doi.org/10.7554/eLife.23608.021 [file elife-23608-supp1.zip › hi-res_adjacency_matrices/alpha/downsampled/zscore/alpha.tstat.aal.zscore.r.downsampled.png]

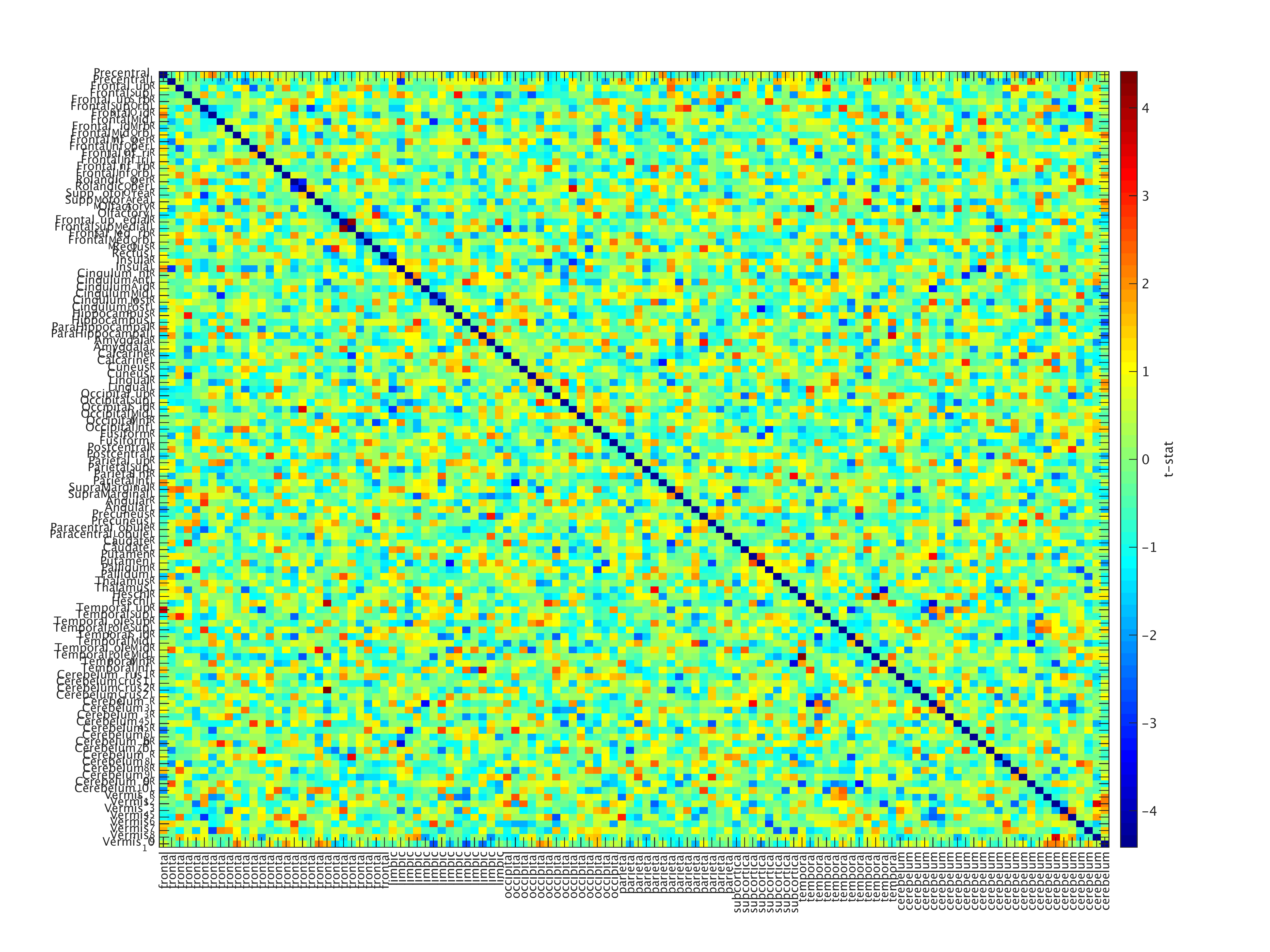

Supplement: Supplementary file 1. — This zip file contains high resolution images of the adjacency matrices for the MEG connectivity analysis suggested by the editor and reviewers. DOI: http://dx.doi.org/10.7554/eLife.23608.021 [file elife-23608-supp1.zip › hi-res_adjacency_matrices/alpha/downsampled/zscore/alpha.tstat.aal.zscore.z.downsampled.png]

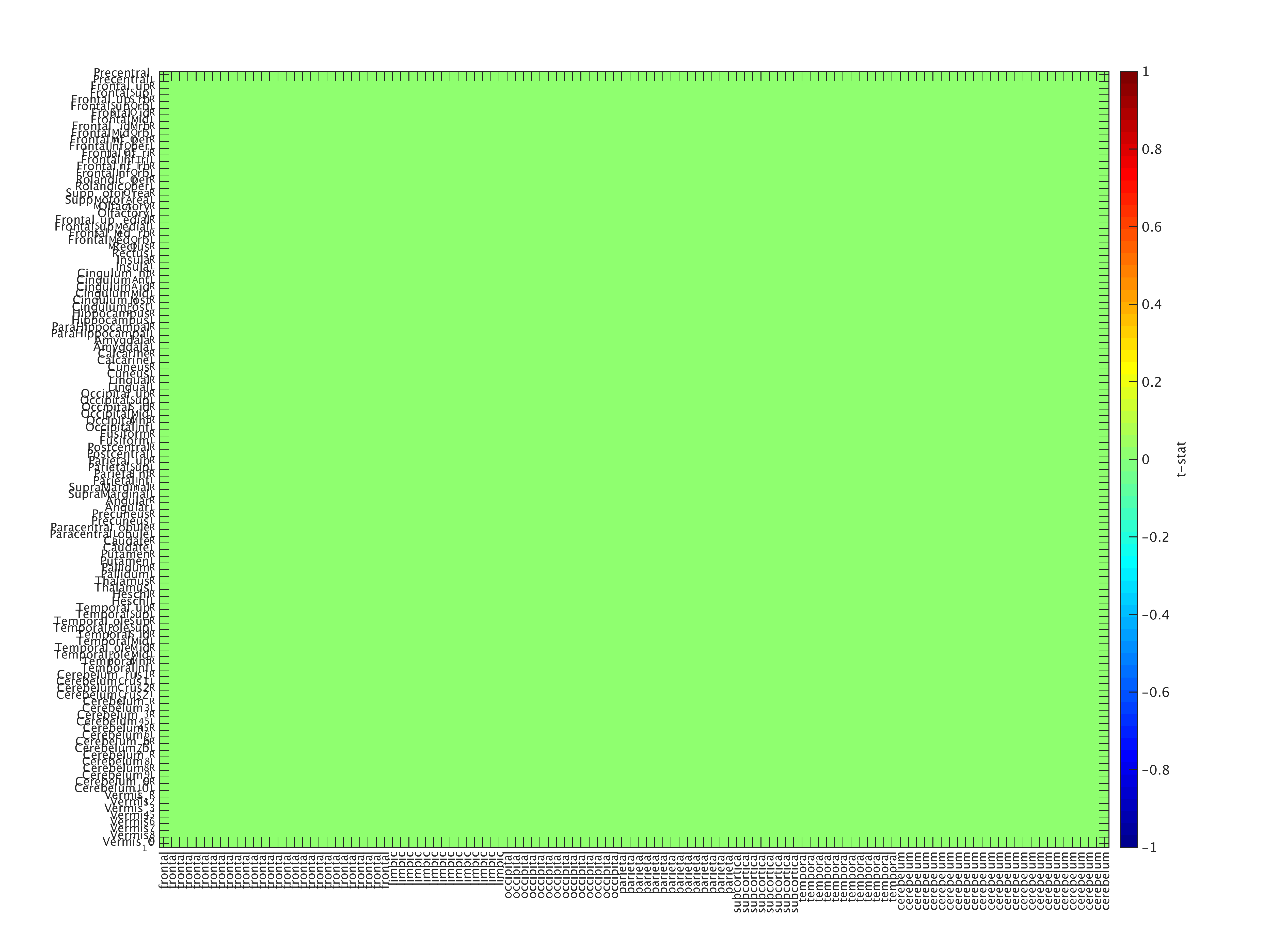

Supplement: Supplementary file 1. — This zip file contains high resolution images of the adjacency matrices for the MEG connectivity analysis suggested by the editor and reviewers. DOI: http://dx.doi.org/10.7554/eLife.23608.021 [file elife-23608-supp1.zip › hi-res_adjacency_matrices/alpha/downsampled/zscore/alpha.t-thresh.aal.zscore.r.downsampled.png]

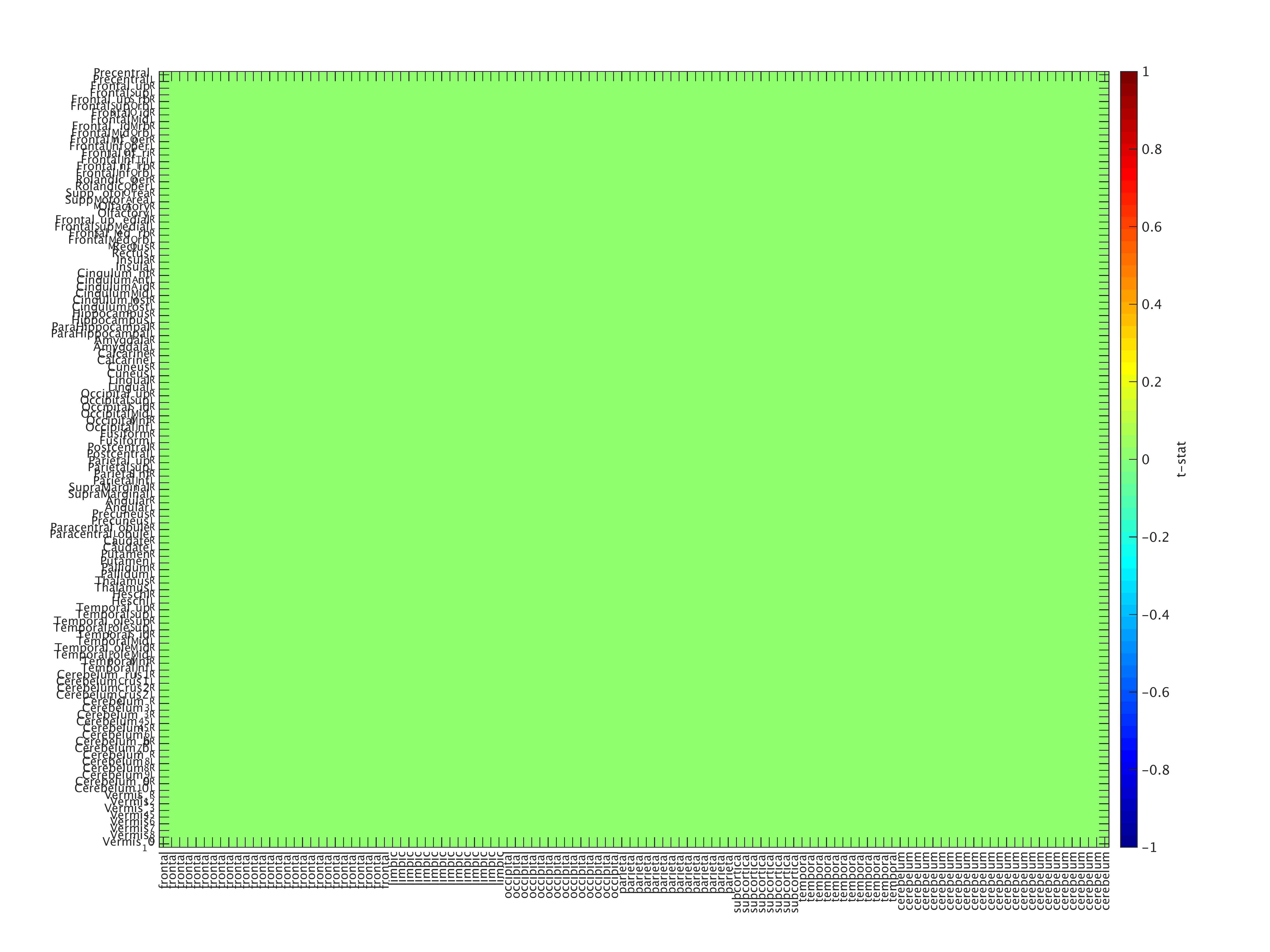

Supplement: Supplementary file 1. — This zip file contains high resolution images of the adjacency matrices for the MEG connectivity analysis suggested by the editor and reviewers. DOI: http://dx.doi.org/10.7554/eLife.23608.021 [file elife-23608-supp1.zip › hi-res_adjacency_matrices/alpha/downsampled/zscore/alpha.t-thresh.aal.zscore.z.downsampled.png]

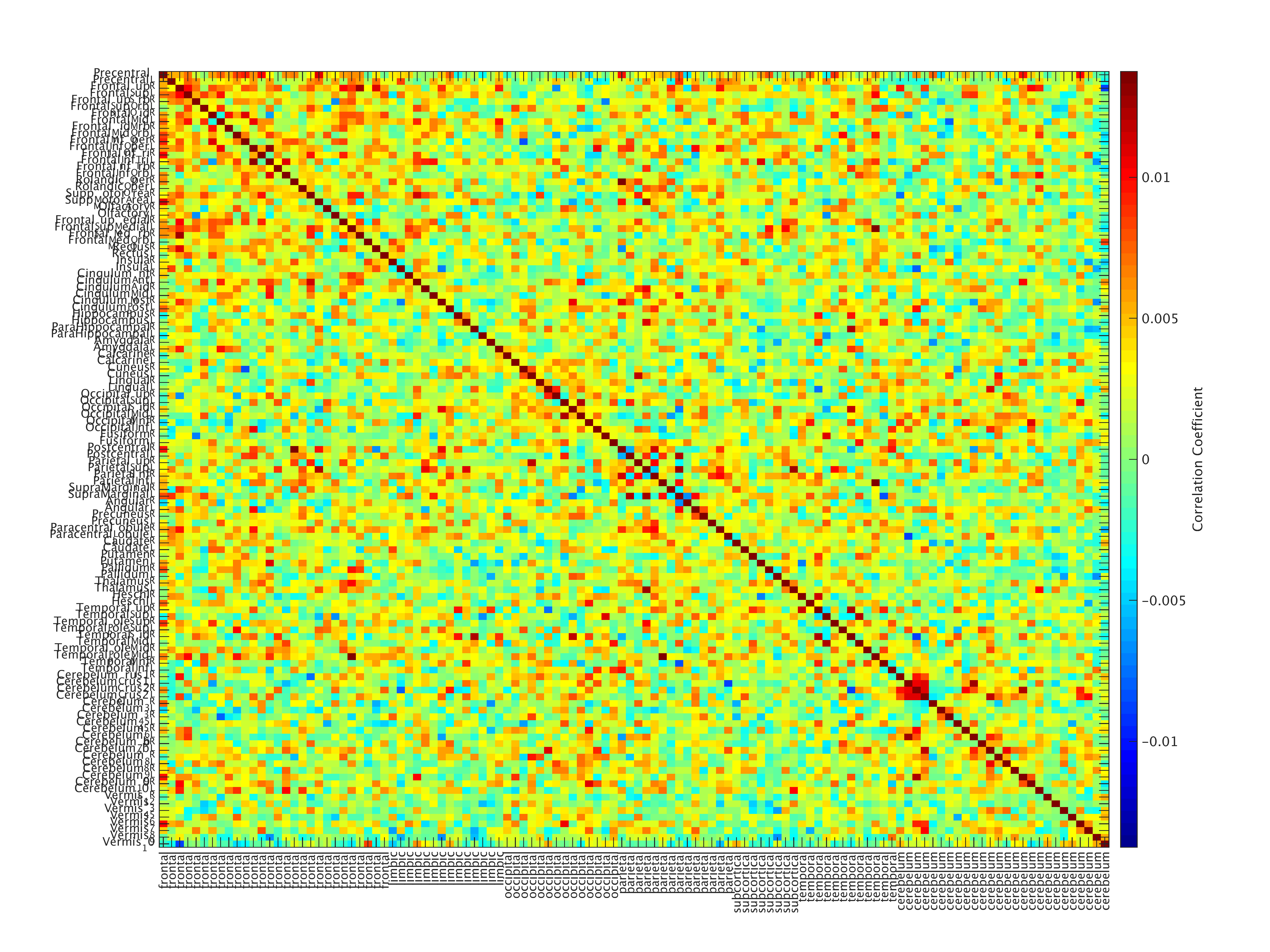

Supplement: Supplementary file 1. — This zip file contains high resolution images of the adjacency matrices for the MEG connectivity analysis suggested by the editor and reviewers. DOI: http://dx.doi.org/10.7554/eLife.23608.021 [file elife-23608-supp1.zip › hi-res_adjacency_matrices/alpha/not_downsampled/raw/alpha.ave.aal.saf.raw.r.not_downsampled.png]

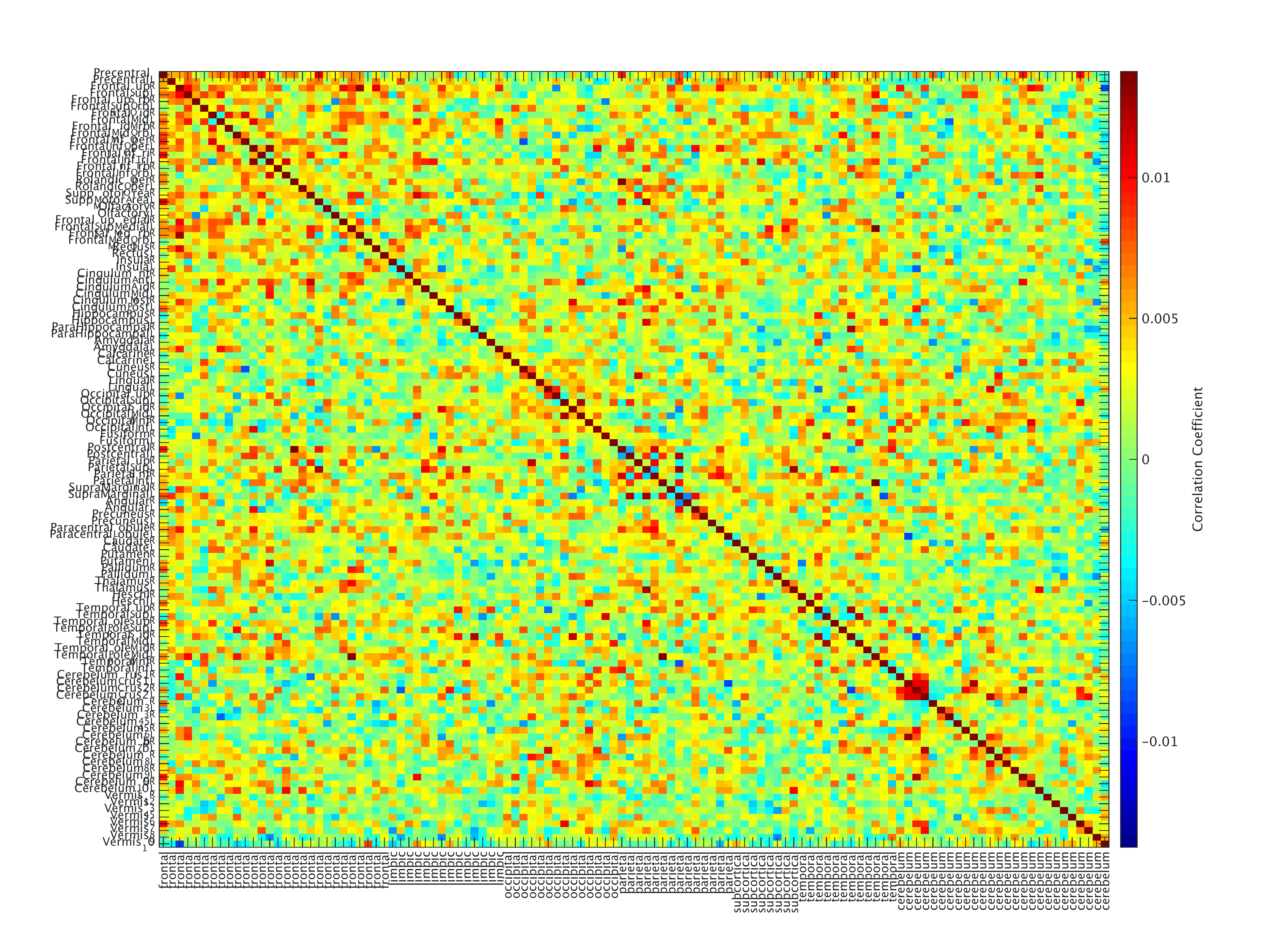

Supplement: Supplementary file 1. — This zip file contains high resolution images of the adjacency matrices for the MEG connectivity analysis suggested by the editor and reviewers. DOI: http://dx.doi.org/10.7554/eLife.23608.021 [file elife-23608-supp1.zip › hi-res_adjacency_matrices/alpha/not_downsampled/raw/alpha.ave.aal.saf.raw.z.not_downsampled.png]

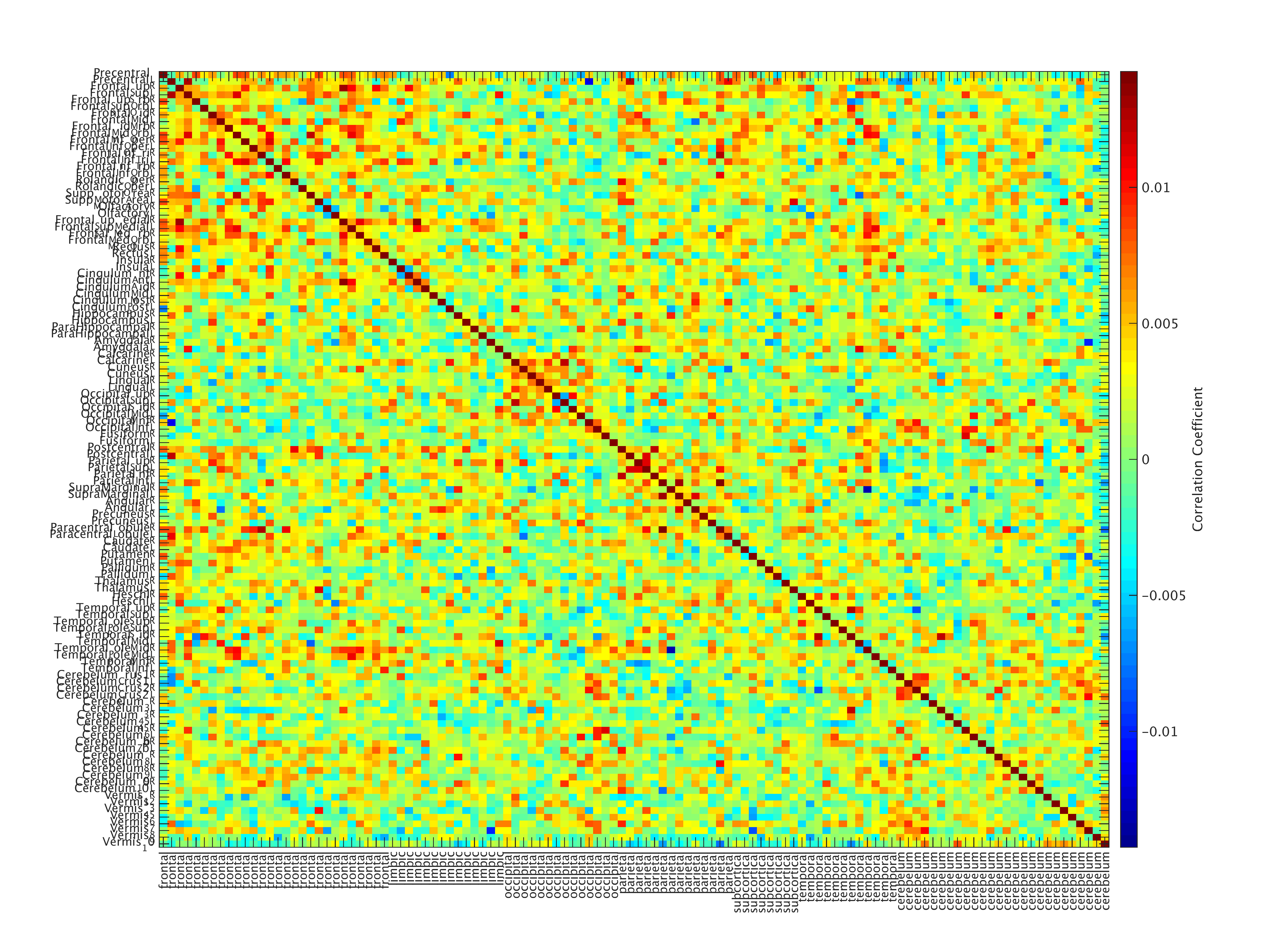

Supplement: Supplementary file 1. — This zip file contains high resolution images of the adjacency matrices for the MEG connectivity analysis suggested by the editor and reviewers. DOI: http://dx.doi.org/10.7554/eLife.23608.021 [file elife-23608-supp1.zip › hi-res_adjacency_matrices/alpha/not_downsampled/raw/alpha.ave.aal.thr.raw.r.not_downsampled.png]

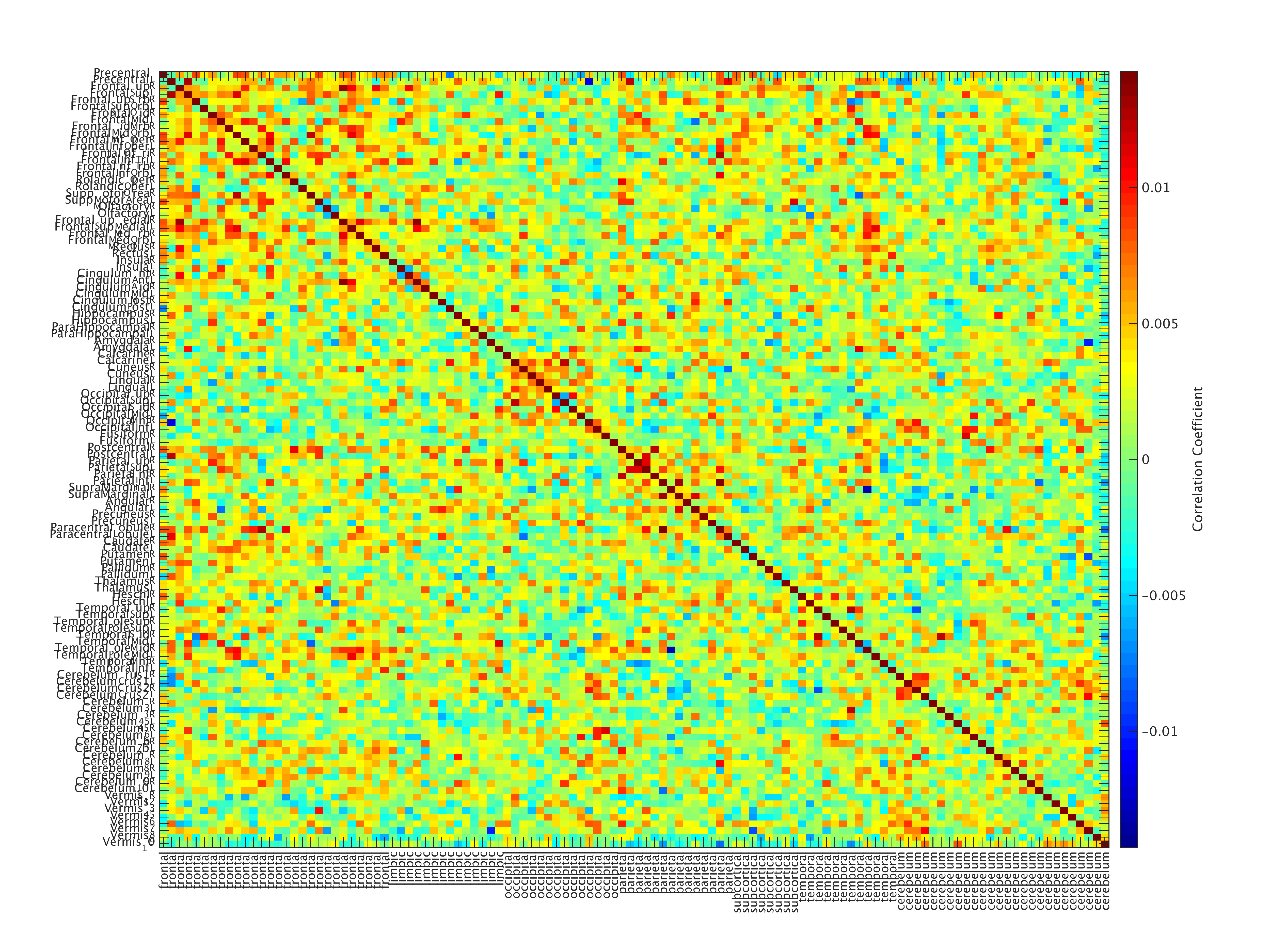

Supplement: Supplementary file 1. — This zip file contains high resolution images of the adjacency matrices for the MEG connectivity analysis suggested by the editor and reviewers. DOI: http://dx.doi.org/10.7554/eLife.23608.021 [file elife-23608-supp1.zip › hi-res_adjacency_matrices/alpha/not_downsampled/raw/alpha.ave.aal.thr.raw.z.not_downsampled.png]

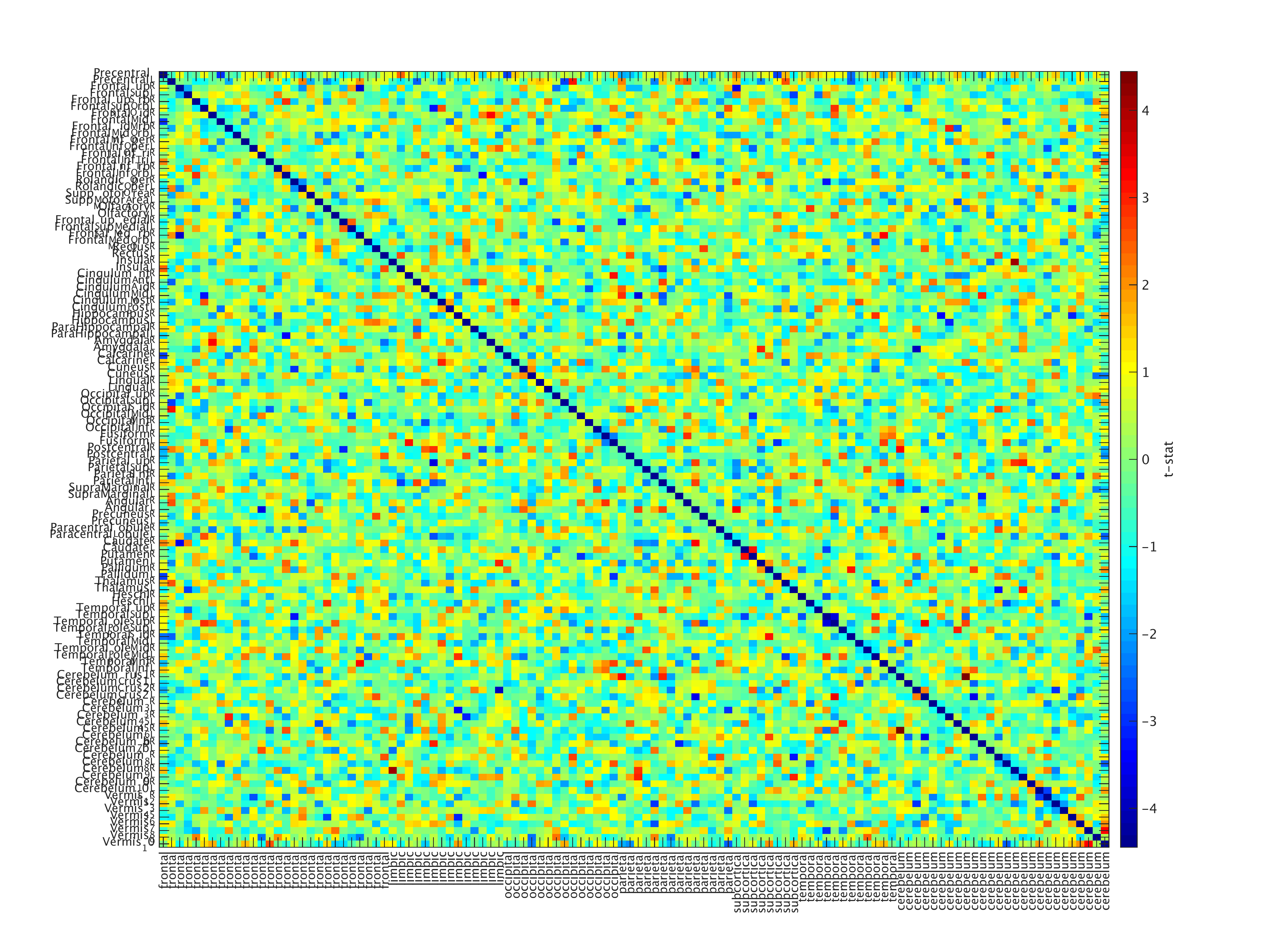

Supplement: Supplementary file 1. — This zip file contains high resolution images of the adjacency matrices for the MEG connectivity analysis suggested by the editor and reviewers. DOI: http://dx.doi.org/10.7554/eLife.23608.021 [file elife-23608-supp1.zip › hi-res_adjacency_matrices/alpha/not_downsampled/raw/alpha.tstat.aal.raw.r.not_downsampled.png]

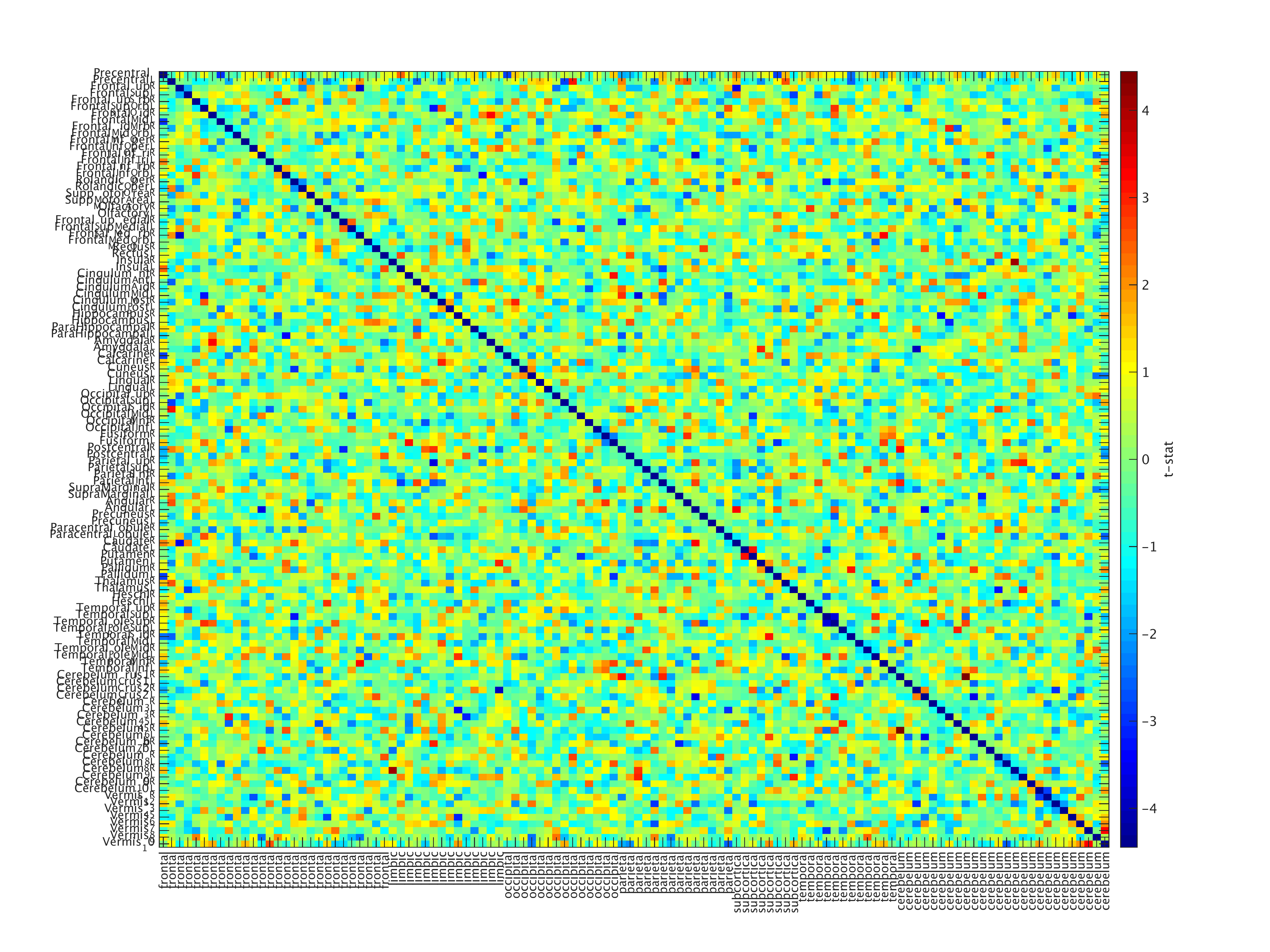

Supplement: Supplementary file 1. — This zip file contains high resolution images of the adjacency matrices for the MEG connectivity analysis suggested by the editor and reviewers. DOI: http://dx.doi.org/10.7554/eLife.23608.021 [file elife-23608-supp1.zip › hi-res_adjacency_matrices/alpha/not_downsampled/raw/alpha.tstat.aal.raw.z.not_downsampled.png]

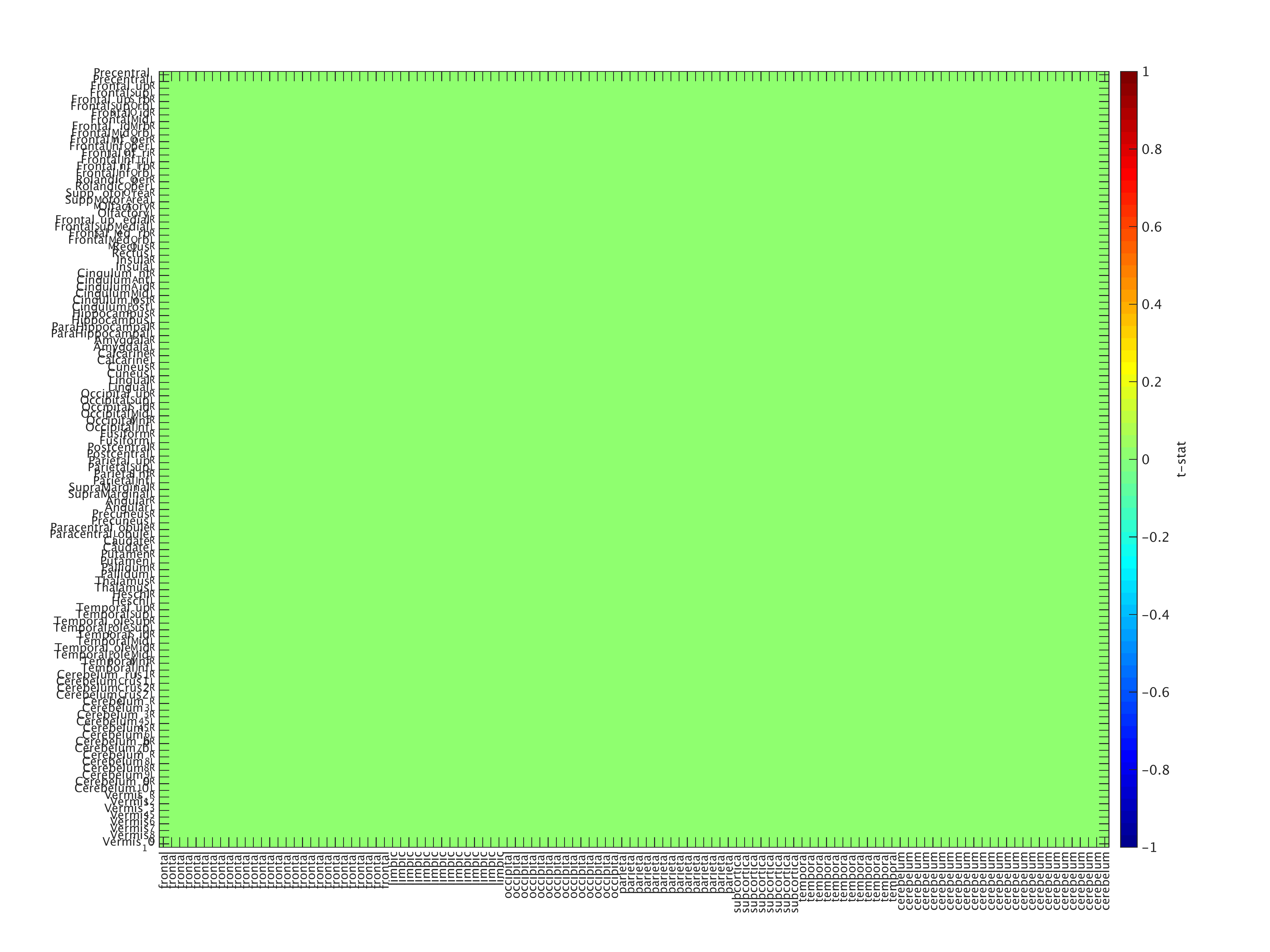

Supplement: Supplementary file 1. — This zip file contains high resolution images of the adjacency matrices for the MEG connectivity analysis suggested by the editor and reviewers. DOI: http://dx.doi.org/10.7554/eLife.23608.021 [file elife-23608-supp1.zip › hi-res_adjacency_matrices/alpha/not_downsampled/raw/alpha.t-thresh.aal.raw.r.not_downsampled.png]

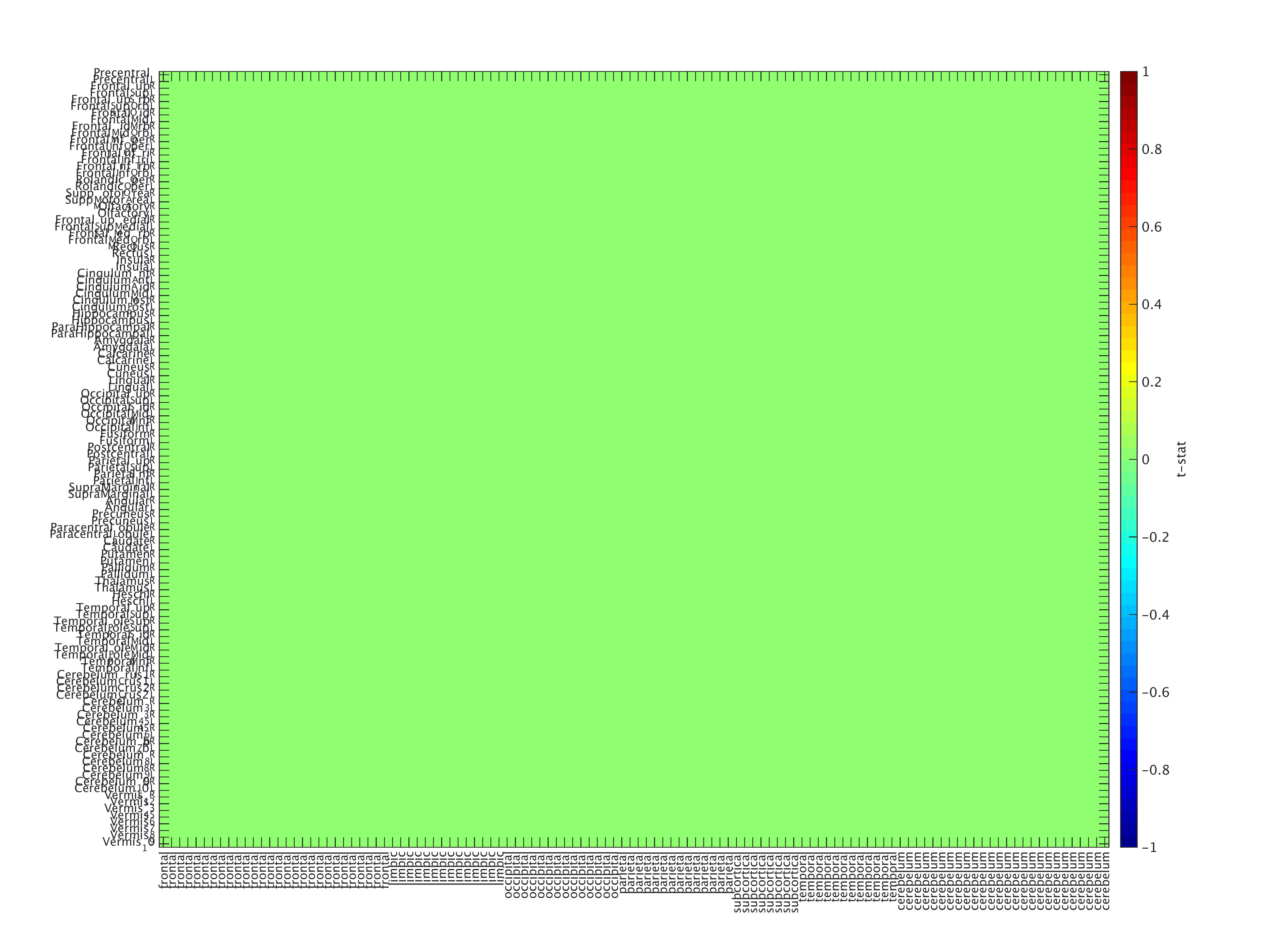

Supplement: Supplementary file 1. — This zip file contains high resolution images of the adjacency matrices for the MEG connectivity analysis suggested by the editor and reviewers. DOI: http://dx.doi.org/10.7554/eLife.23608.021 [file elife-23608-supp1.zip › hi-res_adjacency_matrices/alpha/not_downsampled/raw/alpha.t-thresh.aal.raw.z.not_downsampled.png]

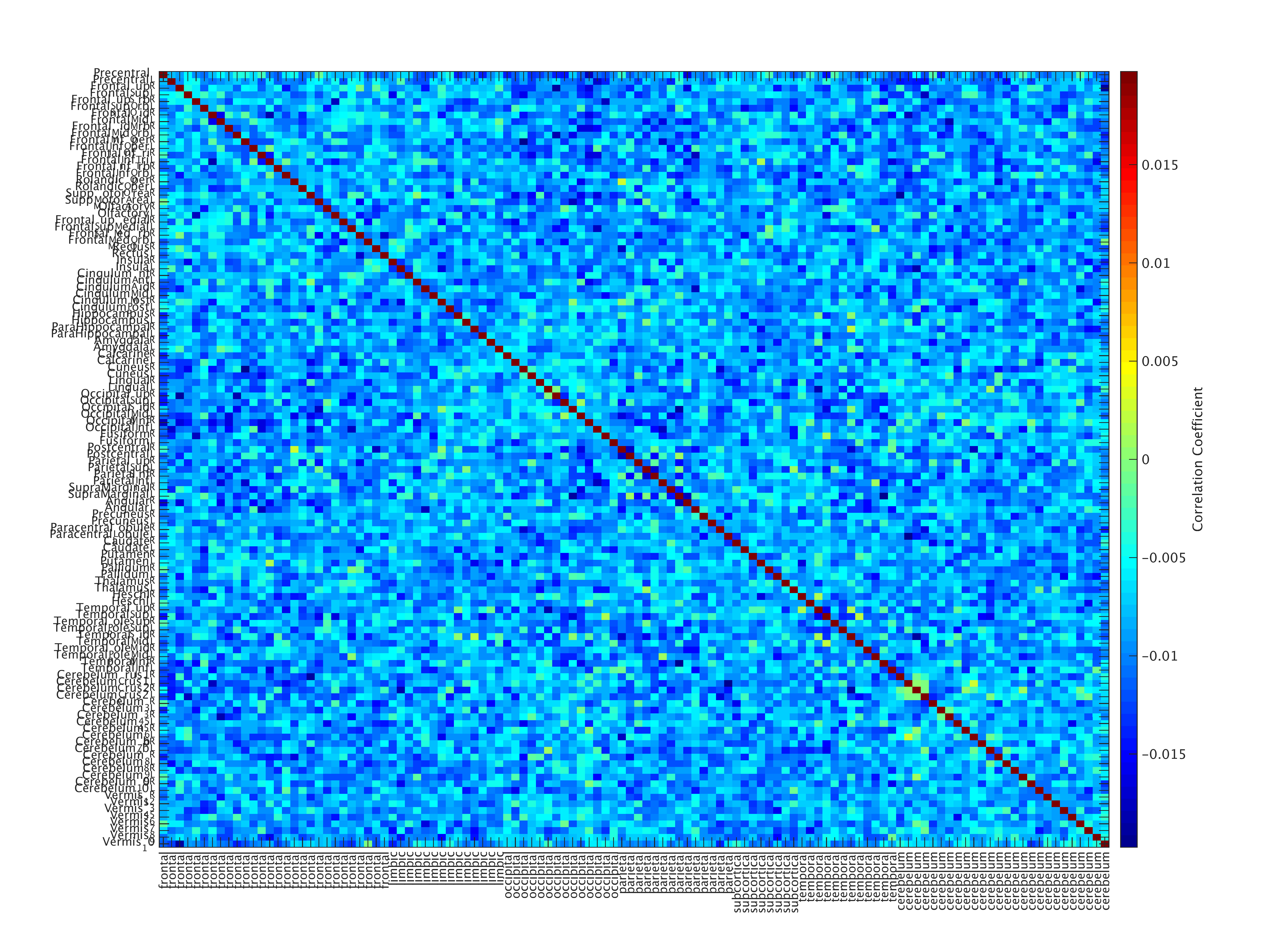

Supplement: Supplementary file 1. — This zip file contains high resolution images of the adjacency matrices for the MEG connectivity analysis suggested by the editor and reviewers. DOI: http://dx.doi.org/10.7554/eLife.23608.021 [file elife-23608-supp1.zip › hi-res_adjacency_matrices/alpha/not_downsampled/zscore/alpha.ave.aal.saf.zscore.r.not_downsampled.png]

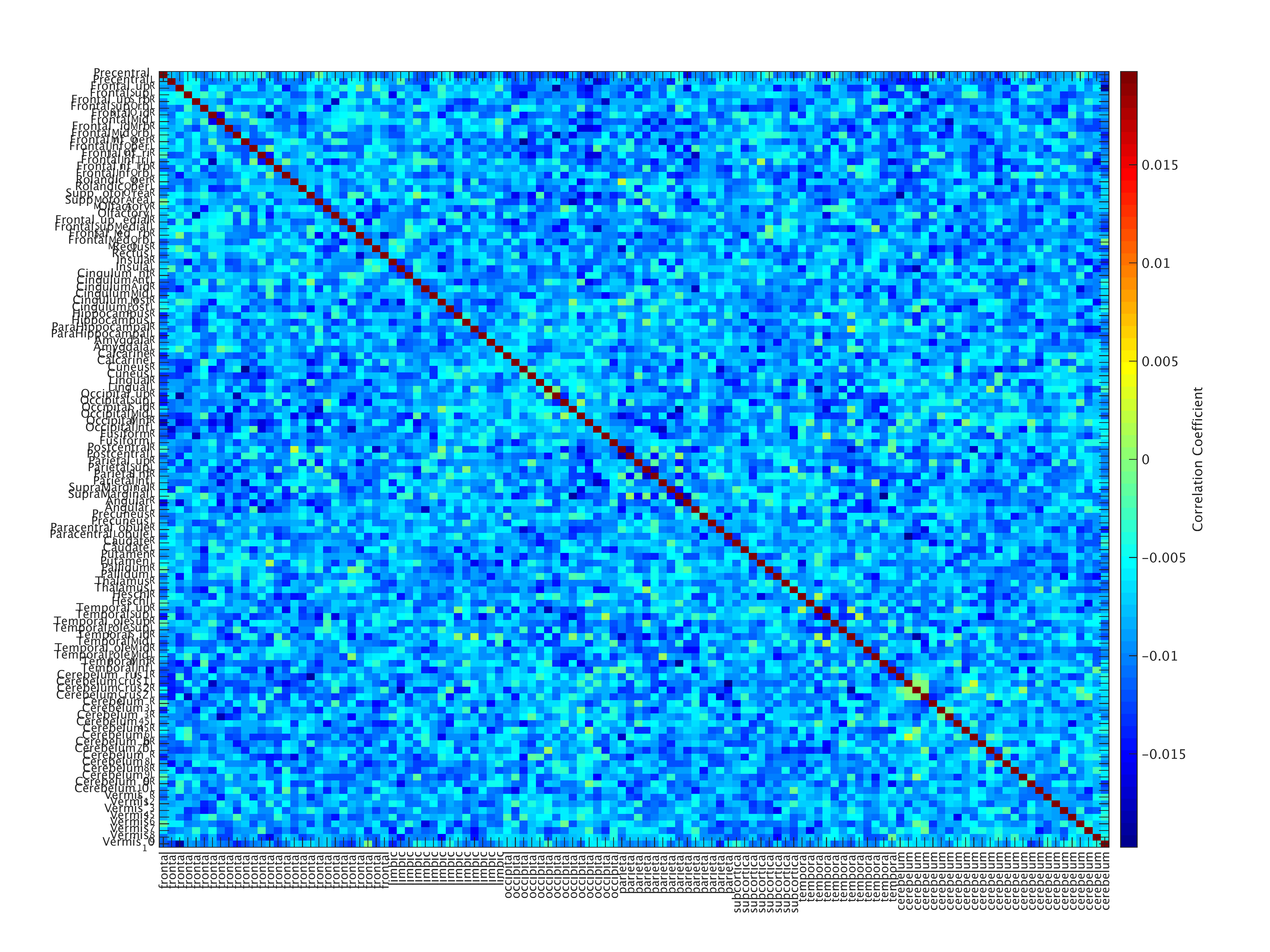

Supplement: Supplementary file 1. — This zip file contains high resolution images of the adjacency matrices for the MEG connectivity analysis suggested by the editor and reviewers. DOI: http://dx.doi.org/10.7554/eLife.23608.021 [file elife-23608-supp1.zip › hi-res_adjacency_matrices/alpha/not_downsampled/zscore/alpha.ave.aal.saf.zscore.z.not_downsampled.png]

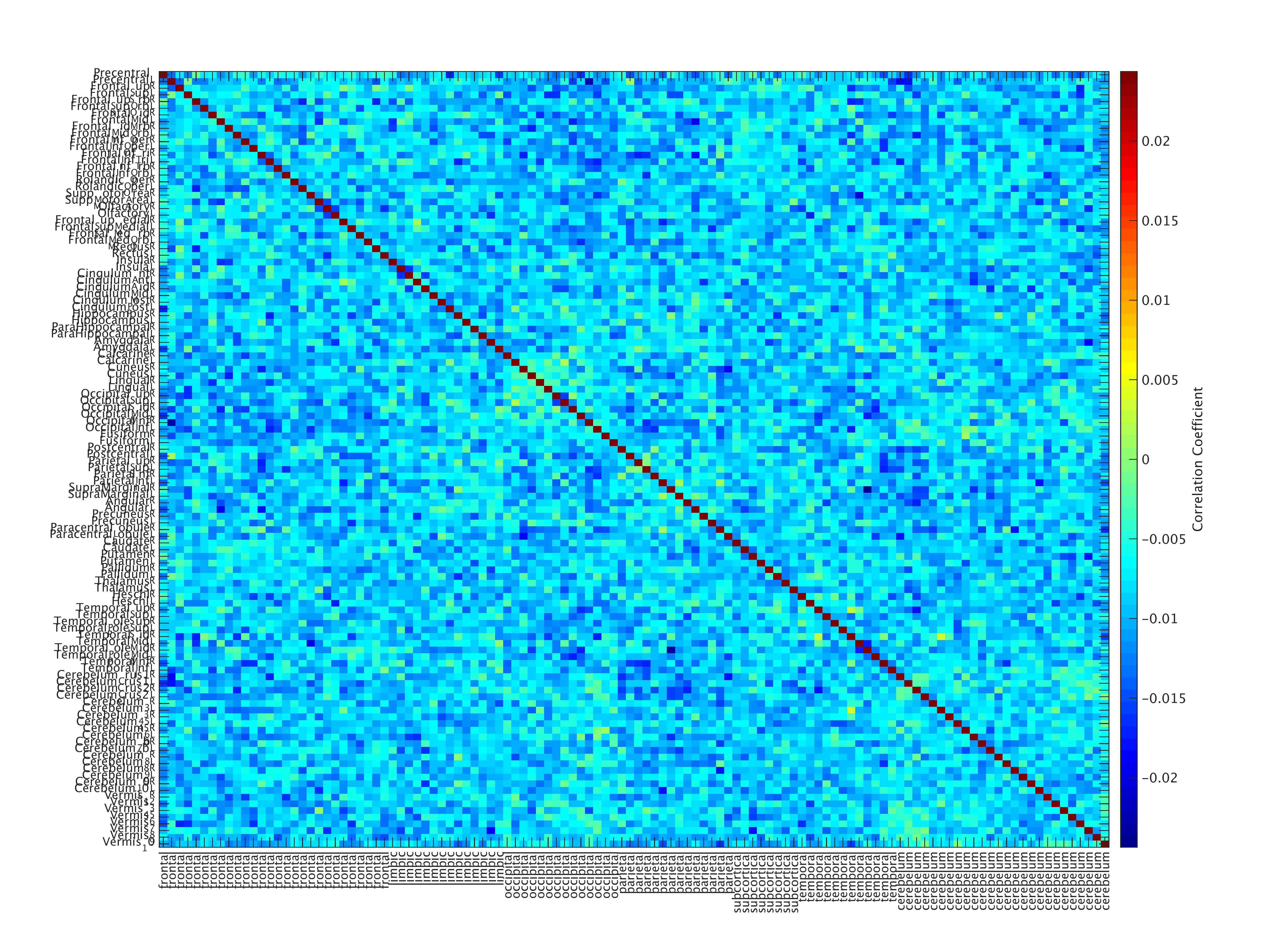

Supplement: Supplementary file 1. — This zip file contains high resolution images of the adjacency matrices for the MEG connectivity analysis suggested by the editor and reviewers. DOI: http://dx.doi.org/10.7554/eLife.23608.021 [file elife-23608-supp1.zip › hi-res_adjacency_matrices/alpha/not_downsampled/zscore/alpha.ave.aal.thr.zscore.r.not_downsampled.png]

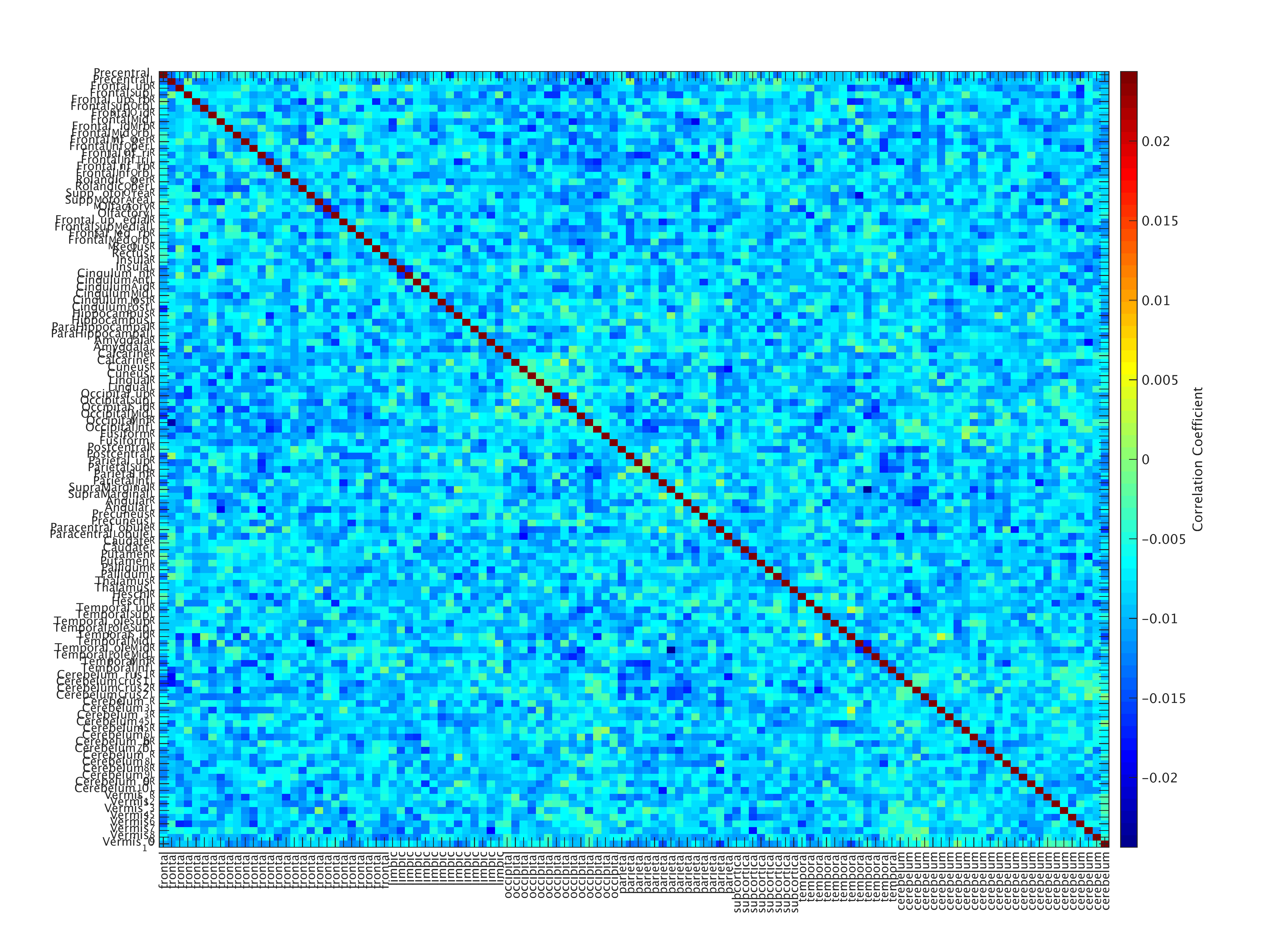

Supplement: Supplementary file 1. — This zip file contains high resolution images of the adjacency matrices for the MEG connectivity analysis suggested by the editor and reviewers. DOI: http://dx.doi.org/10.7554/eLife.23608.021 [file elife-23608-supp1.zip › hi-res_adjacency_matrices/alpha/not_downsampled/zscore/alpha.ave.aal.thr.zscore.z.not_downsampled.png]

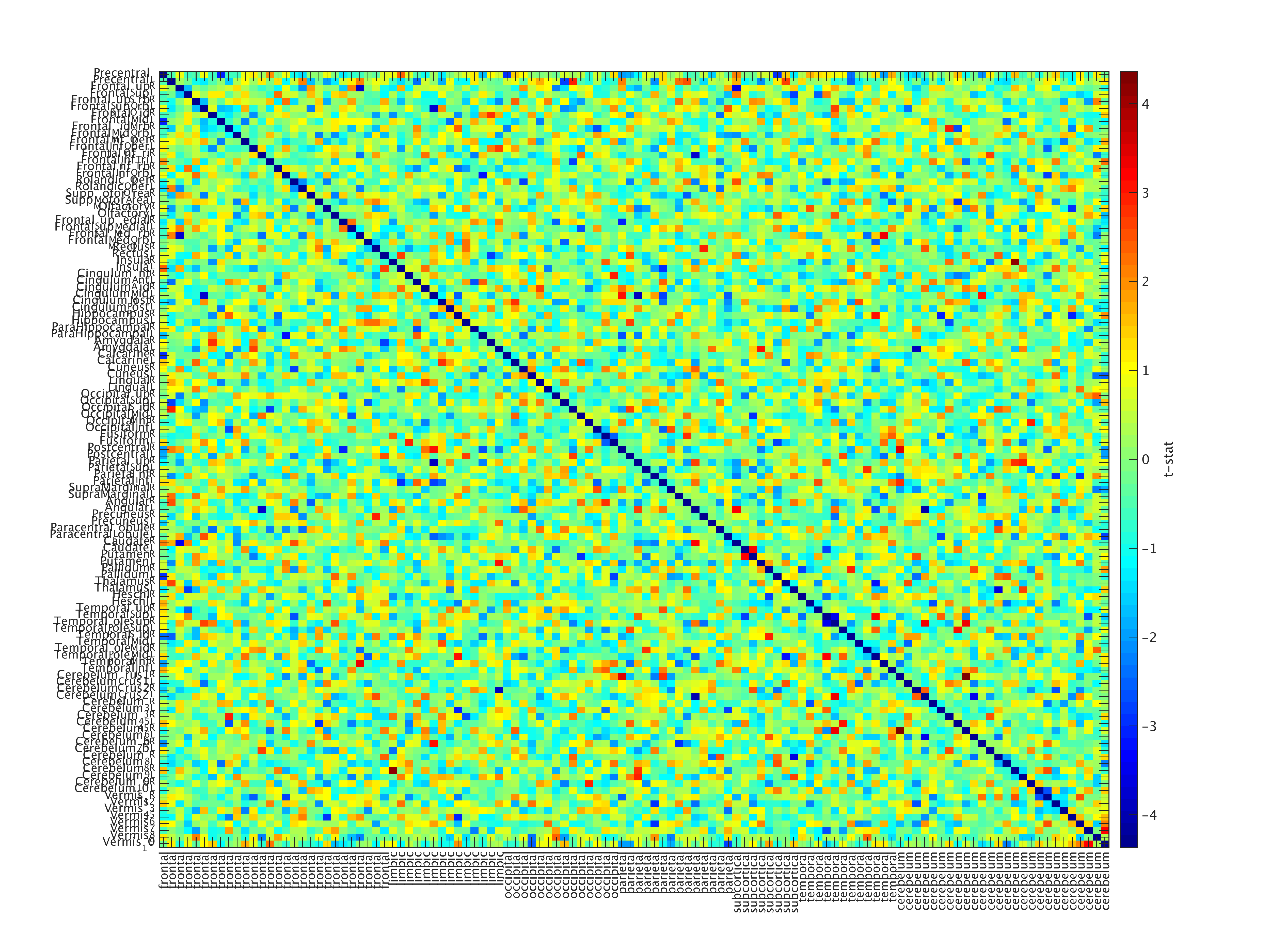

Supplement: Supplementary file 1. — This zip file contains high resolution images of the adjacency matrices for the MEG connectivity analysis suggested by the editor and reviewers. DOI: http://dx.doi.org/10.7554/eLife.23608.021 [file elife-23608-supp1.zip › hi-res_adjacency_matrices/alpha/not_downsampled/zscore/alpha.tstat.aal.zscore.r.not_downsampled.png]

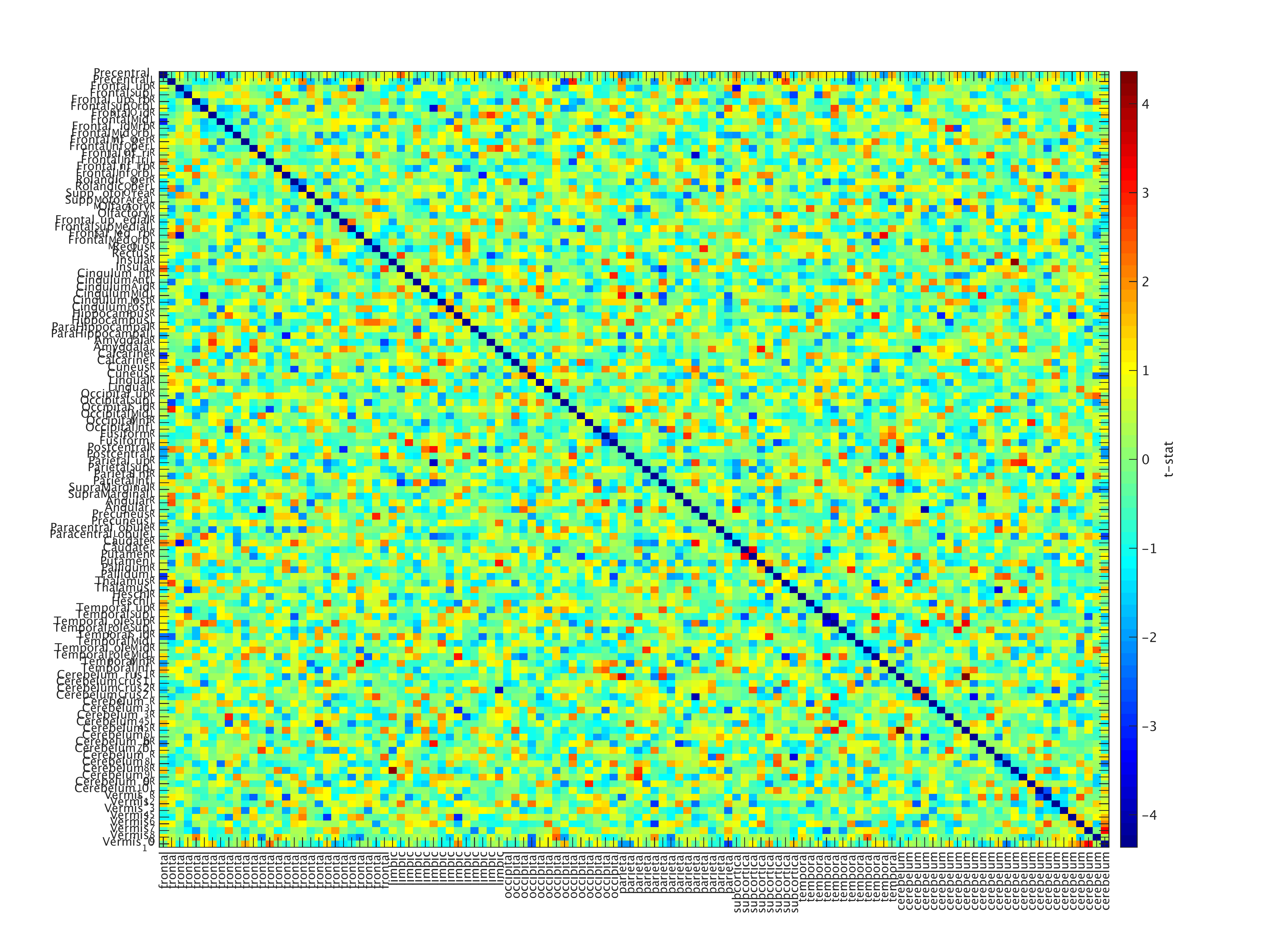

Supplement: Supplementary file 1. — This zip file contains high resolution images of the adjacency matrices for the MEG connectivity analysis suggested by the editor and reviewers. DOI: http://dx.doi.org/10.7554/eLife.23608.021 [file elife-23608-supp1.zip › hi-res_adjacency_matrices/alpha/not_downsampled/zscore/alpha.tstat.aal.zscore.z.not_downsampled.png]

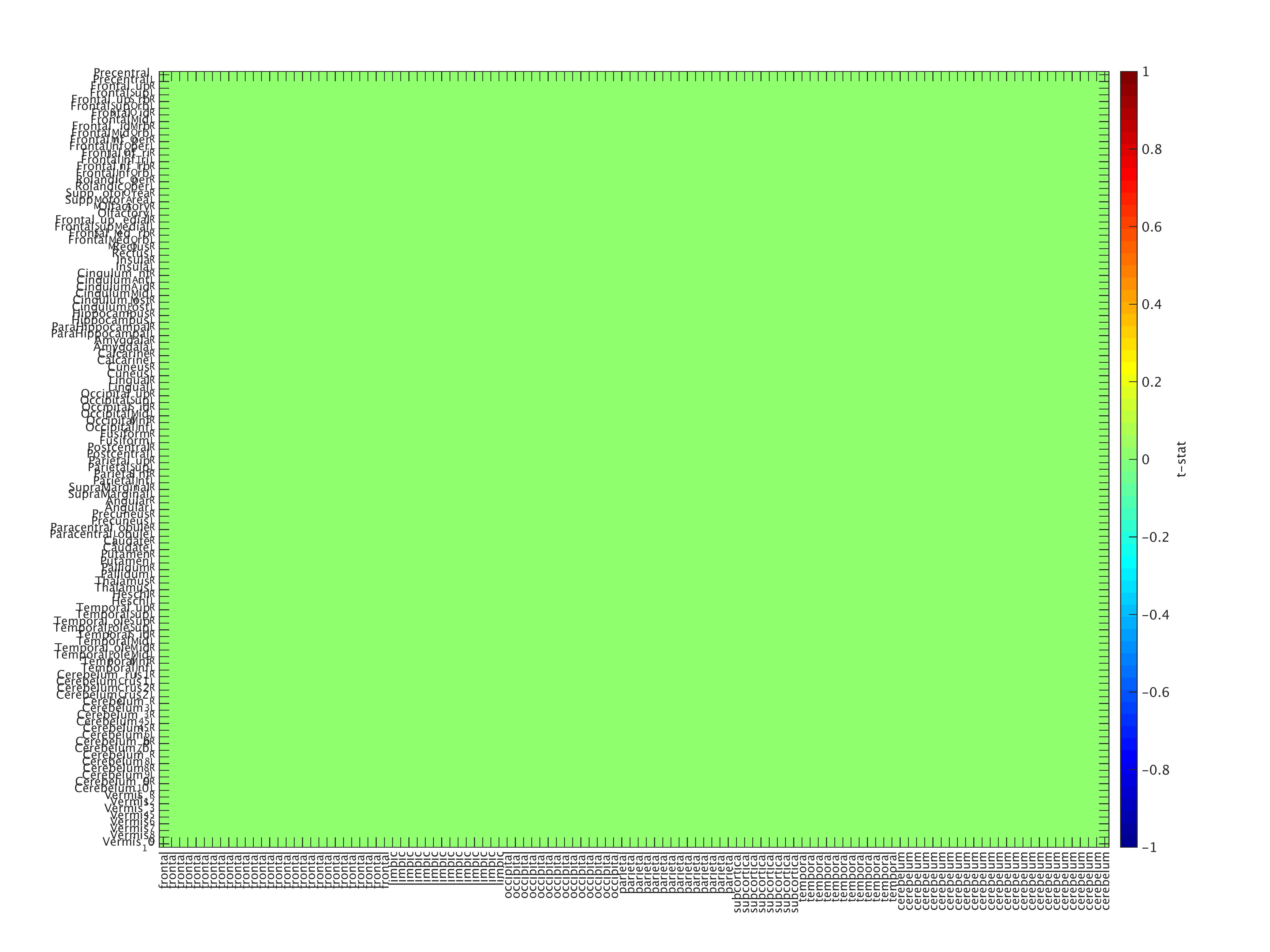

Supplement: Supplementary file 1. — This zip file contains high resolution images of the adjacency matrices for the MEG connectivity analysis suggested by the editor and reviewers. DOI: http://dx.doi.org/10.7554/eLife.23608.021 [file elife-23608-supp1.zip › hi-res_adjacency_matrices/alpha/not_downsampled/zscore/alpha.t-thresh.aal.zscore.r.not_downsampled.png]

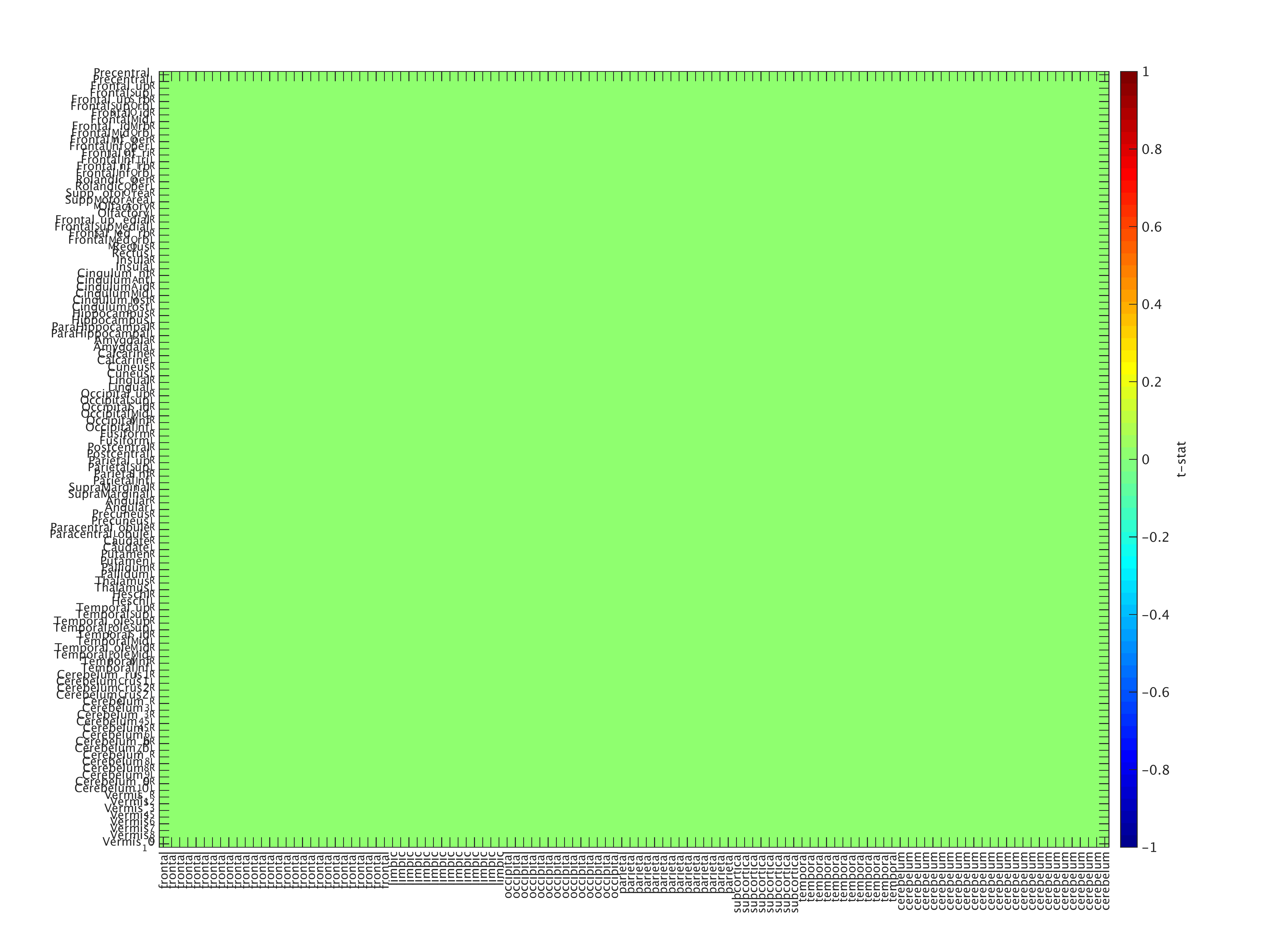

Supplement: Supplementary file 1. — This zip file contains high resolution images of the adjacency matrices for the MEG connectivity analysis suggested by the editor and reviewers. DOI: http://dx.doi.org/10.7554/eLife.23608.021 [file elife-23608-supp1.zip › hi-res_adjacency_matrices/alpha/not_downsampled/zscore/alpha.t-thresh.aal.zscore.z.not_downsampled.png]

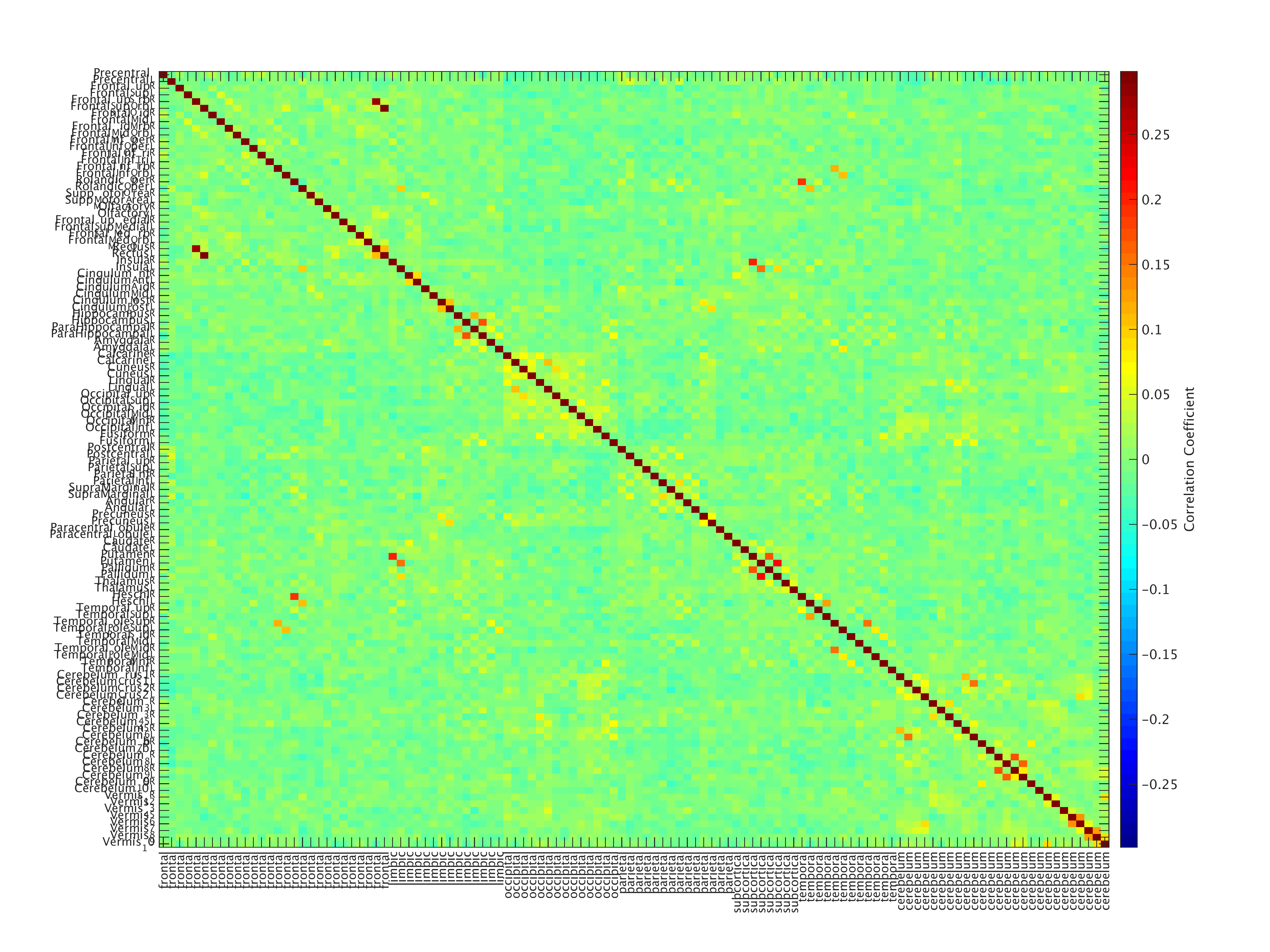

Supplement: Supplementary file 1. — This zip file contains high resolution images of the adjacency matrices for the MEG connectivity analysis suggested by the editor and reviewers. DOI: http://dx.doi.org/10.7554/eLife.23608.021 [file elife-23608-supp1.zip › hi-res_adjacency_matrices/beta/downsampled/raw/beta.ave.aal.saf.raw.r.downsampled.png]

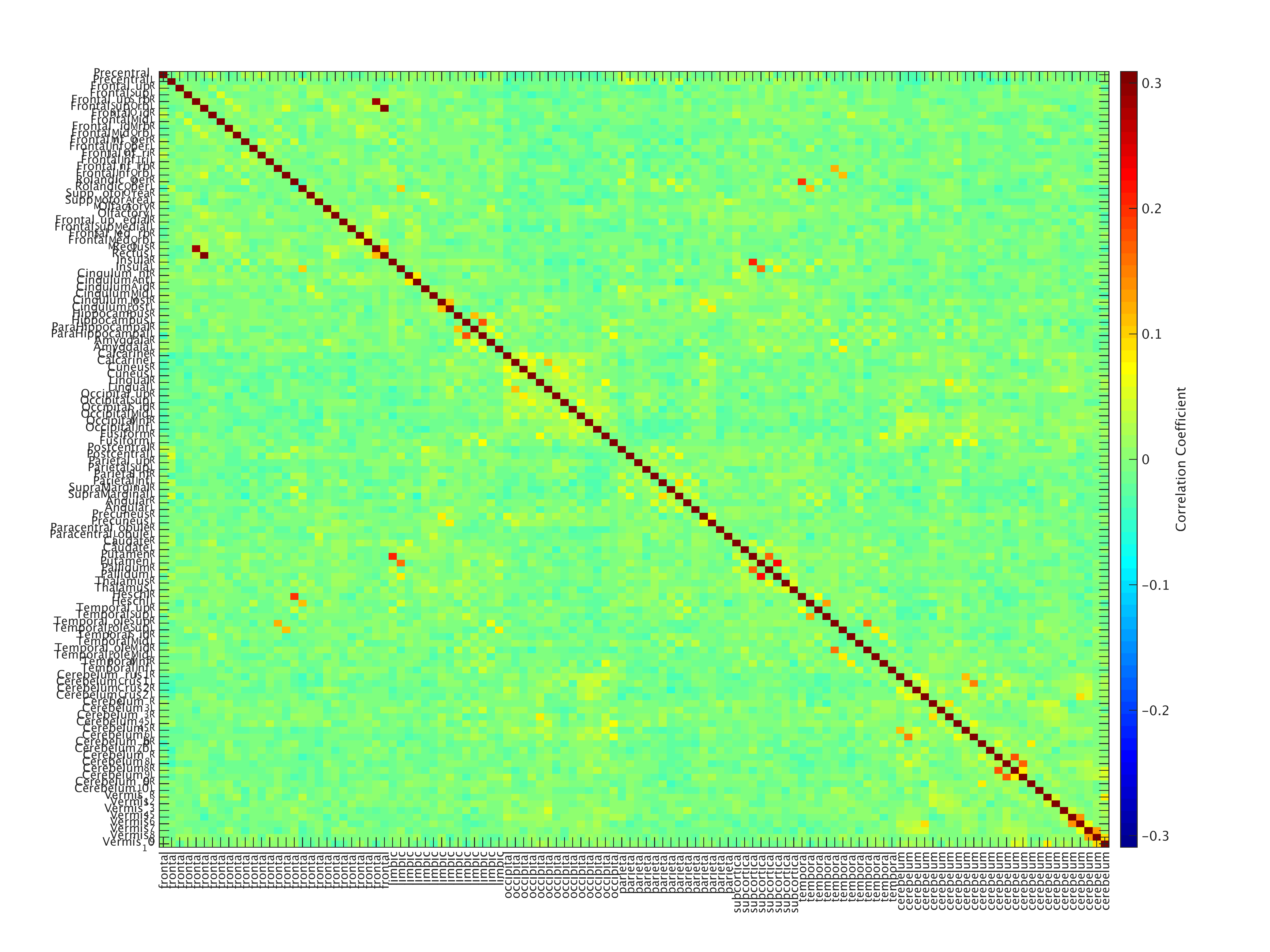

Supplement: Supplementary file 1. — This zip file contains high resolution images of the adjacency matrices for the MEG connectivity analysis suggested by the editor and reviewers. DOI: http://dx.doi.org/10.7554/eLife.23608.021 [file elife-23608-supp1.zip › hi-res_adjacency_matrices/beta/downsampled/raw/beta.ave.aal.saf.raw.z.downsampled.png]

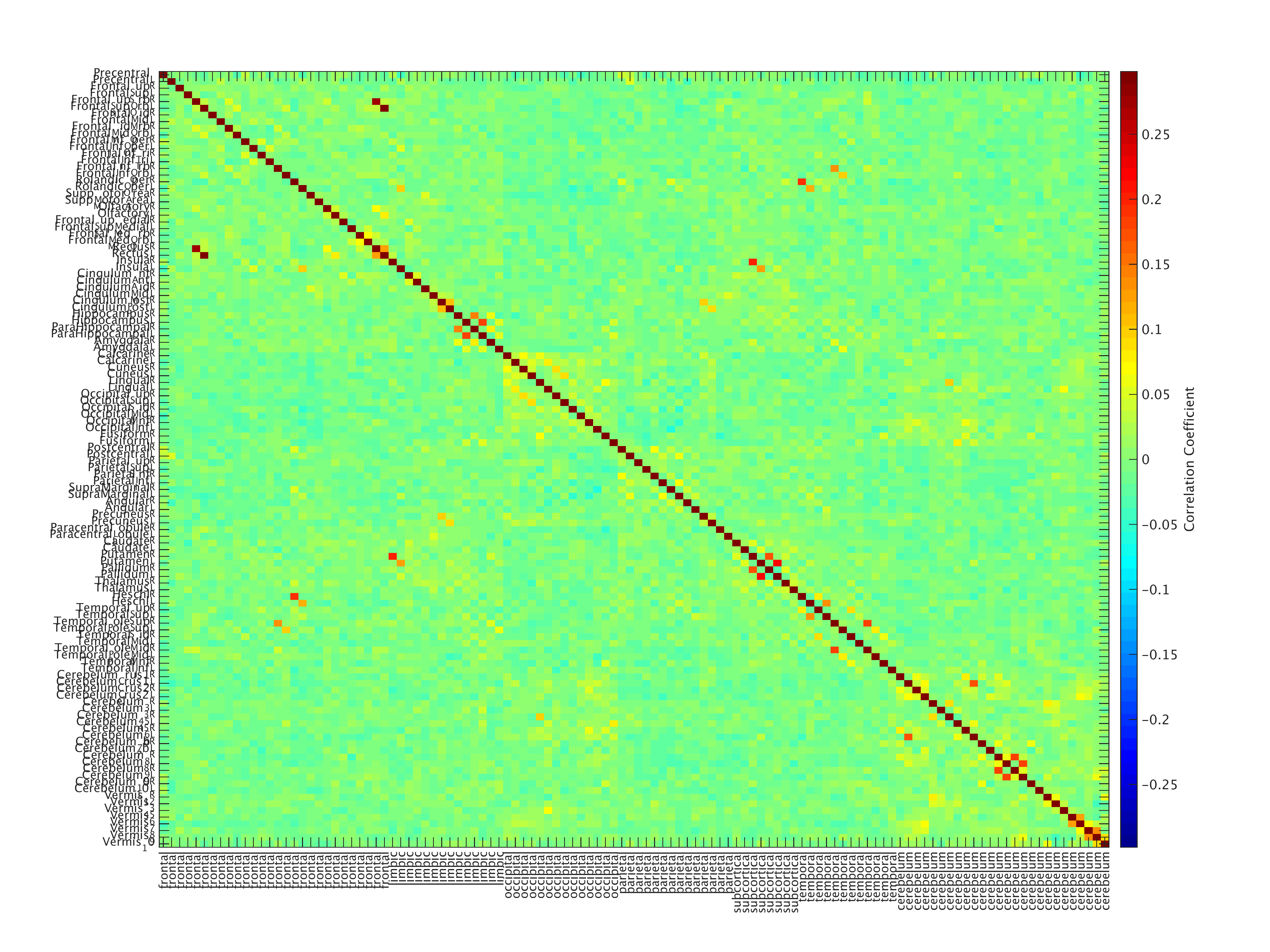

Supplement: Supplementary file 1. — This zip file contains high resolution images of the adjacency matrices for the MEG connectivity analysis suggested by the editor and reviewers. DOI: http://dx.doi.org/10.7554/eLife.23608.021 [file elife-23608-supp1.zip › hi-res_adjacency_matrices/beta/downsampled/raw/beta.ave.aal.thr.raw.r.downsampled.png]

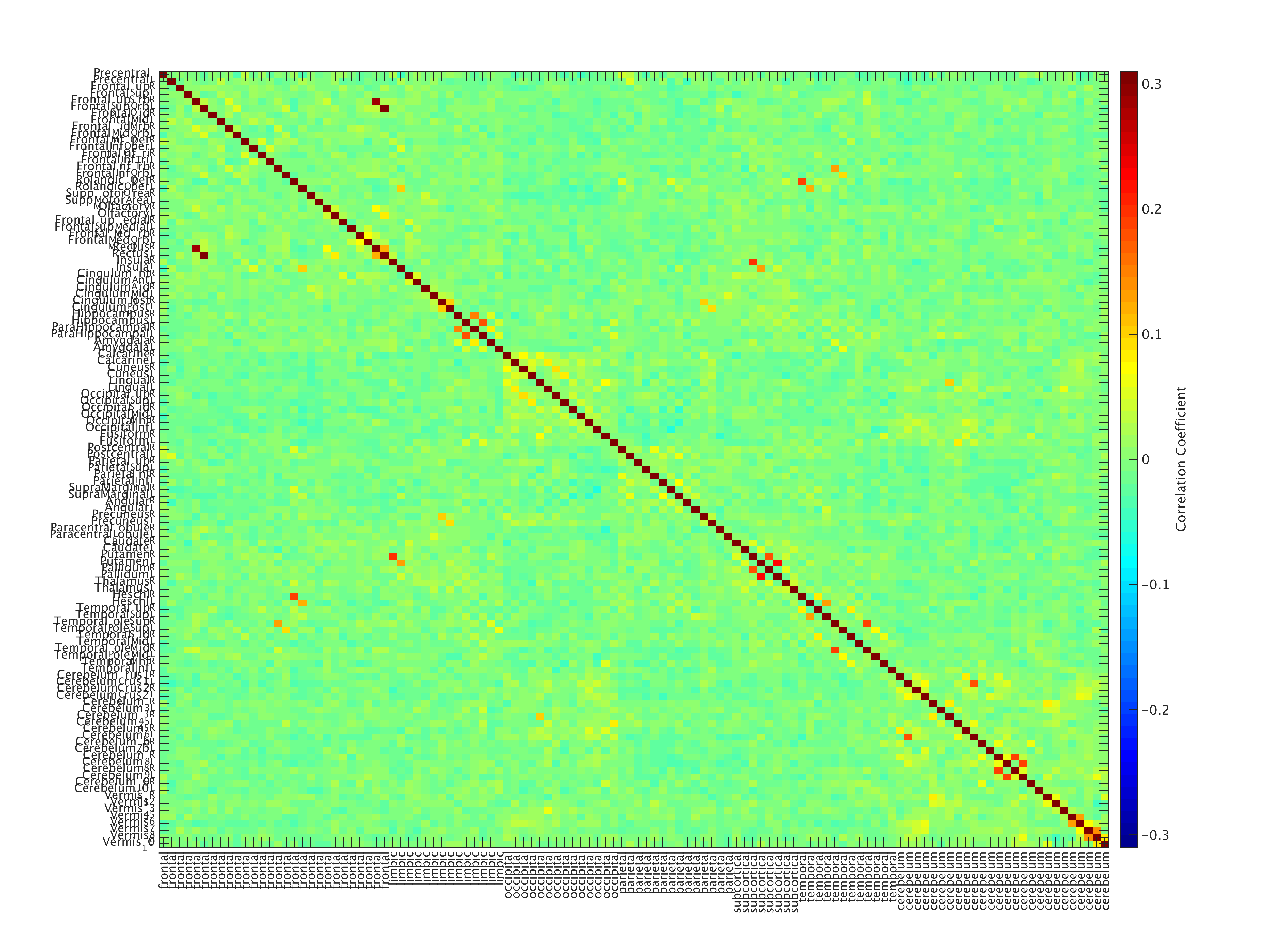

Supplement: Supplementary file 1. — This zip file contains high resolution images of the adjacency matrices for the MEG connectivity analysis suggested by the editor and reviewers. DOI: http://dx.doi.org/10.7554/eLife.23608.021 [file elife-23608-supp1.zip › hi-res_adjacency_matrices/beta/downsampled/raw/beta.ave.aal.thr.raw.z.downsampled.png]

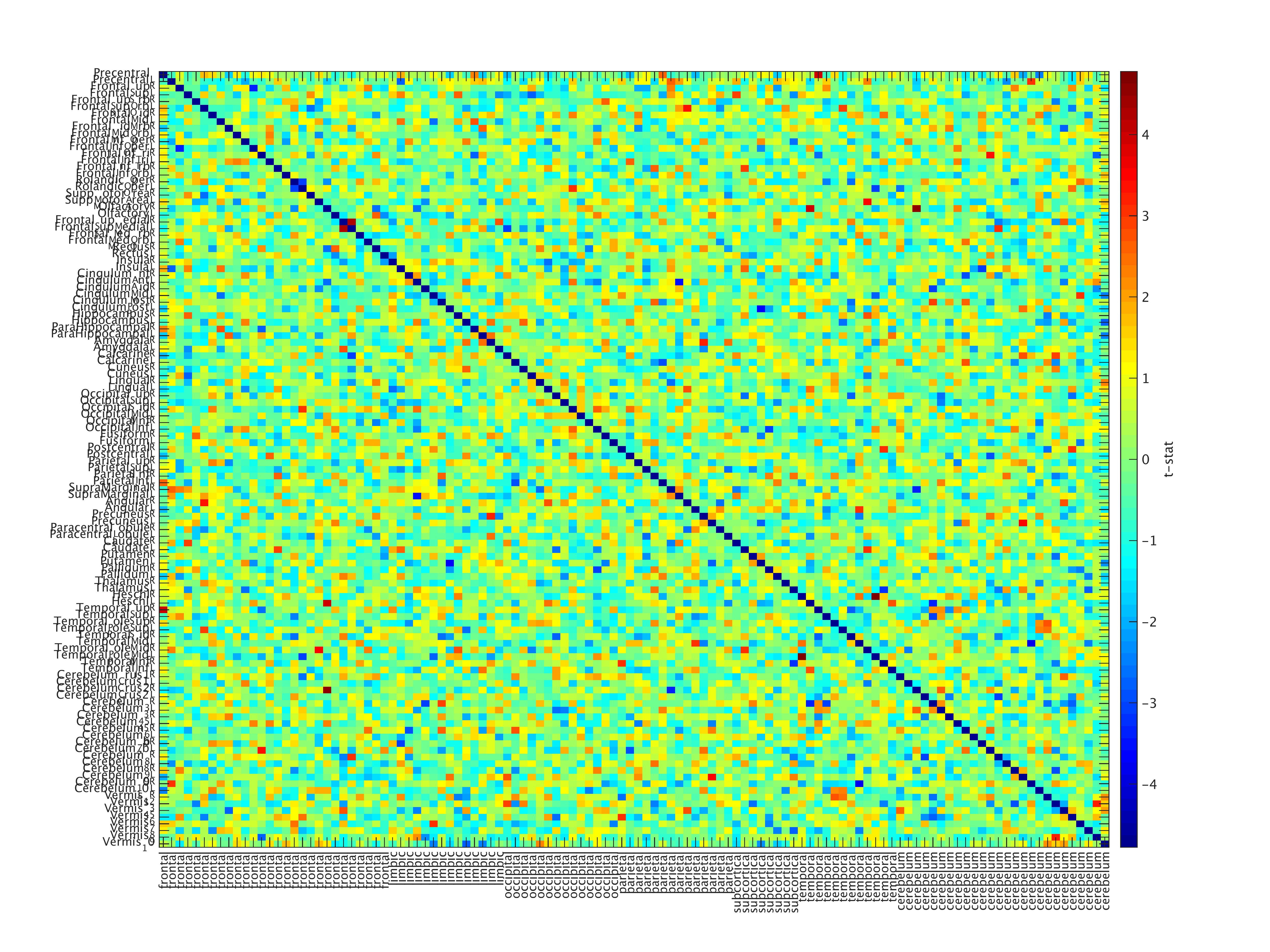

Supplement: Supplementary file 1. — This zip file contains high resolution images of the adjacency matrices for the MEG connectivity analysis suggested by the editor and reviewers. DOI: http://dx.doi.org/10.7554/eLife.23608.021 [file elife-23608-supp1.zip › hi-res_adjacency_matrices/beta/downsampled/raw/beta.tstat.aal.raw.r.downsampled.png]

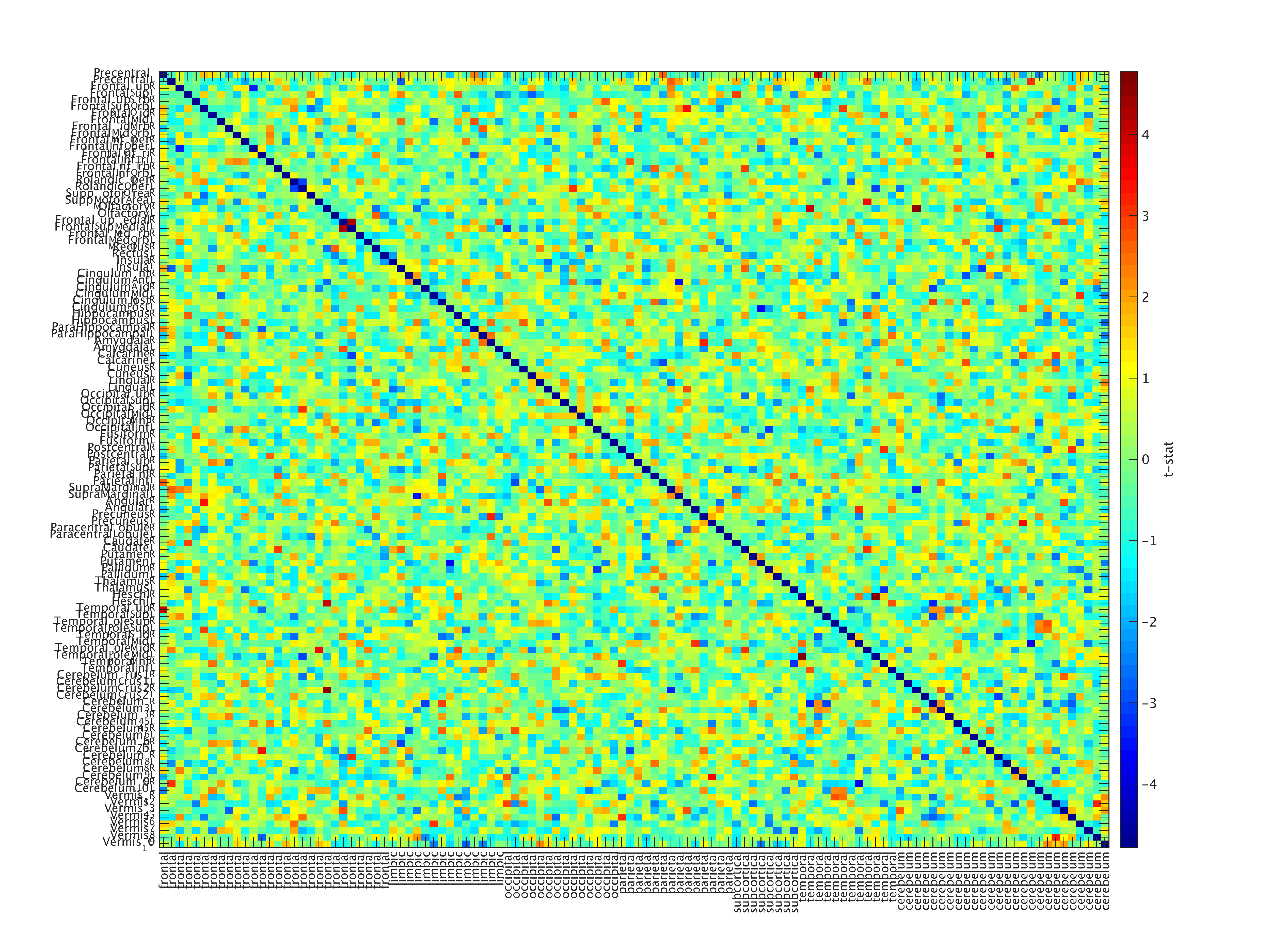

Supplement: Supplementary file 1. — This zip file contains high resolution images of the adjacency matrices for the MEG connectivity analysis suggested by the editor and reviewers. DOI: http://dx.doi.org/10.7554/eLife.23608.021 [file elife-23608-supp1.zip › hi-res_adjacency_matrices/beta/downsampled/raw/beta.tstat.aal.raw.z.downsampled.png]

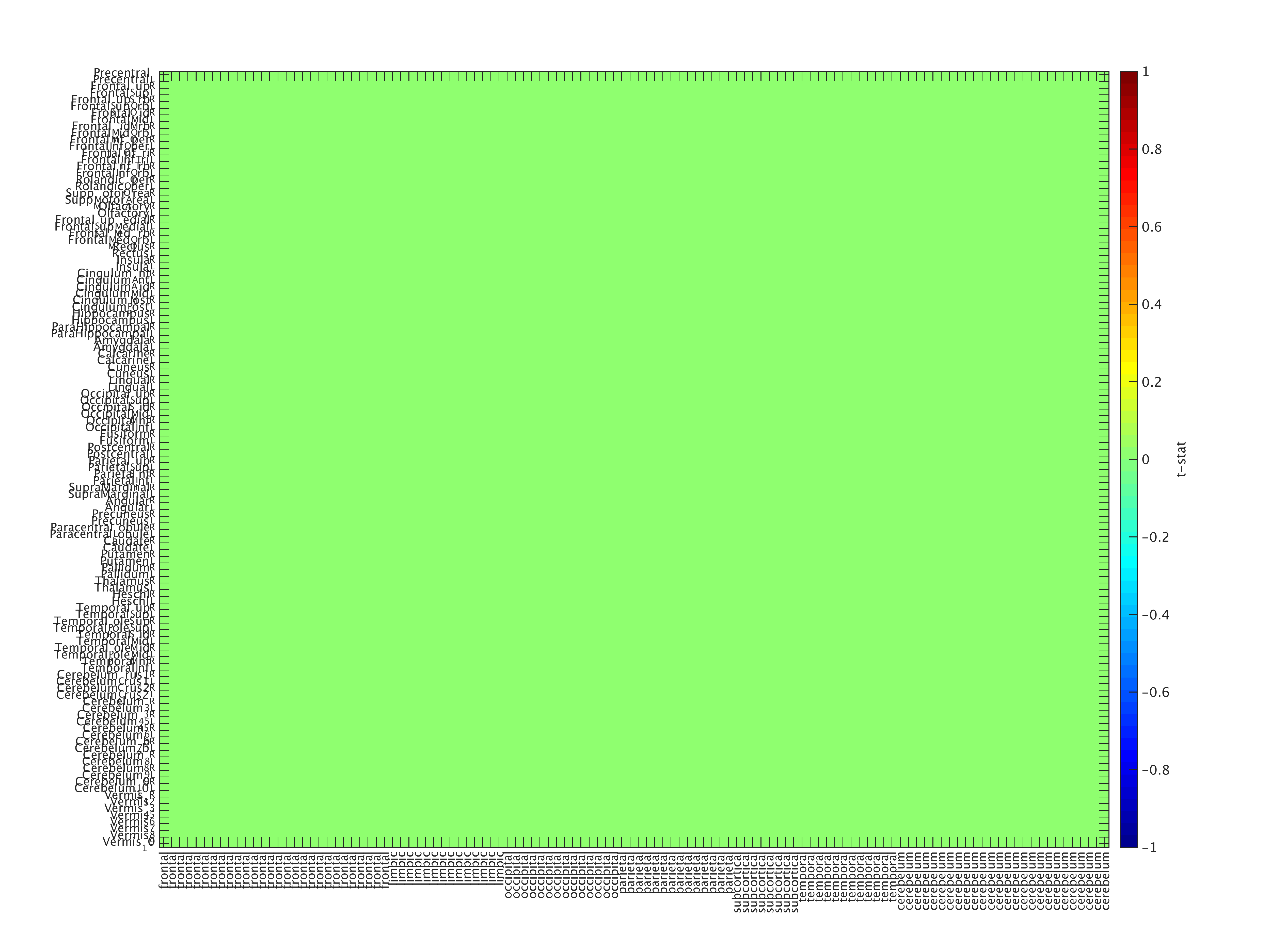

Supplement: Supplementary file 1. — This zip file contains high resolution images of the adjacency matrices for the MEG connectivity analysis suggested by the editor and reviewers. DOI: http://dx.doi.org/10.7554/eLife.23608.021 [file elife-23608-supp1.zip › hi-res_adjacency_matrices/beta/downsampled/raw/beta.t-thresh.aal.raw.r.downsampled.png]

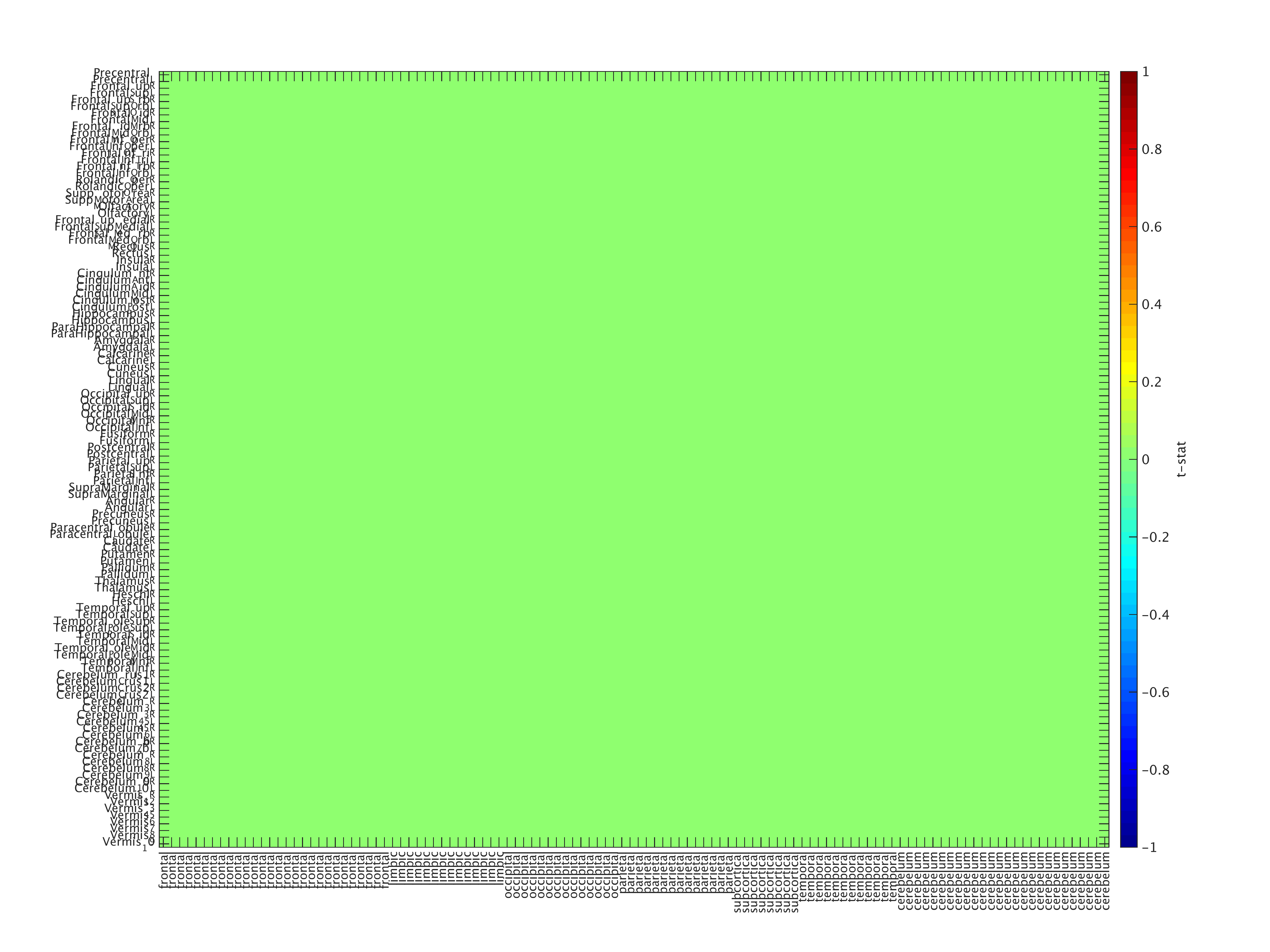

Supplement: Supplementary file 1. — This zip file contains high resolution images of the adjacency matrices for the MEG connectivity analysis suggested by the editor and reviewers. DOI: http://dx.doi.org/10.7554/eLife.23608.021 [file elife-23608-supp1.zip › hi-res_adjacency_matrices/beta/downsampled/raw/beta.t-thresh.aal.raw.z.downsampled.png]

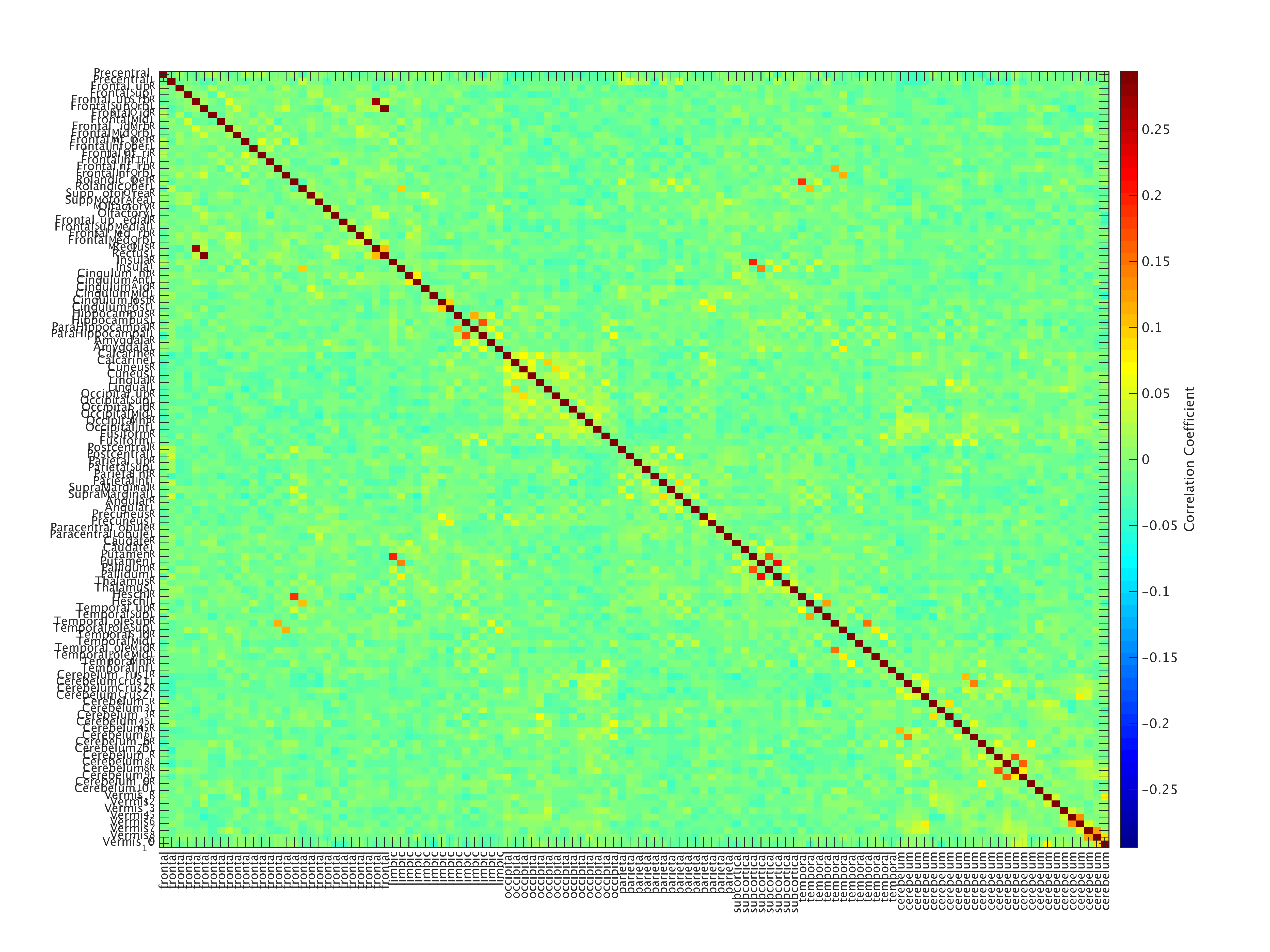

Supplement: Supplementary file 1. — This zip file contains high resolution images of the adjacency matrices for the MEG connectivity analysis suggested by the editor and reviewers. DOI: http://dx.doi.org/10.7554/eLife.23608.021 [file elife-23608-supp1.zip › hi-res_adjacency_matrices/beta/downsampled/zscore/beta.ave.aal.saf.zscore.r.downsampled.png]

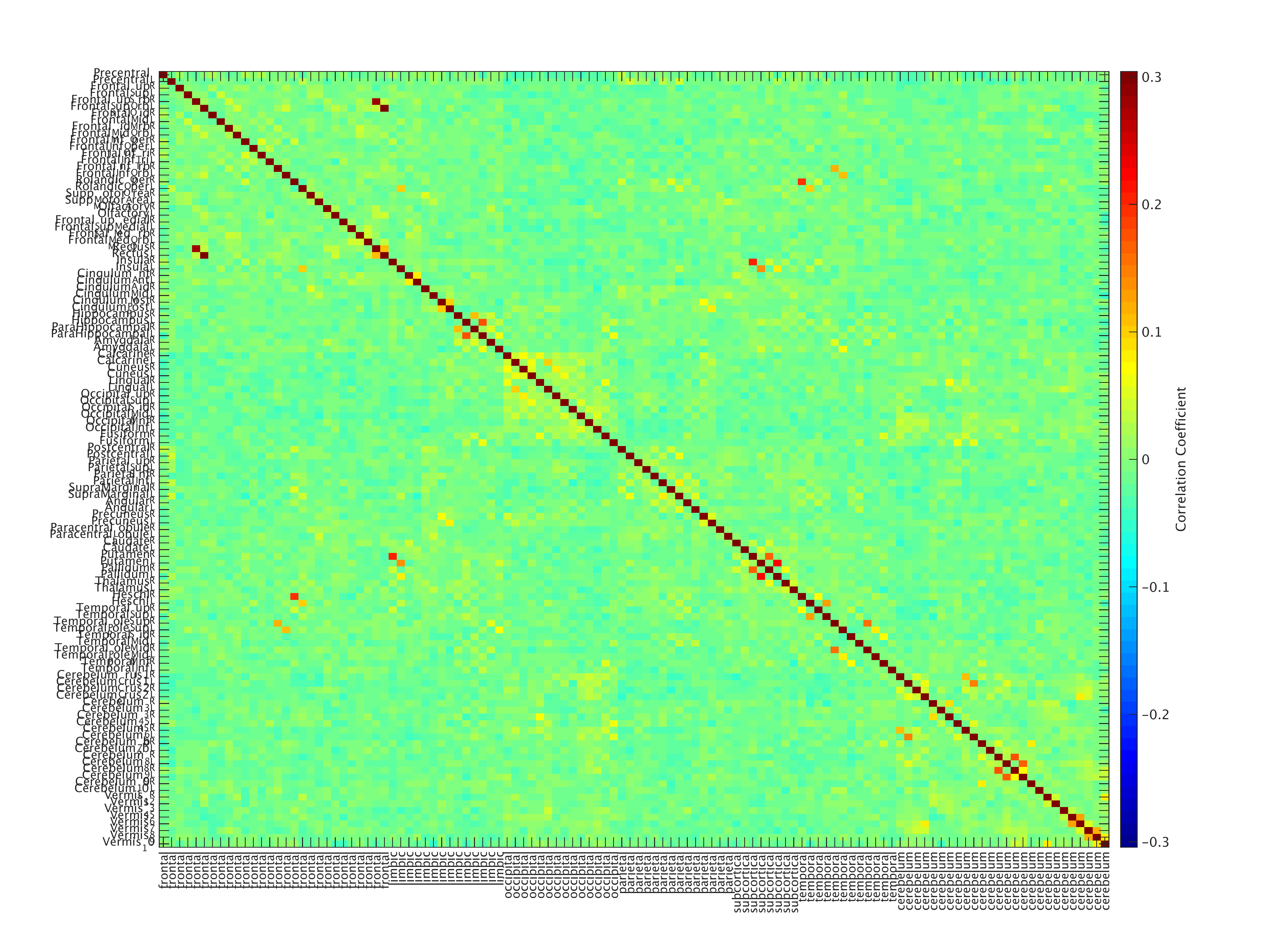

Supplement: Supplementary file 1. — This zip file contains high resolution images of the adjacency matrices for the MEG connectivity analysis suggested by the editor and reviewers. DOI: http://dx.doi.org/10.7554/eLife.23608.021 [file elife-23608-supp1.zip › hi-res_adjacency_matrices/beta/downsampled/zscore/beta.ave.aal.saf.zscore.z.downsampled.png]

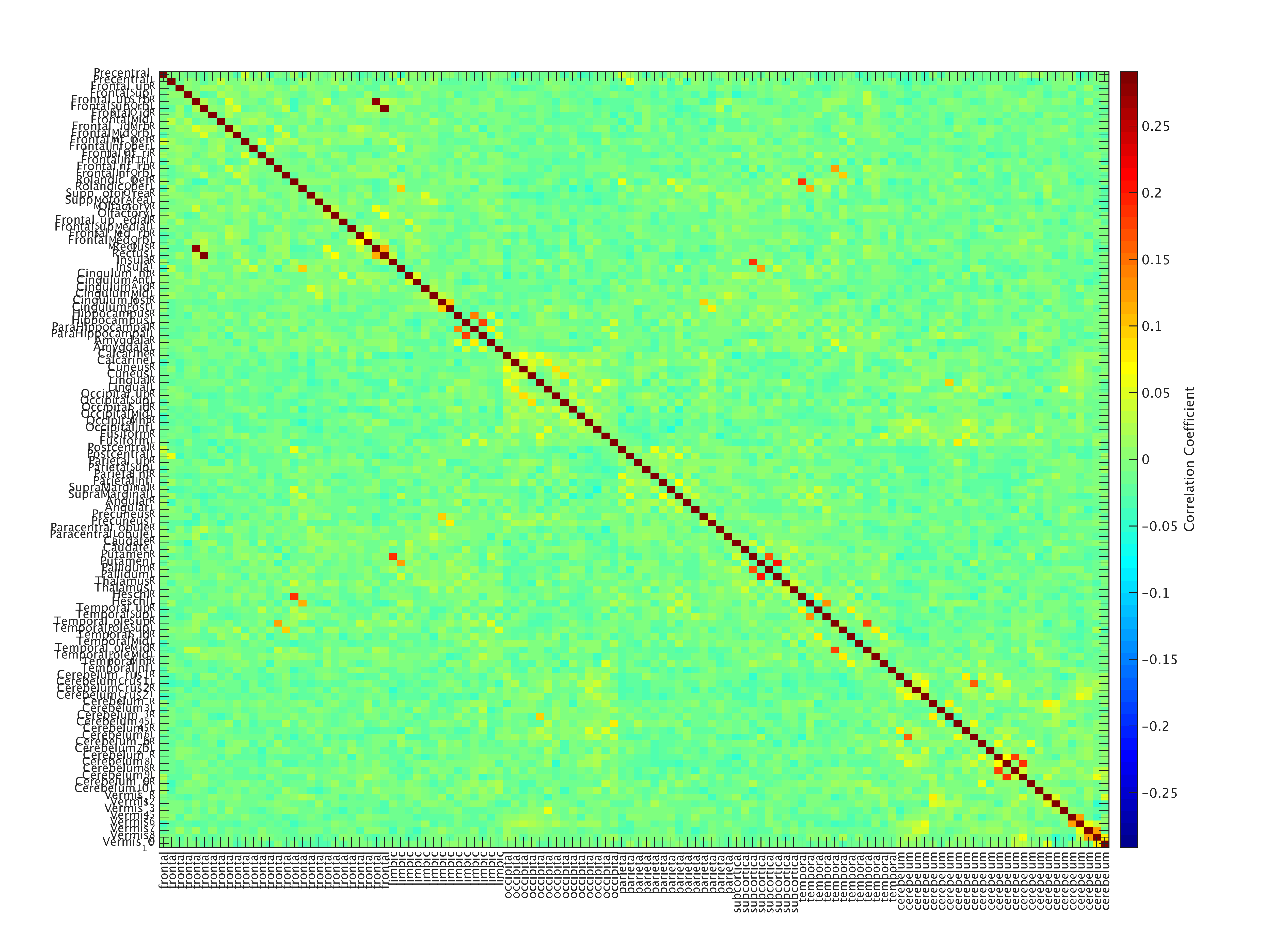

Supplement: Supplementary file 1. — This zip file contains high resolution images of the adjacency matrices for the MEG connectivity analysis suggested by the editor and reviewers. DOI: http://dx.doi.org/10.7554/eLife.23608.021 [file elife-23608-supp1.zip › hi-res_adjacency_matrices/beta/downsampled/zscore/beta.ave.aal.thr.zscore.r.downsampled.png]

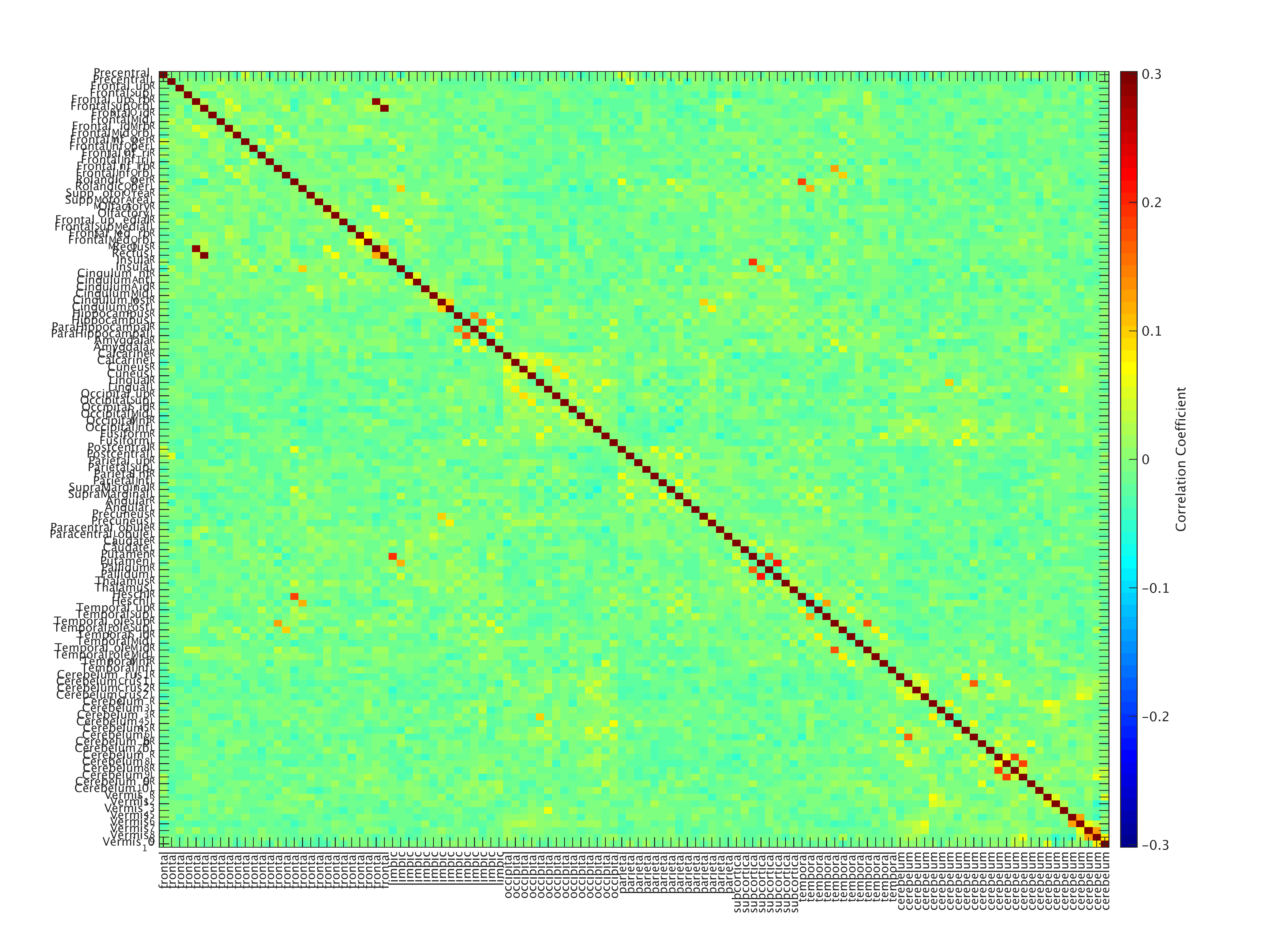

Supplement: Supplementary file 1. — This zip file contains high resolution images of the adjacency matrices for the MEG connectivity analysis suggested by the editor and reviewers. DOI: http://dx.doi.org/10.7554/eLife.23608.021 [file elife-23608-supp1.zip › hi-res_adjacency_matrices/beta/downsampled/zscore/beta.ave.aal.thr.zscore.z.downsampled.png]

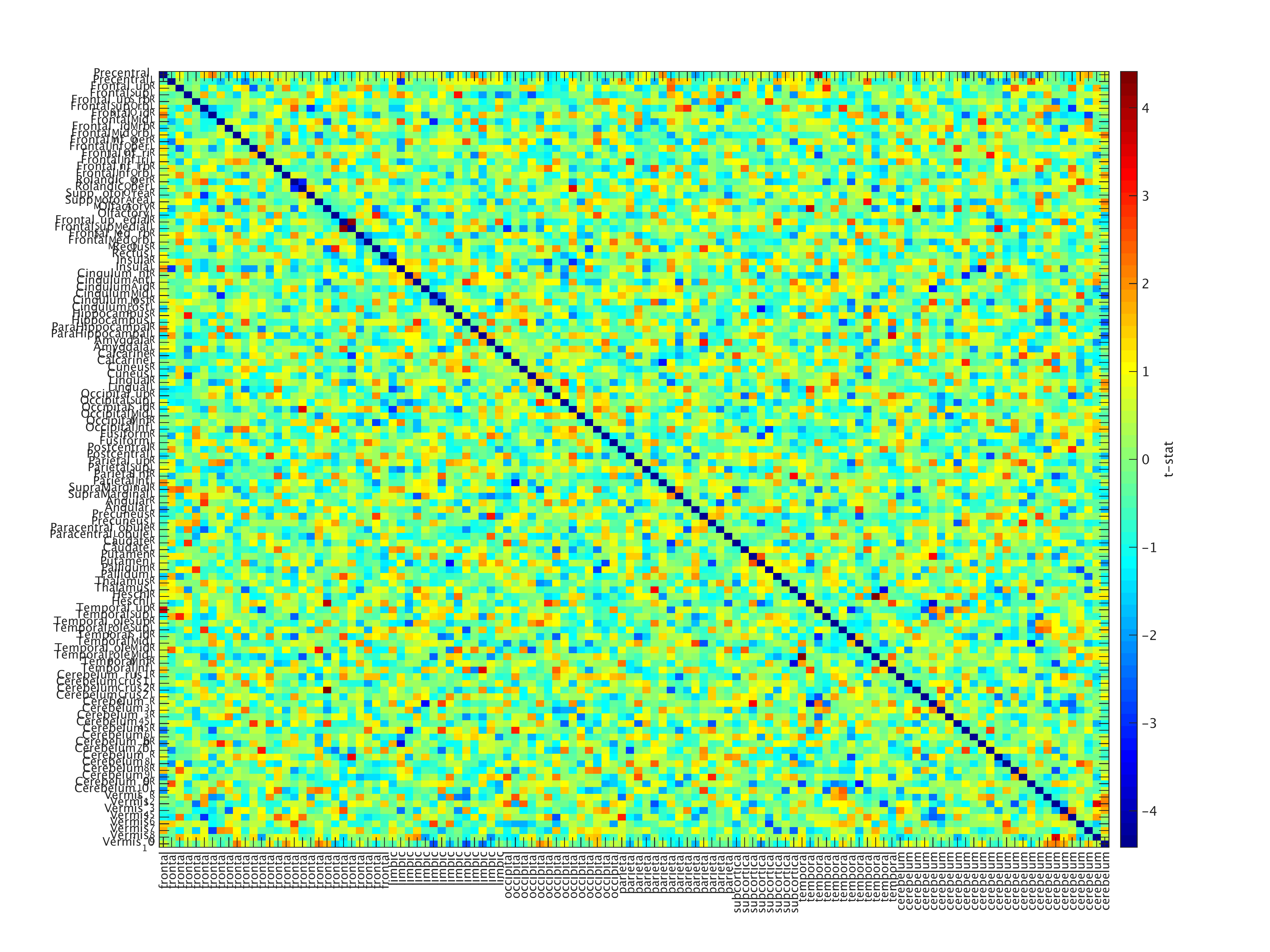

Supplement: Supplementary file 1. — This zip file contains high resolution images of the adjacency matrices for the MEG connectivity analysis suggested by the editor and reviewers. DOI: http://dx.doi.org/10.7554/eLife.23608.021 [file elife-23608-supp1.zip › hi-res_adjacency_matrices/beta/downsampled/zscore/beta.tstat.aal.zscore.r.downsampled.png]

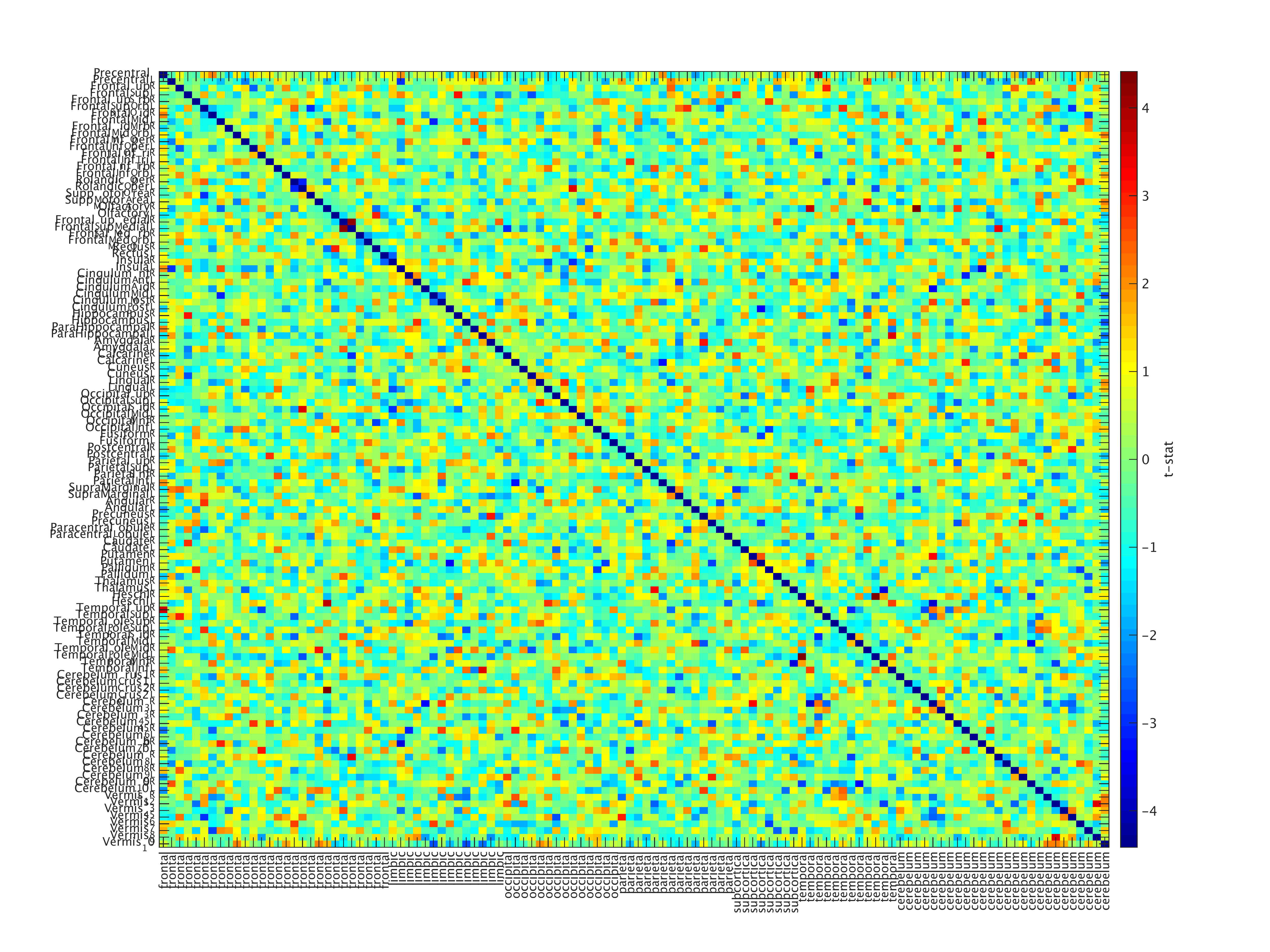

Supplement: Supplementary file 1. — This zip file contains high resolution images of the adjacency matrices for the MEG connectivity analysis suggested by the editor and reviewers. DOI: http://dx.doi.org/10.7554/eLife.23608.021 [file elife-23608-supp1.zip › hi-res_adjacency_matrices/beta/downsampled/zscore/beta.tstat.aal.zscore.z.downsampled.png]

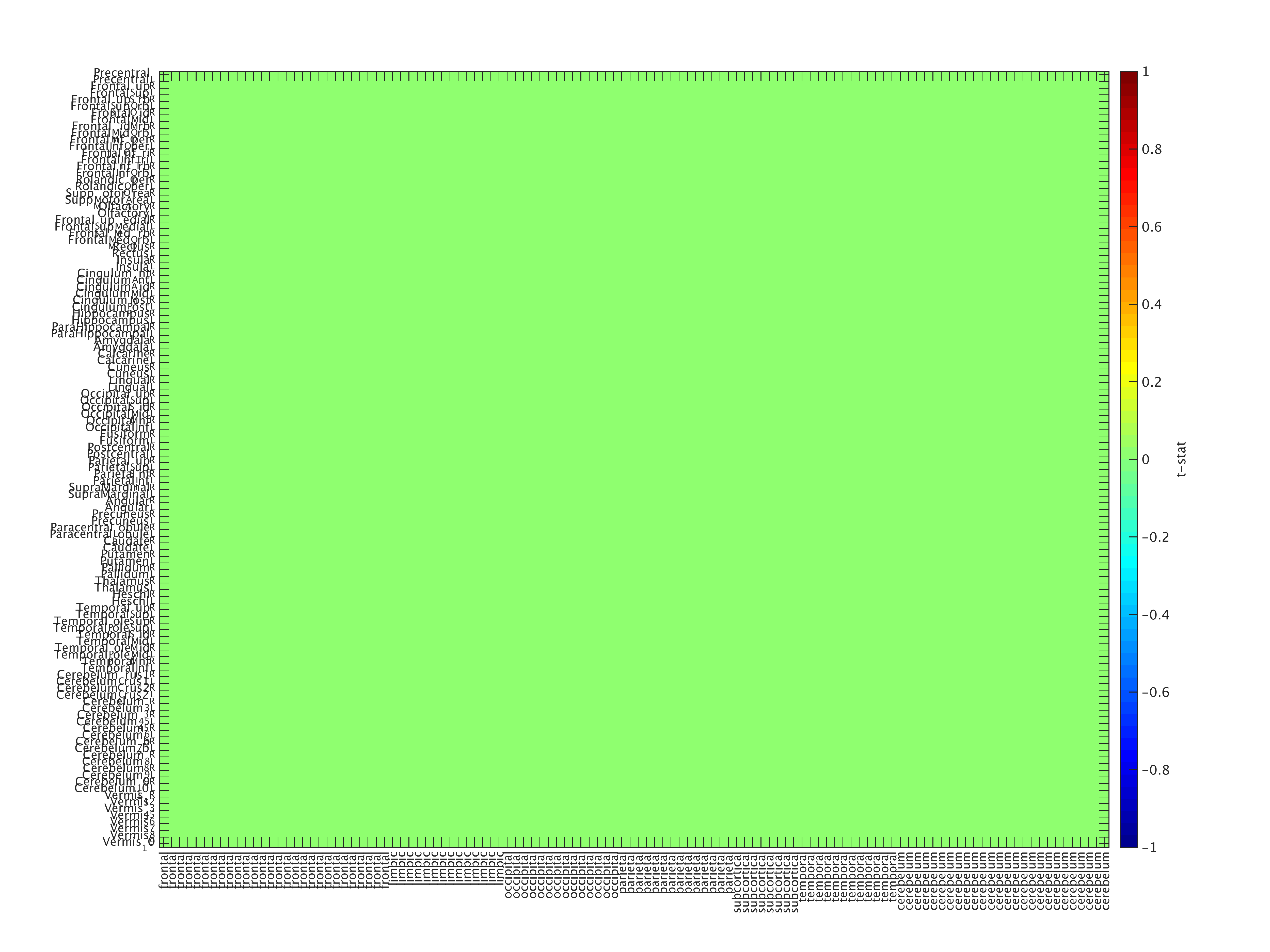

Supplement: Supplementary file 1. — This zip file contains high resolution images of the adjacency matrices for the MEG connectivity analysis suggested by the editor and reviewers. DOI: http://dx.doi.org/10.7554/eLife.23608.021 [file elife-23608-supp1.zip › hi-res_adjacency_matrices/beta/downsampled/zscore/beta.t-thresh.aal.zscore.r.downsampled.png]

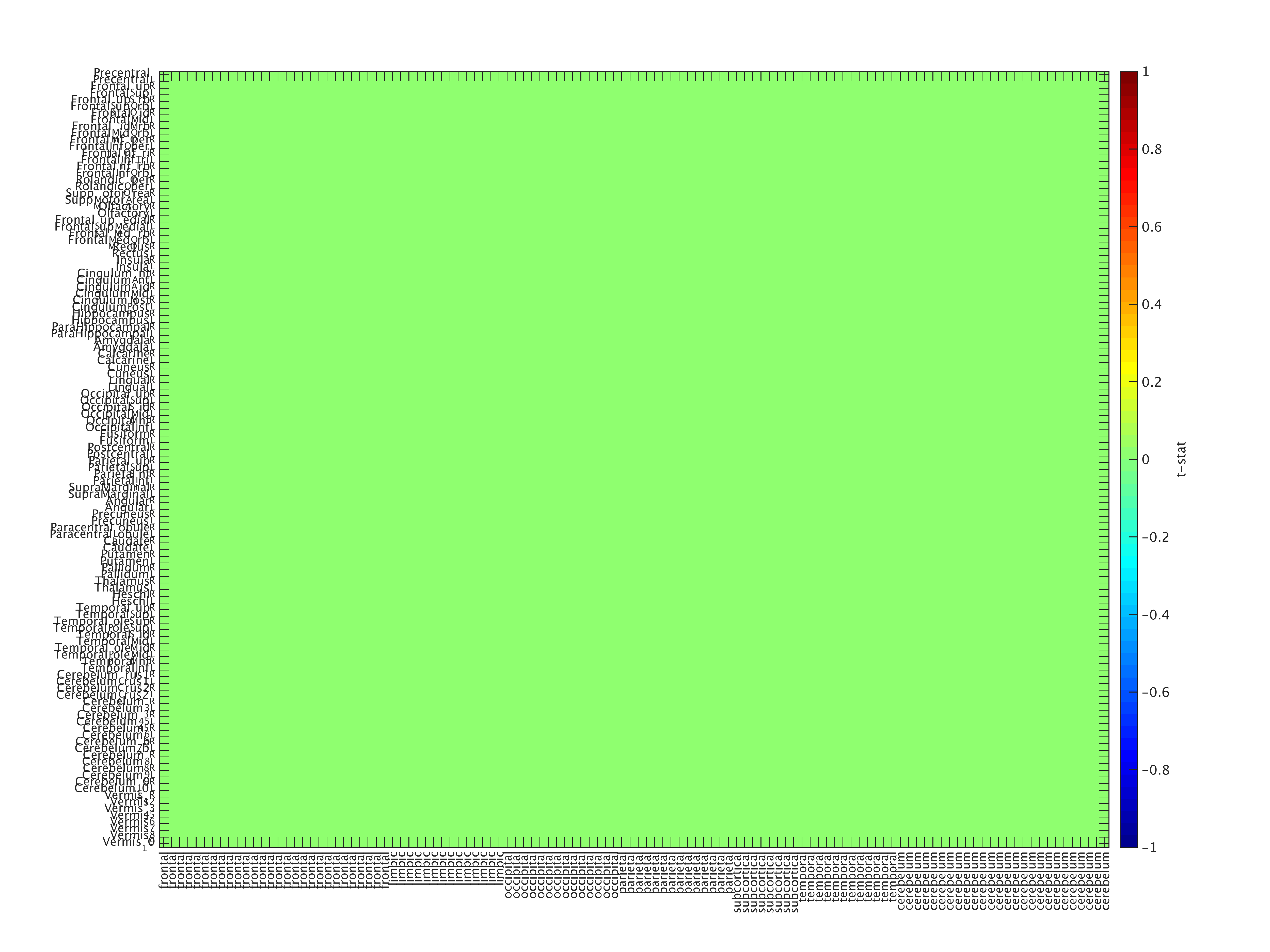

Supplement: Supplementary file 1. — This zip file contains high resolution images of the adjacency matrices for the MEG connectivity analysis suggested by the editor and reviewers. DOI: http://dx.doi.org/10.7554/eLife.23608.021 [file elife-23608-supp1.zip › hi-res_adjacency_matrices/beta/downsampled/zscore/beta.t-thresh.aal.zscore.z.downsampled.png]

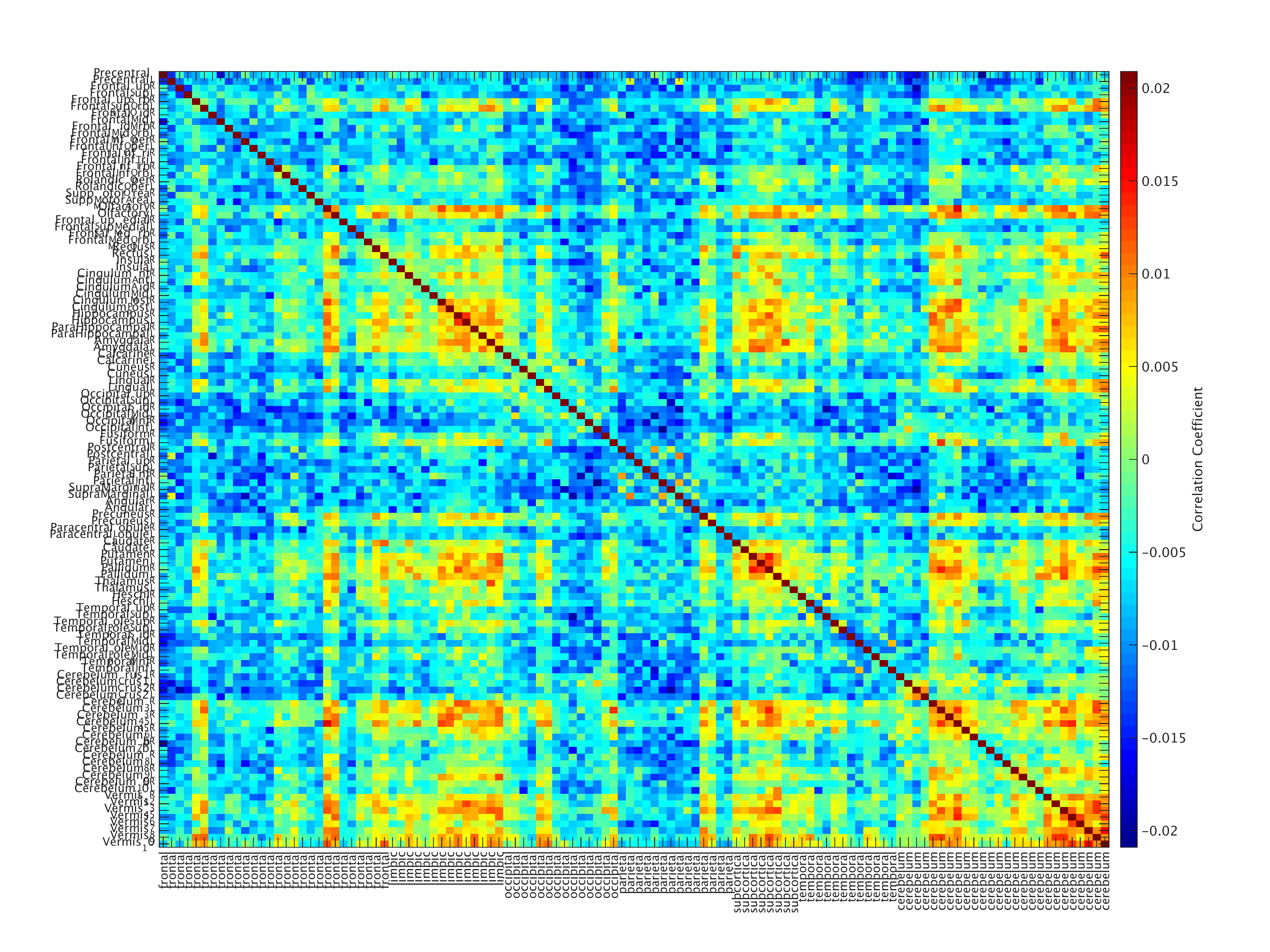

Supplement: Supplementary file 1. — This zip file contains high resolution images of the adjacency matrices for the MEG connectivity analysis suggested by the editor and reviewers. DOI: http://dx.doi.org/10.7554/eLife.23608.021 [file elife-23608-supp1.zip › hi-res_adjacency_matrices/beta/not_downsampled/raw/beta.ave.aal.saf.raw.r.not_downsampled.png]

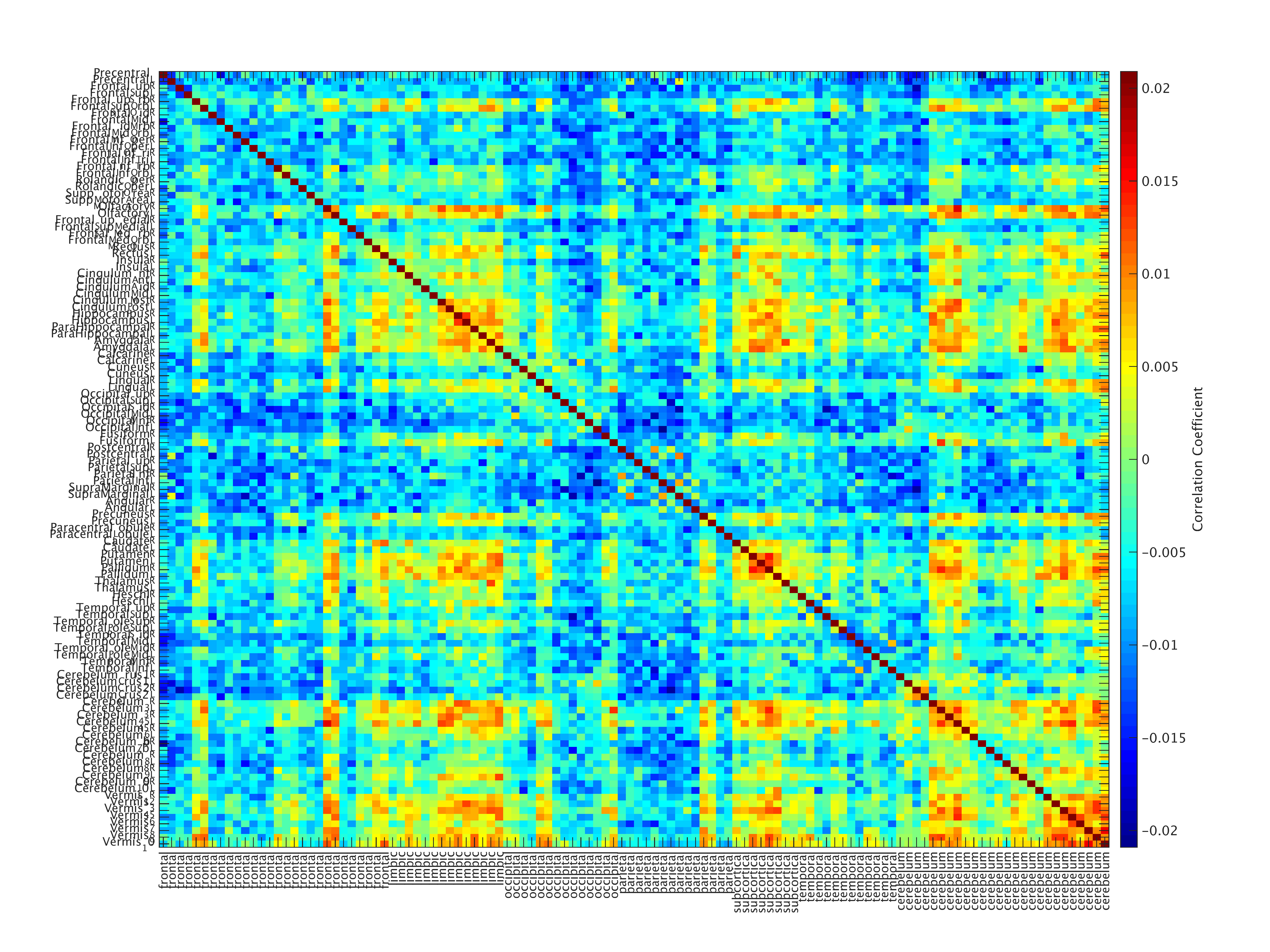

Supplement: Supplementary file 1. — This zip file contains high resolution images of the adjacency matrices for the MEG connectivity analysis suggested by the editor and reviewers. DOI: http://dx.doi.org/10.7554/eLife.23608.021 [file elife-23608-supp1.zip › hi-res_adjacency_matrices/beta/not_downsampled/raw/beta.ave.aal.saf.raw.z.not_downsampled.png]

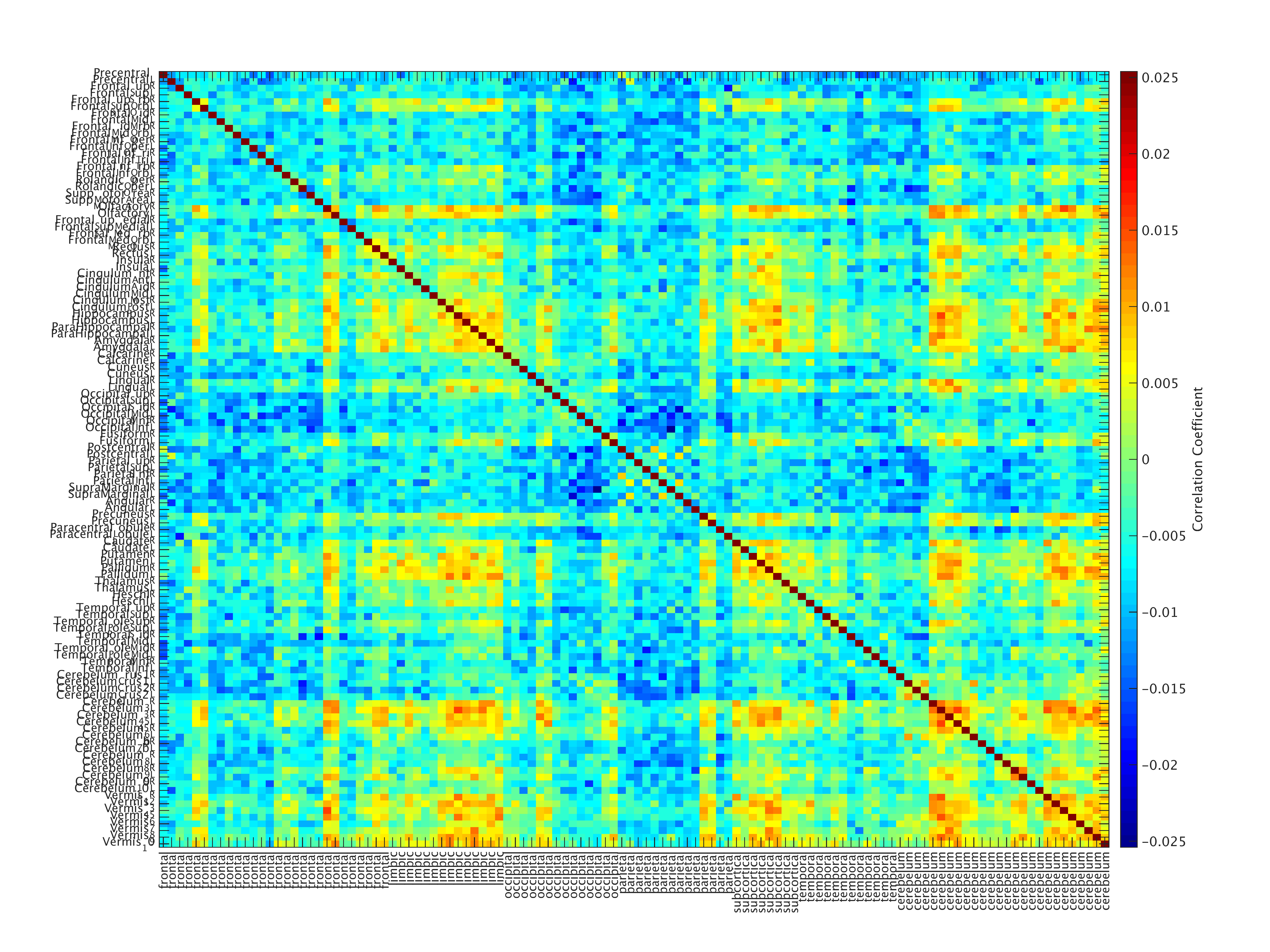

Supplement: Supplementary file 1. — This zip file contains high resolution images of the adjacency matrices for the MEG connectivity analysis suggested by the editor and reviewers. DOI: http://dx.doi.org/10.7554/eLife.23608.021 [file elife-23608-supp1.zip › hi-res_adjacency_matrices/beta/not_downsampled/raw/beta.ave.aal.thr.raw.r.not_downsampled.png]

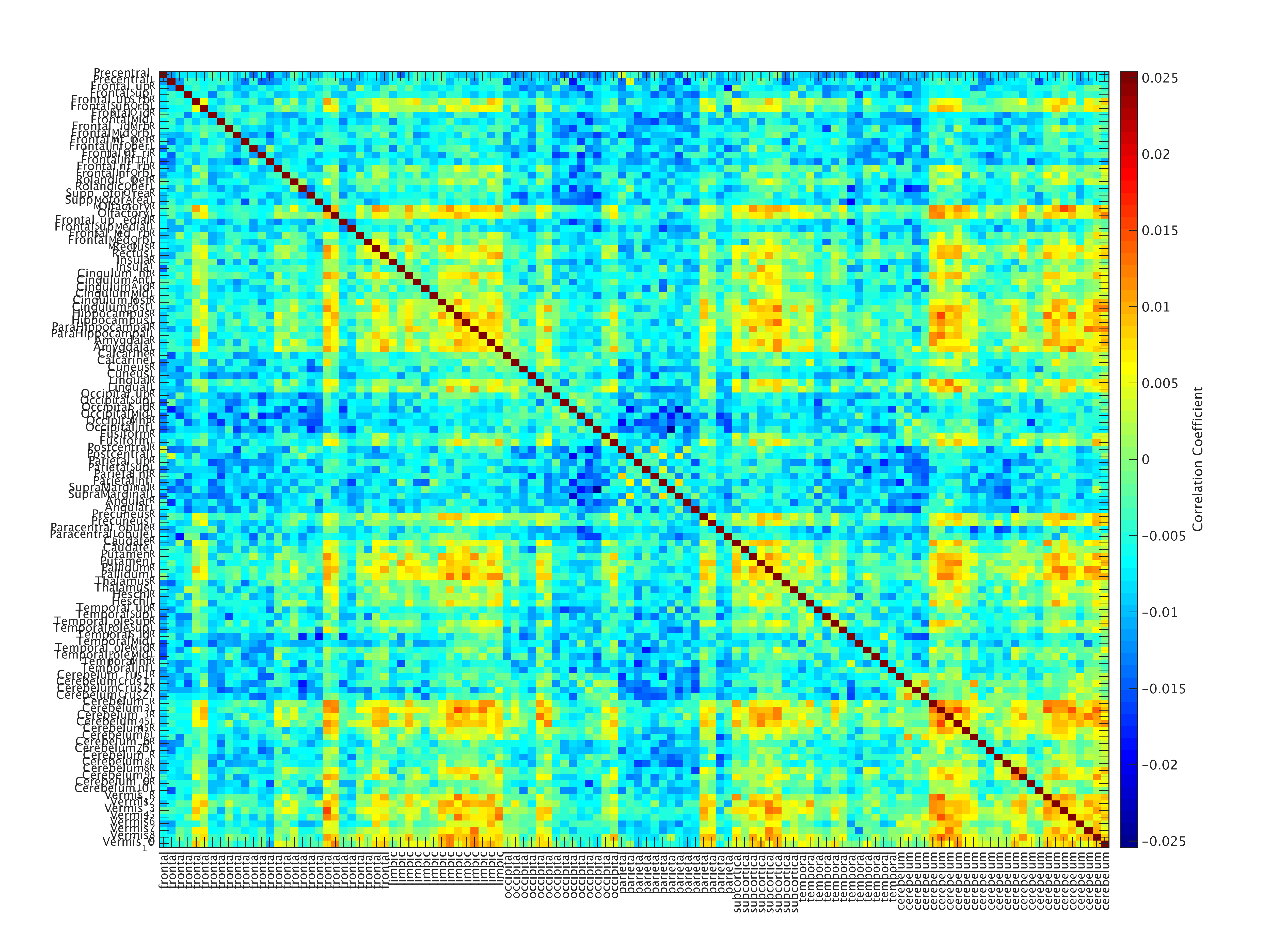

Supplement: Supplementary file 1. — This zip file contains high resolution images of the adjacency matrices for the MEG connectivity analysis suggested by the editor and reviewers. DOI: http://dx.doi.org/10.7554/eLife.23608.021 [file elife-23608-supp1.zip › hi-res_adjacency_matrices/beta/not_downsampled/raw/beta.ave.aal.thr.raw.z.not_downsampled.png]

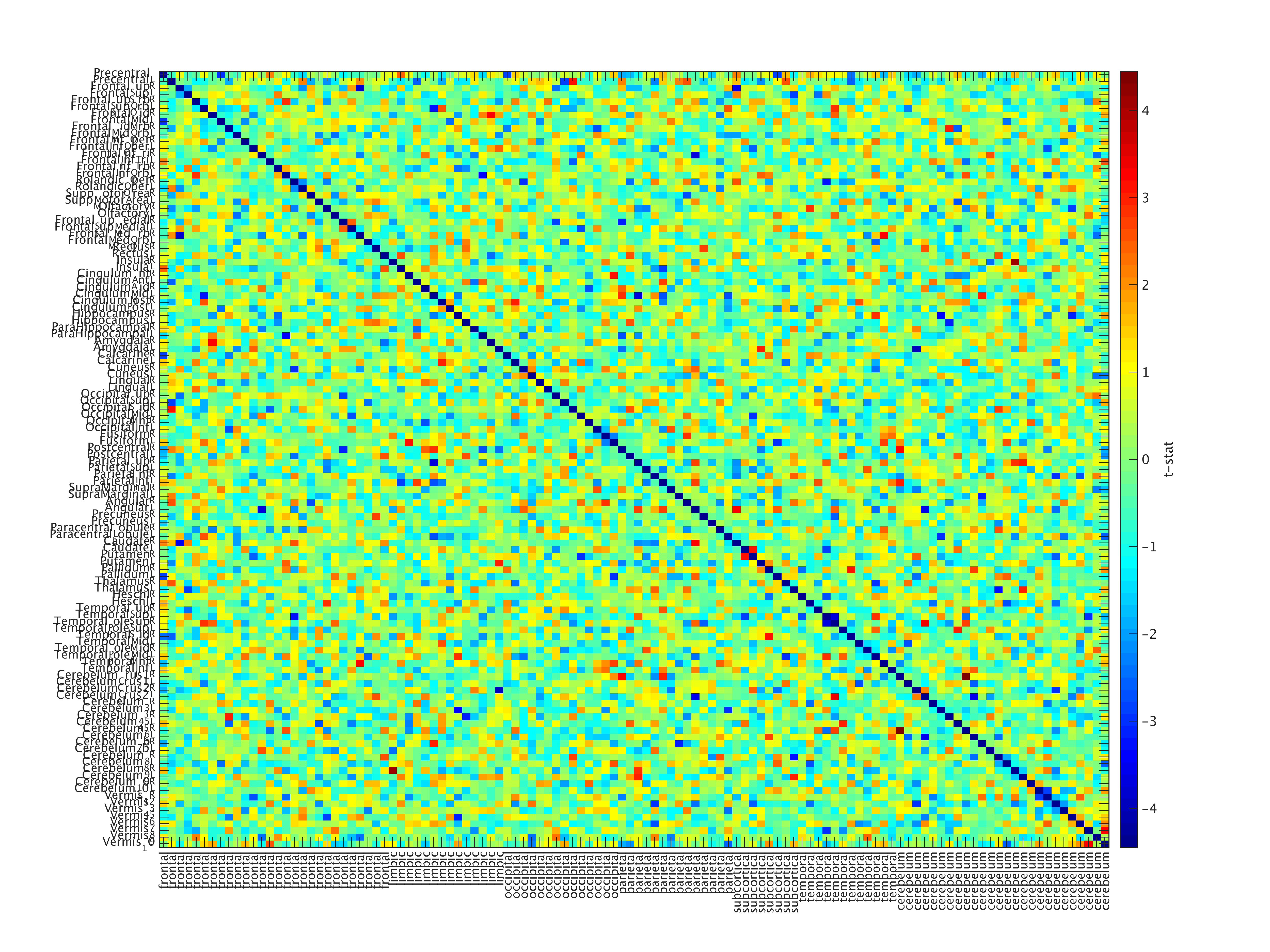

Supplement: Supplementary file 1. — This zip file contains high resolution images of the adjacency matrices for the MEG connectivity analysis suggested by the editor and reviewers. DOI: http://dx.doi.org/10.7554/eLife.23608.021 [file elife-23608-supp1.zip › hi-res_adjacency_matrices/beta/not_downsampled/raw/beta.tstat.aal.raw.r.not_downsampled.png]

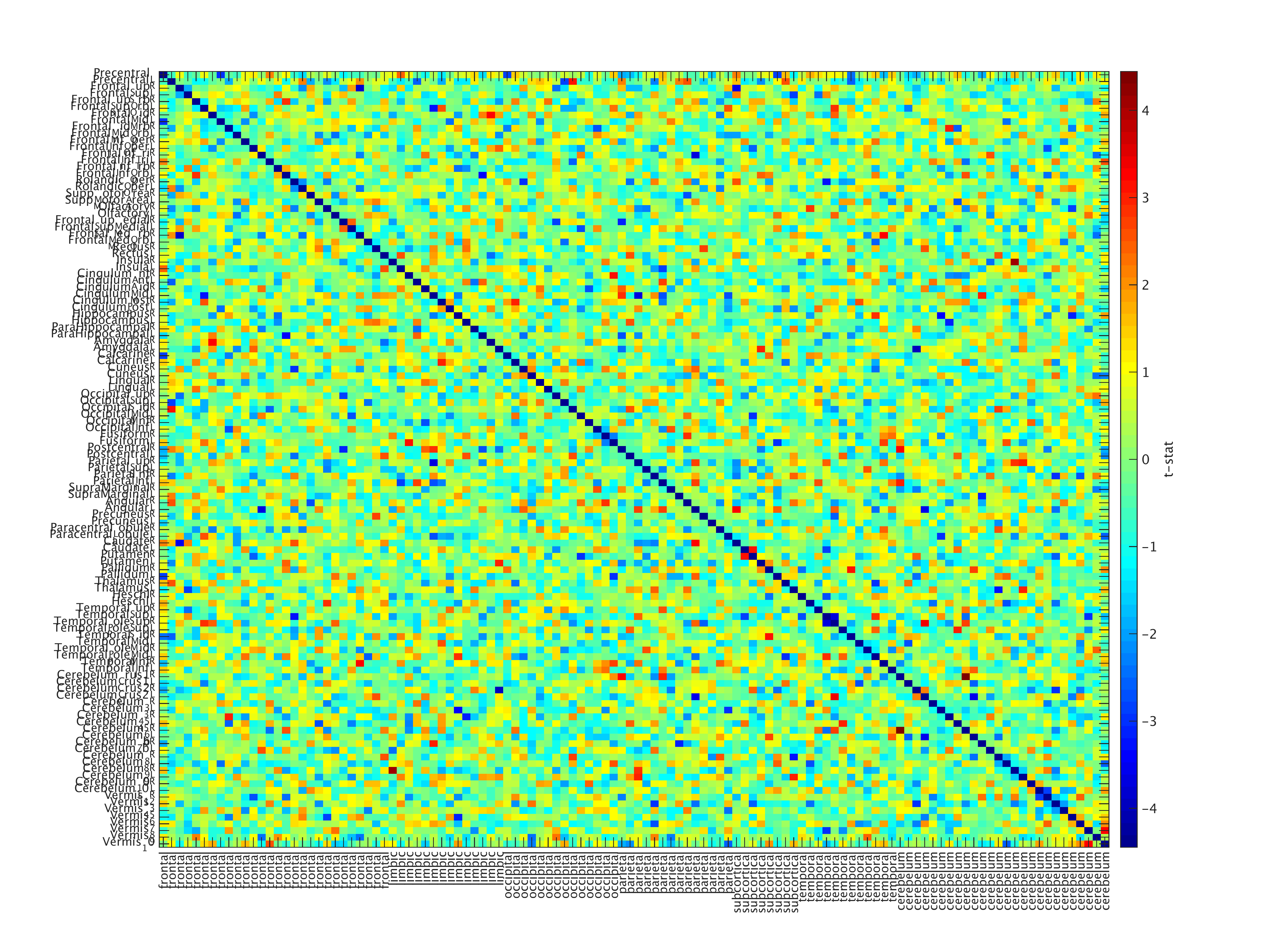

Supplement: Supplementary file 1. — This zip file contains high resolution images of the adjacency matrices for the MEG connectivity analysis suggested by the editor and reviewers. DOI: http://dx.doi.org/10.7554/eLife.23608.021 [file elife-23608-supp1.zip › hi-res_adjacency_matrices/beta/not_downsampled/raw/beta.tstat.aal.raw.z.not_downsampled.png]

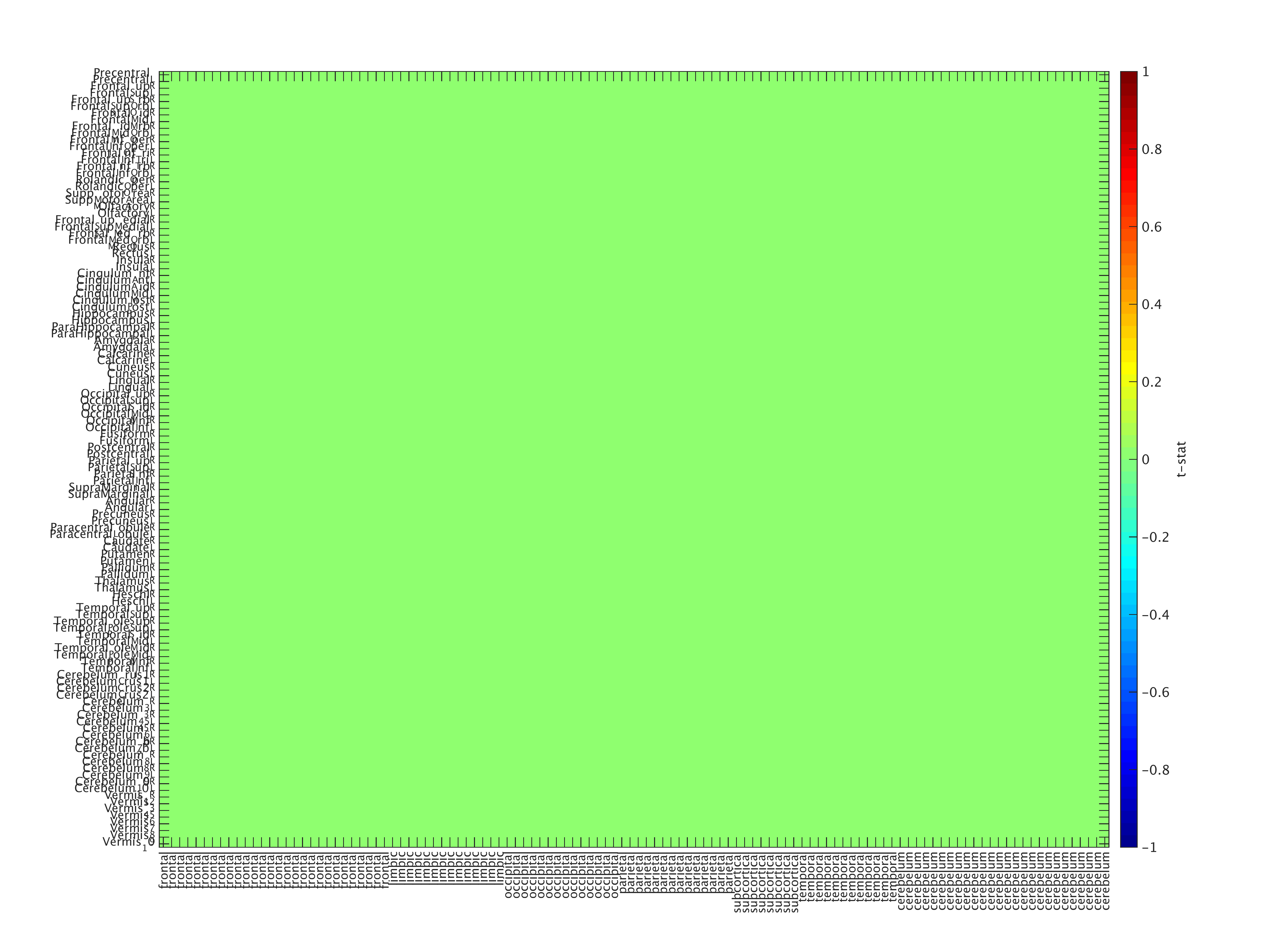

Supplement: Supplementary file 1. — This zip file contains high resolution images of the adjacency matrices for the MEG connectivity analysis suggested by the editor and reviewers. DOI: http://dx.doi.org/10.7554/eLife.23608.021 [file elife-23608-supp1.zip › hi-res_adjacency_matrices/beta/not_downsampled/raw/beta.t-thresh.aal.raw.r.not_downsampled.png]

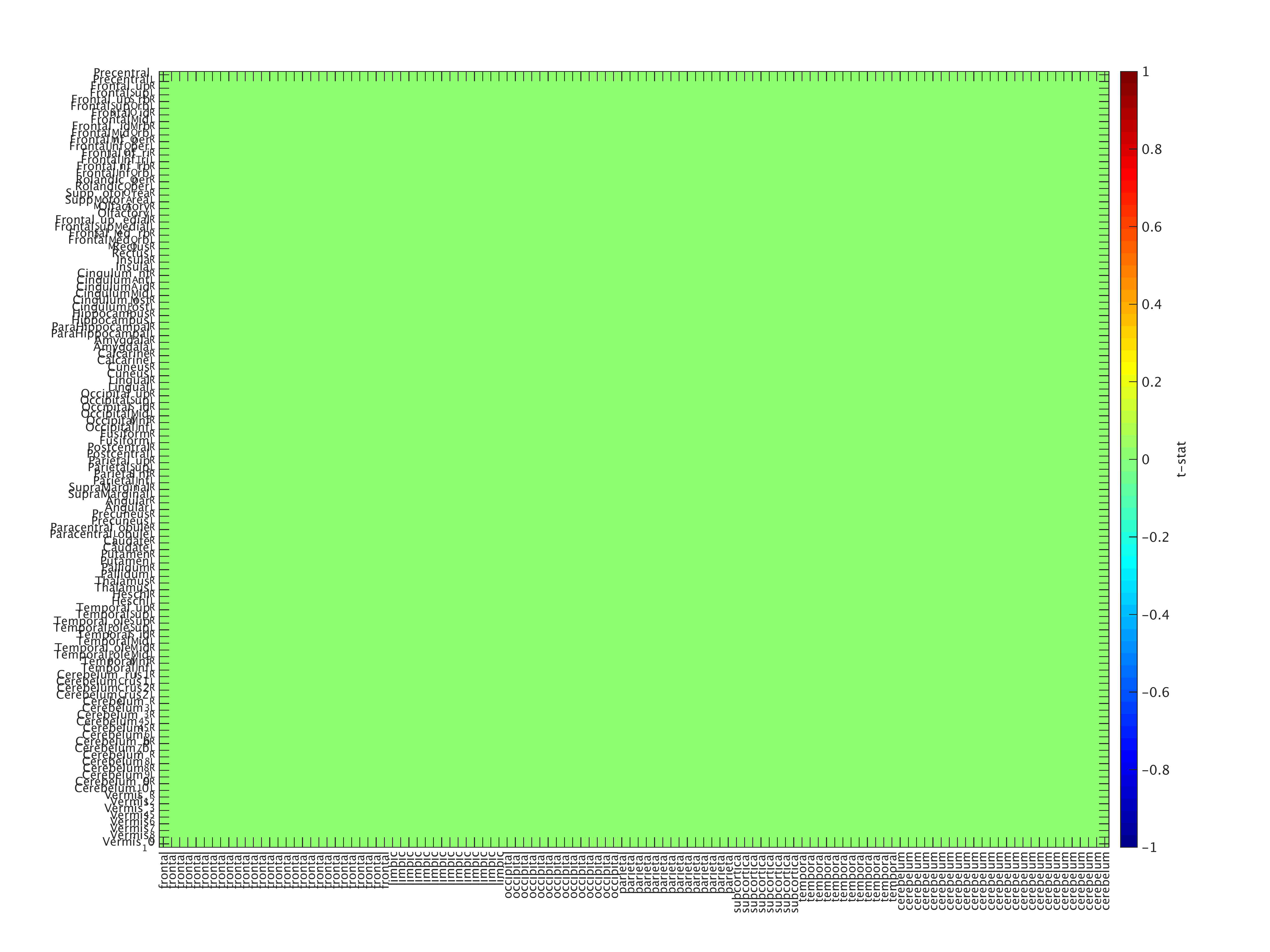

Supplement: Supplementary file 1. — This zip file contains high resolution images of the adjacency matrices for the MEG connectivity analysis suggested by the editor and reviewers. DOI: http://dx.doi.org/10.7554/eLife.23608.021 [file elife-23608-supp1.zip › hi-res_adjacency_matrices/beta/not_downsampled/raw/beta.t-thresh.aal.raw.z.not_downsampled.png]

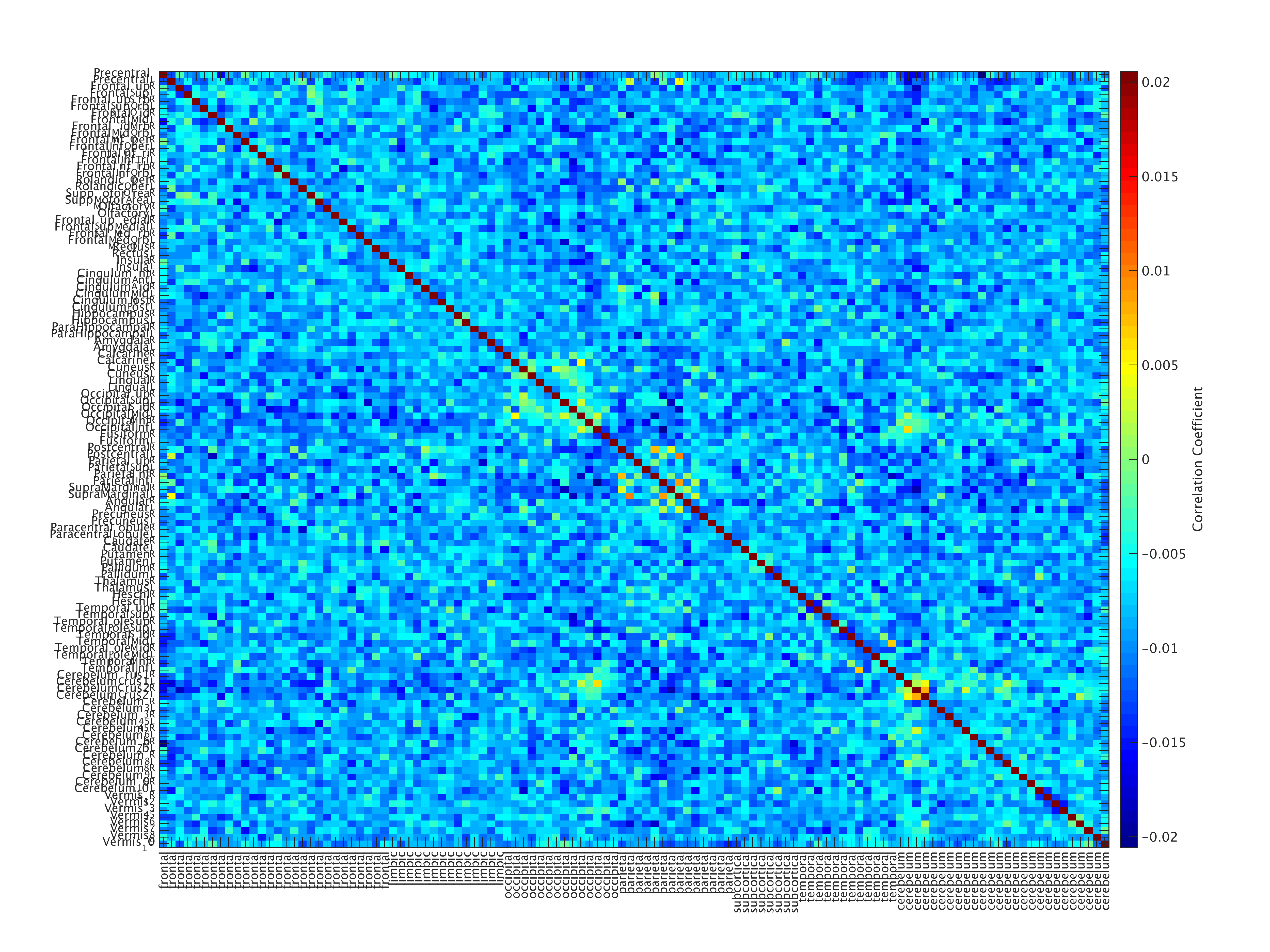

Supplement: Supplementary file 1. — This zip file contains high resolution images of the adjacency matrices for the MEG connectivity analysis suggested by the editor and reviewers. DOI: http://dx.doi.org/10.7554/eLife.23608.021 [file elife-23608-supp1.zip › hi-res_adjacency_matrices/beta/not_downsampled/zscore/beta.ave.aal.saf.zscore.r.not_downsampled.png]

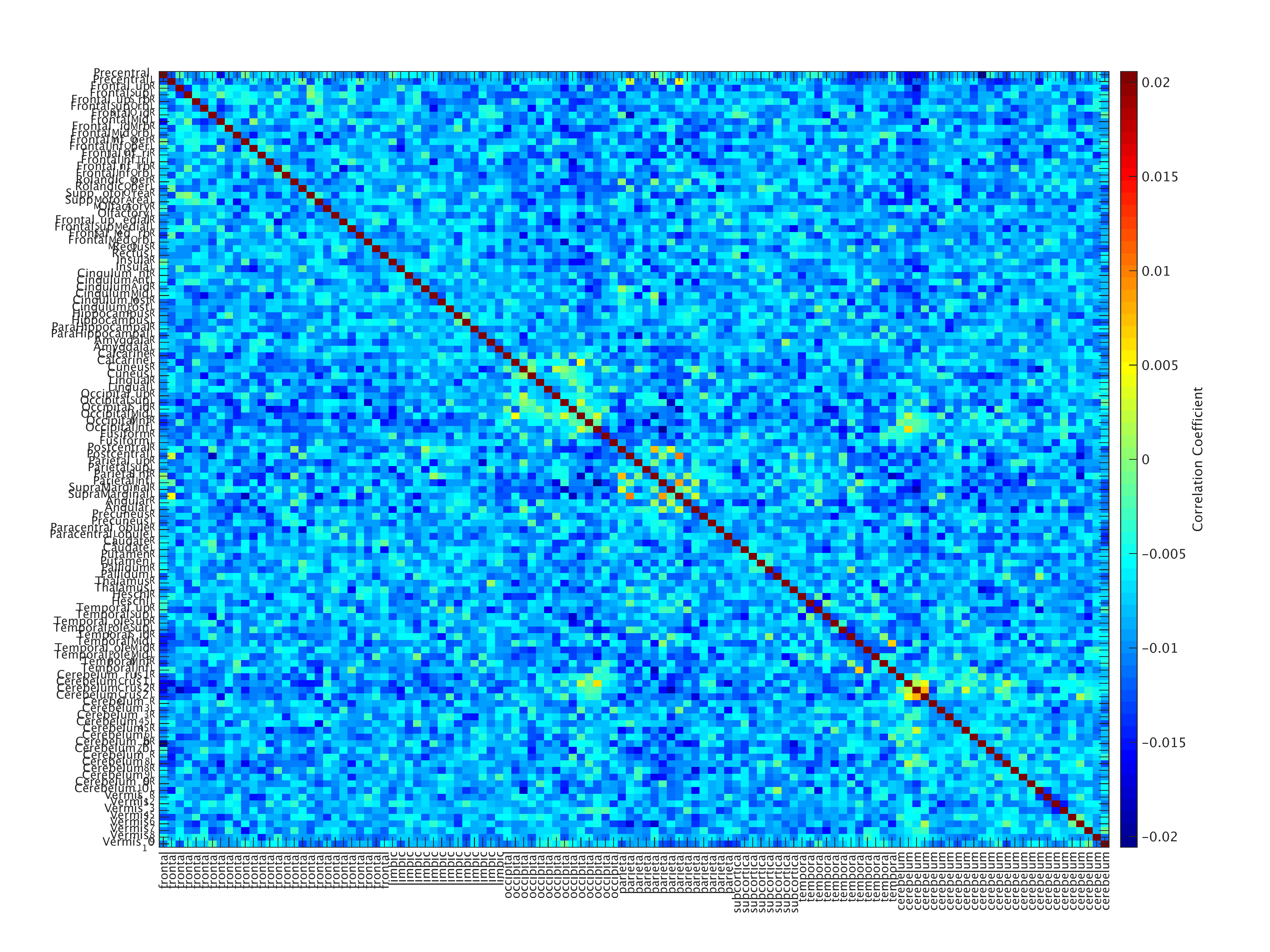

Supplement: Supplementary file 1. — This zip file contains high resolution images of the adjacency matrices for the MEG connectivity analysis suggested by the editor and reviewers. DOI: http://dx.doi.org/10.7554/eLife.23608.021 [file elife-23608-supp1.zip › hi-res_adjacency_matrices/beta/not_downsampled/zscore/beta.ave.aal.saf.zscore.z.not_downsampled.png]

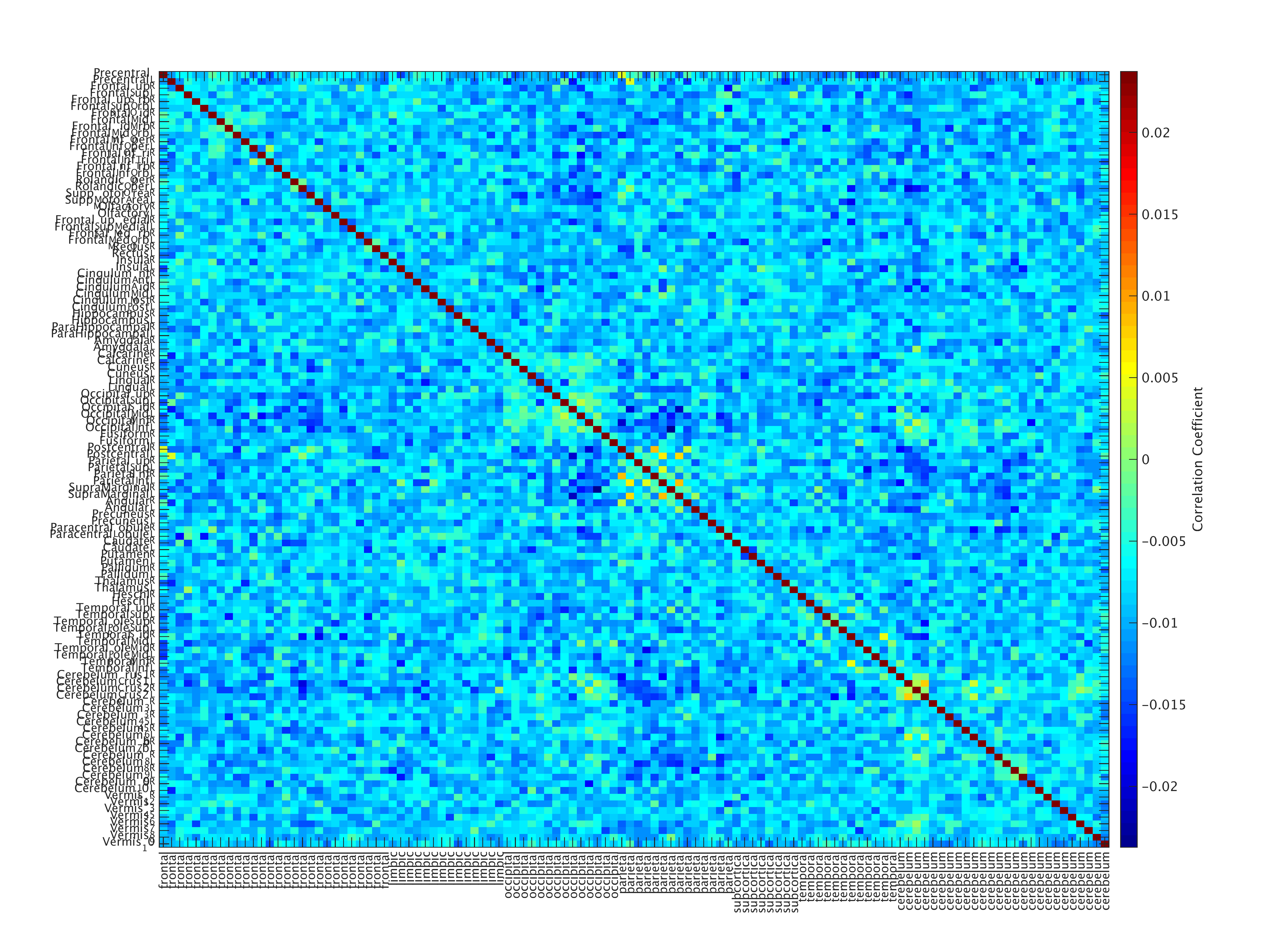

Supplement: Supplementary file 1. — This zip file contains high resolution images of the adjacency matrices for the MEG connectivity analysis suggested by the editor and reviewers. DOI: http://dx.doi.org/10.7554/eLife.23608.021 [file elife-23608-supp1.zip › hi-res_adjacency_matrices/beta/not_downsampled/zscore/beta.ave.aal.thr.zscore.r.not_downsampled.png]

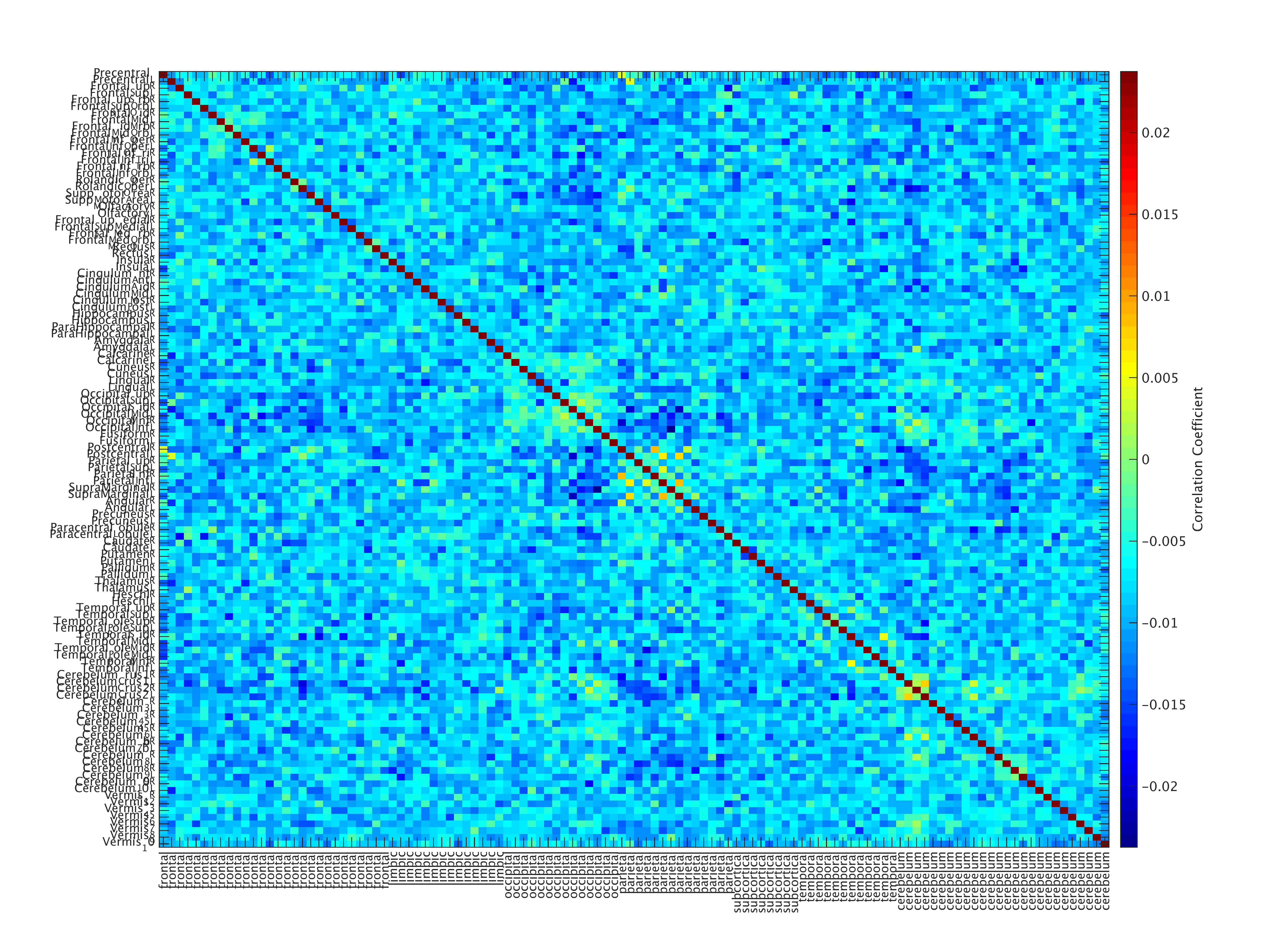

Supplement: Supplementary file 1. — This zip file contains high resolution images of the adjacency matrices for the MEG connectivity analysis suggested by the editor and reviewers. DOI: http://dx.doi.org/10.7554/eLife.23608.021 [file elife-23608-supp1.zip › hi-res_adjacency_matrices/beta/not_downsampled/zscore/beta.ave.aal.thr.zscore.z.not_downsampled.png]

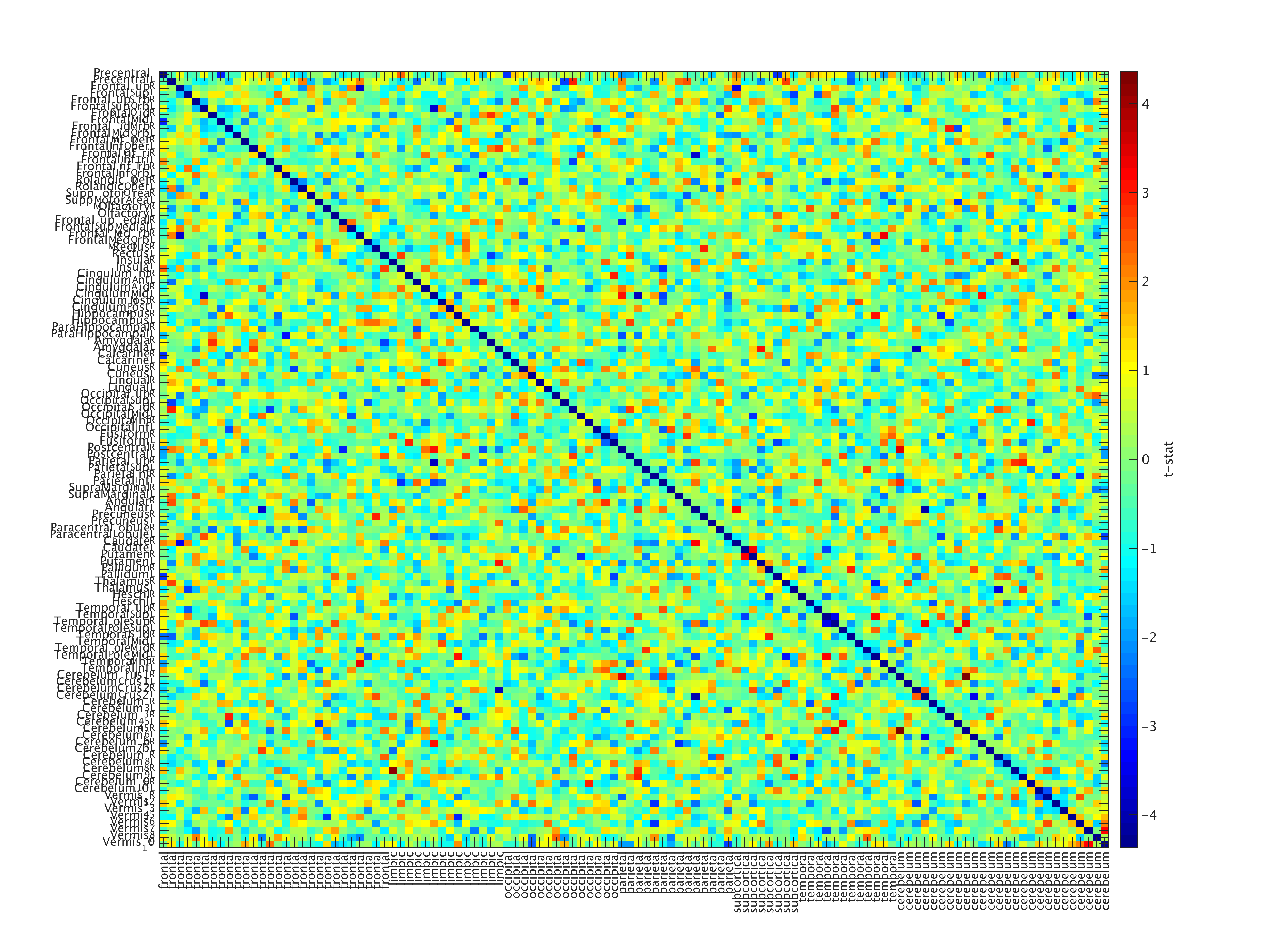

Supplement: Supplementary file 1. — This zip file contains high resolution images of the adjacency matrices for the MEG connectivity analysis suggested by the editor and reviewers. DOI: http://dx.doi.org/10.7554/eLife.23608.021 [file elife-23608-supp1.zip › hi-res_adjacency_matrices/beta/not_downsampled/zscore/beta.tstat.aal.zscore.r.not_downsampled.png]

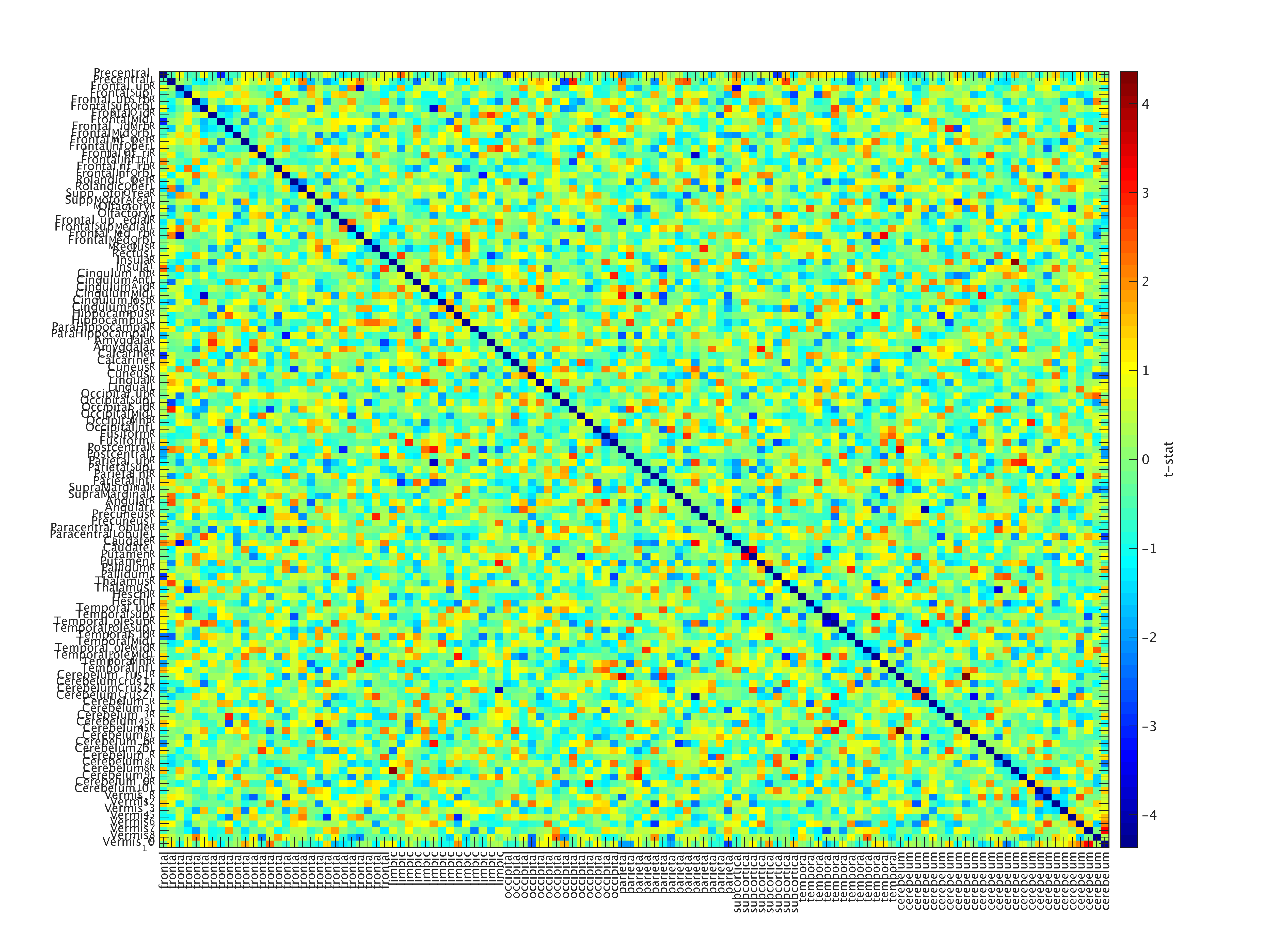

Supplement: Supplementary file 1. — This zip file contains high resolution images of the adjacency matrices for the MEG connectivity analysis suggested by the editor and reviewers. DOI: http://dx.doi.org/10.7554/eLife.23608.021 [file elife-23608-supp1.zip › hi-res_adjacency_matrices/beta/not_downsampled/zscore/beta.tstat.aal.zscore.z.not_downsampled.png]

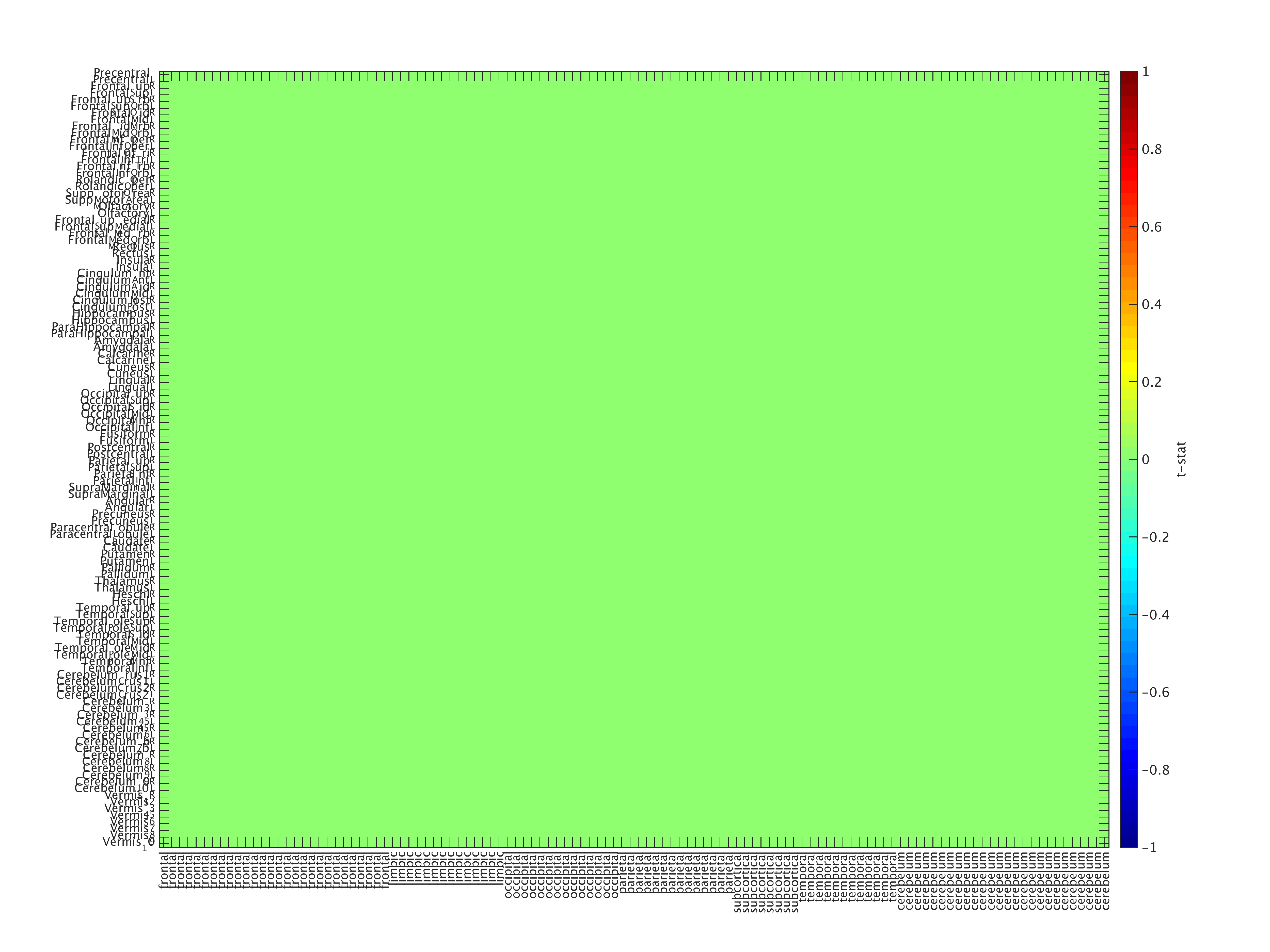

Supplement: Supplementary file 1. — This zip file contains high resolution images of the adjacency matrices for the MEG connectivity analysis suggested by the editor and reviewers. DOI: http://dx.doi.org/10.7554/eLife.23608.021 [file elife-23608-supp1.zip › hi-res_adjacency_matrices/beta/not_downsampled/zscore/beta.t-thresh.aal.zscore.r.not_downsampled.png]

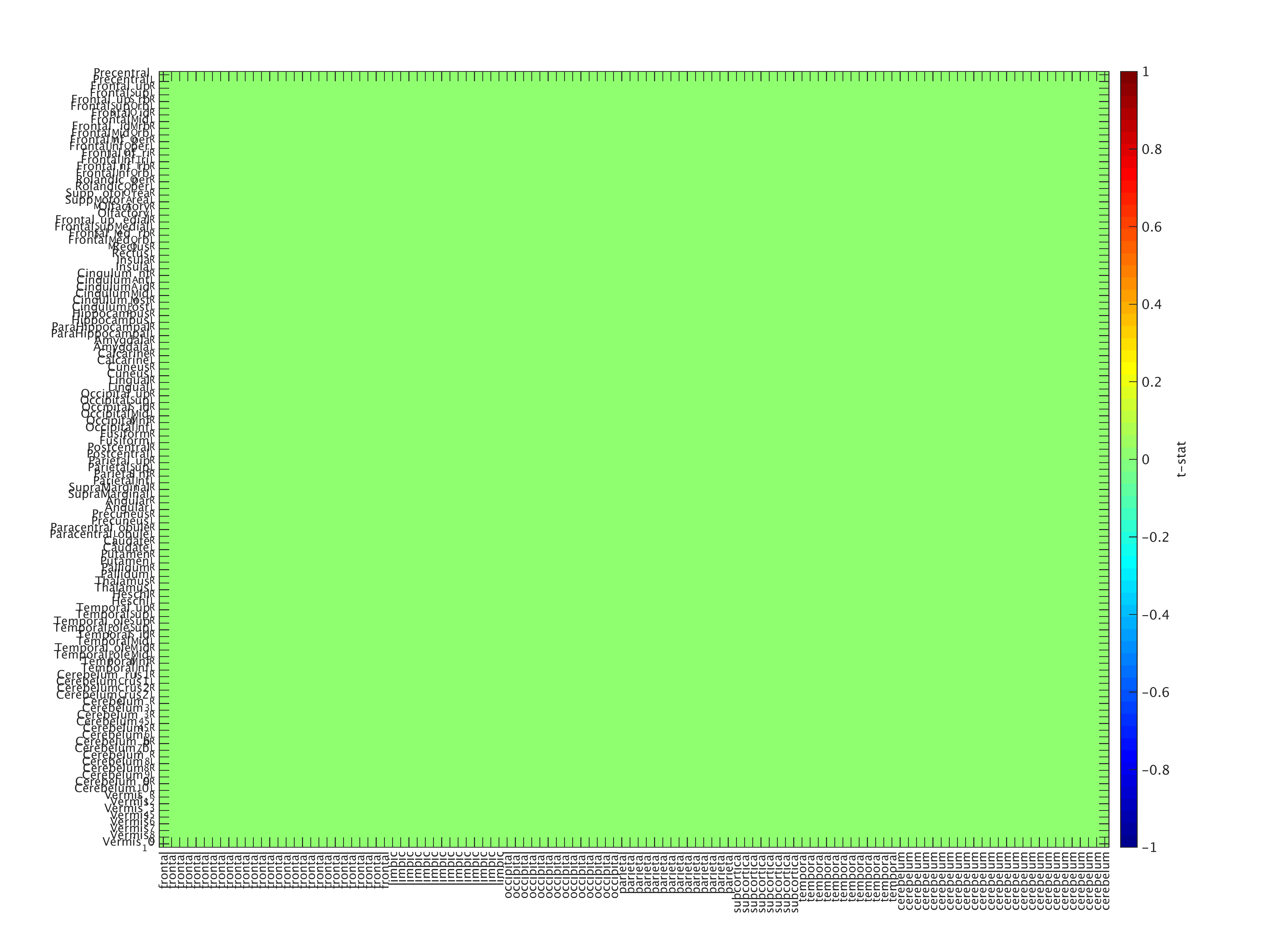

Supplement: Supplementary file 1. — This zip file contains high resolution images of the adjacency matrices for the MEG connectivity analysis suggested by the editor and reviewers. DOI: http://dx.doi.org/10.7554/eLife.23608.021 [file elife-23608-supp1.zip › hi-res_adjacency_matrices/beta/not_downsampled/zscore/beta.t-thresh.aal.zscore.z.not_downsampled.png]

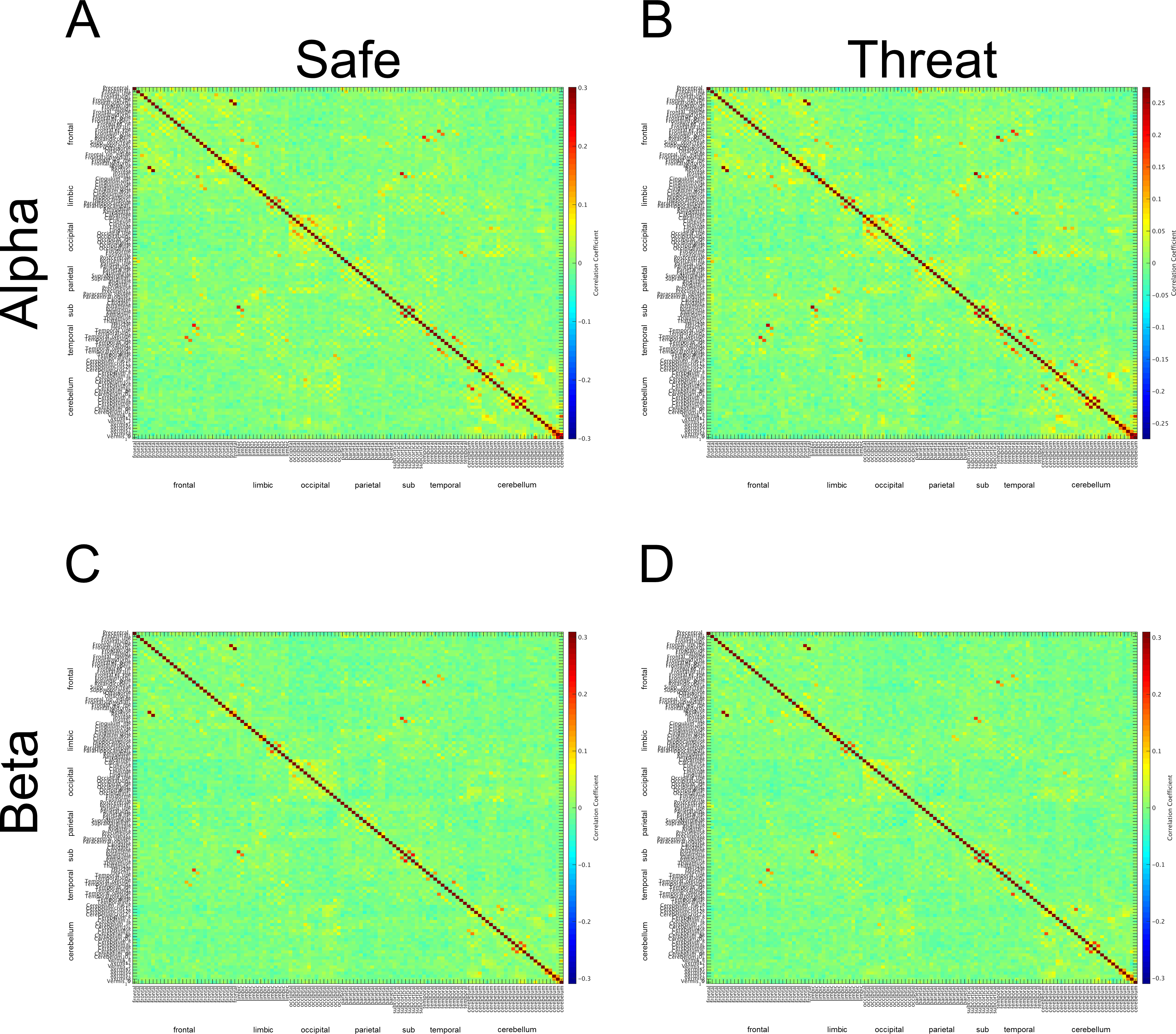

Supplement: Supplementary file 1. — This zip file contains high resolution images of the adjacency matrices for the MEG connectivity analysis suggested by the editor and reviewers. DOI: http://dx.doi.org/10.7554/eLife.23608.021 [file elife-23608-supp1.zip › hi-res_adjacency_matrices/downsampled-raw.tif]

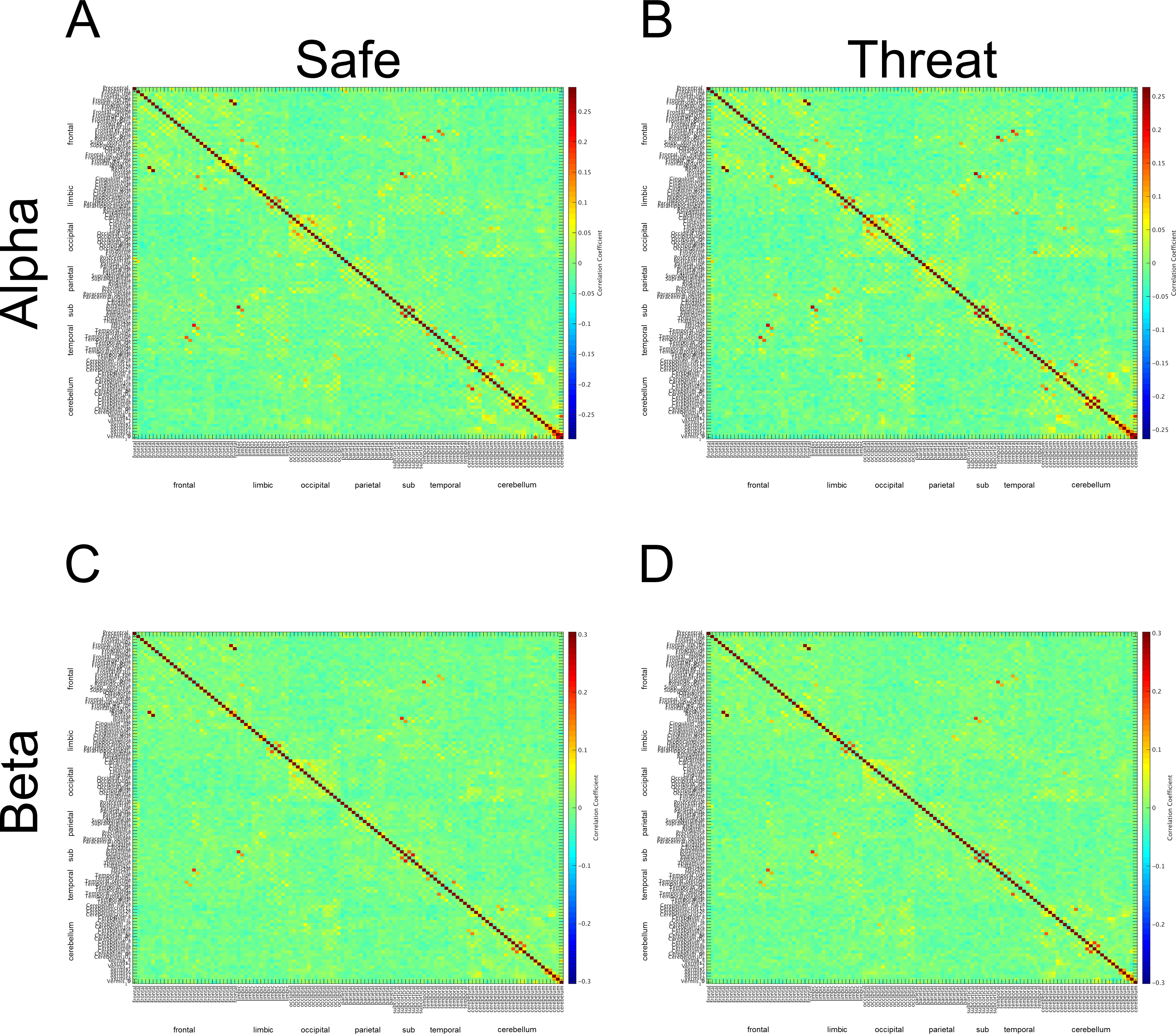

Supplement: Supplementary file 1. — This zip file contains high resolution images of the adjacency matrices for the MEG connectivity analysis suggested by the editor and reviewers. DOI: http://dx.doi.org/10.7554/eLife.23608.021 [file elife-23608-supp1.zip › hi-res_adjacency_matrices/downsampled-zscore.tif]

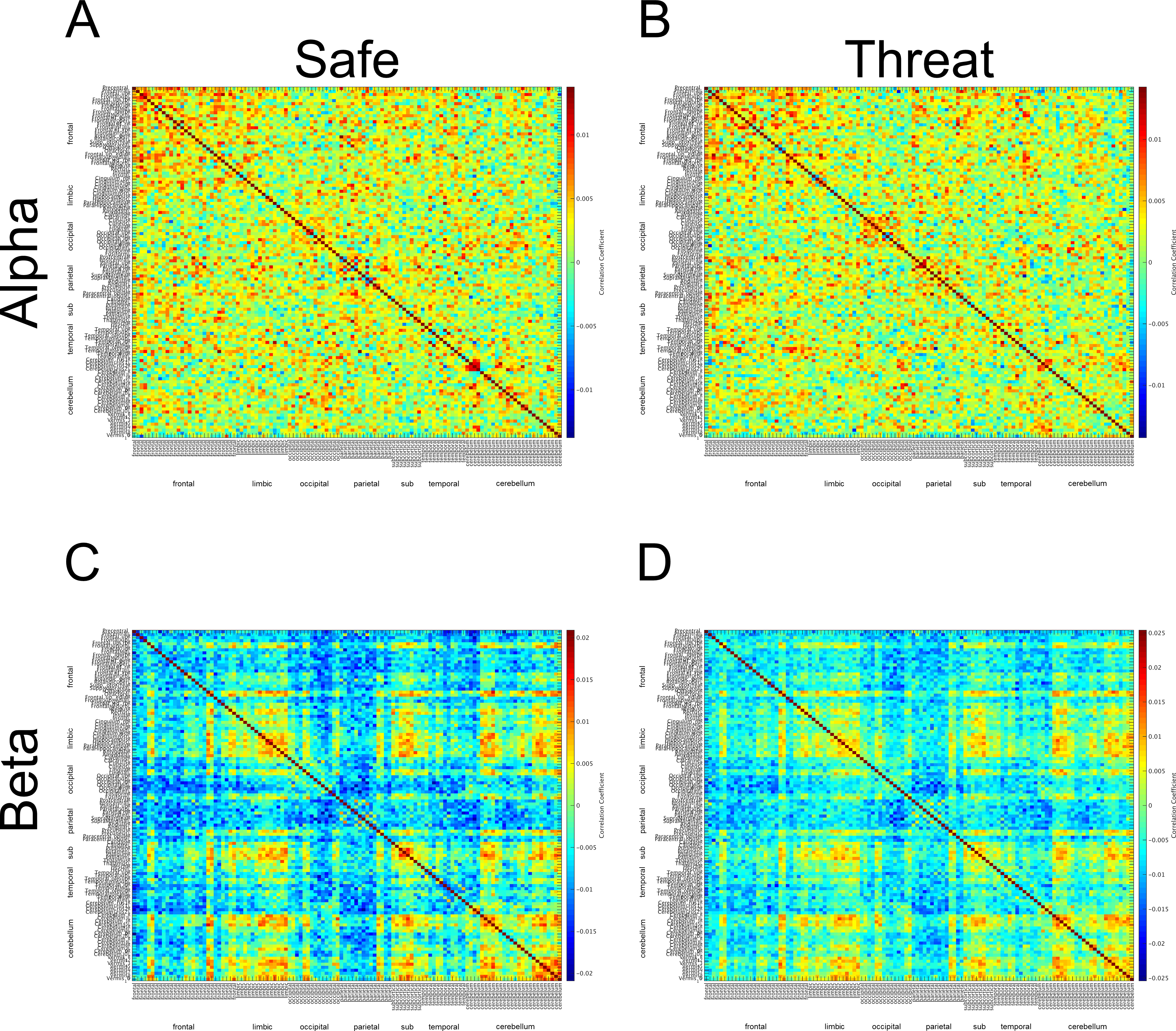

Supplement: Supplementary file 1. — This zip file contains high resolution images of the adjacency matrices for the MEG connectivity analysis suggested by the editor and reviewers. DOI: http://dx.doi.org/10.7554/eLife.23608.021 [file elife-23608-supp1.zip › hi-res_adjacency_matrices/not_downsampled-raw.tif]

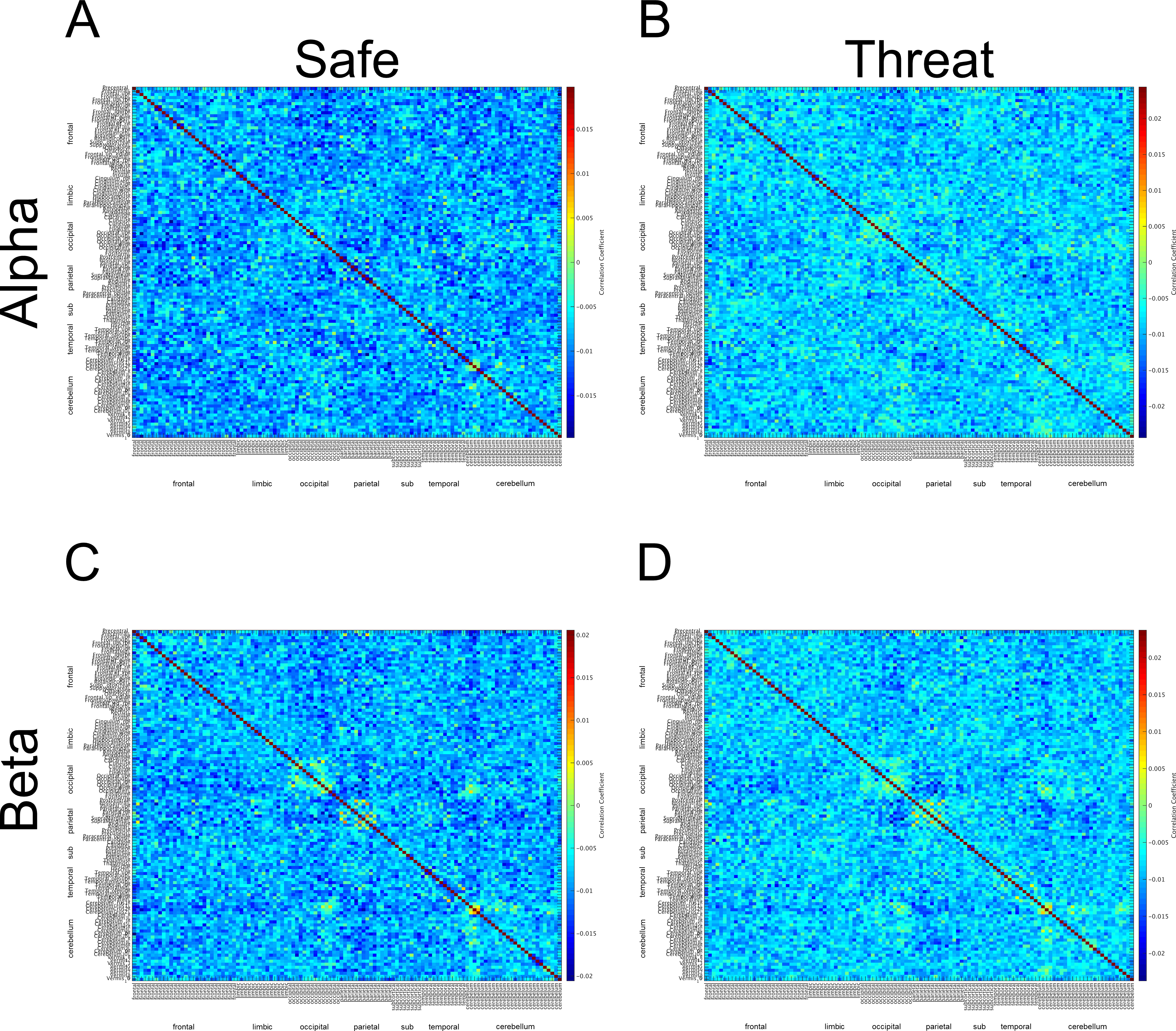

Supplement: Supplementary file 1. — This zip file contains high resolution images of the adjacency matrices for the MEG connectivity analysis suggested by the editor and reviewers. DOI: http://dx.doi.org/10.7554/eLife.23608.021 [file elife-23608-supp1.zip › hi-res_adjacency_matrices/not_downsampled-zscore.tif]
